# Study of Corner and Shape Accuracies in Wire Electro-Discharge Machining of Fin and Gear Profiles and Taper Cutting

**DOI:** 10.3390/mi16050547

**Published:** 2025-04-30

**Authors:** Joshua Adjei-Yeboah, Muhammad Pervej Jahan

**Affiliations:** Department of Mechanical and Manufacturing Engineering, Miami University, Oxford, OH 45056, USA; adjeiyj@miamioh.edu

**Keywords:** wire EDM (WEDM), complex profiles, fins, taper cutting, corner accuracy, Taguchi analysis, optimization

## Abstract

Wire electrical discharge machining (WEDM) enables the production of complex parts with tight tolerances, although maintaining dimensional accuracy in corners and tapers remains challenging due to wire deflection and vibration. This study optimizes WEDM parameters for achieving high accuracy in machining complex geometrical parts and taper cuts in 6061 aluminum alloy using an Excetek W350G WEDM machine with a copper wire electrode. Parameters including wire tension, pulse on-time, pulse off-time, wire feed rate, open circuit voltage, and flushing pressure were varied using a L18 Taguchi orthogonal array and the response graph method to identify optimal cutting conditions. Experimental results indicated that feature-specific optimization is crucial, as different geometrical features (rectangular fins, triangular fins, gears) exhibited varying critical parameters. Key findings highlighted the importance of wire tension and pulse on-time in maintaining cutting accuracy, although at varying levels for specific features. Response graphs demonstrated the effects of major WEDM parameters on corner and profile accuracies, whereas Taguchi analysis provided the optimum settings of parameters for each feature and taper cutting. These findings will help enhance precision, efficiency, and versatility of the WEDM process in machining complex profiles and corners, contributing to precision manufacturing.

## 1. Introduction

Wire electrical discharge machining (WEDM) is a non-traditional manufacturing process that uses an electrically energized wire to progressively erode material from a conductive workpiece [1]. The CNC-guided wire, combined with precisely controlled electrical discharges, facilitates machining of complex two- and three-dimensional profiles with acceptable dimensional tolerances. Major applications include injection molds, turbine blades, precision gears, and surgical implants, which require intricate, high-accuracy features that are difficult to produce through conventional methods [2]. Achieving highly accurate corners and complex geometries with WEDM has significant implications across numerous advanced manufacturing industries. Intricate injection molds with conformal cooling channels, intricate implants and prosthetics tailored to patient anatomy, and rotating turbine components with optimized aerodynamic profiles all stand to benefit from enhanced corner precision in WEDM for tapered cutting operations.

Maintaining accuracy in taper-cutting operations remains a persistent challenge in WEDM. Taper cutting involves an intentional angle applied during the machining motion to compensate for electrode wear. Precise corner geometry is also critical for components with sharp interior angles or features. However, the high temperatures and pressures from electrical discharges can cause small wire deflections. Over longer taper distances, these deflections accumulate and result in inaccuracies in the final workpiece corners and angles [3]. Several factors influence the precision of angled/tapered cuts, including wire diameter, cutting speed, spark voltage, flushing conditions around the wire, and workpiece material properties [3]. The accuracy of machined corners, profiles, and features is a critical aspect determining the quality and performance of components produced by WEDM. Therefore, enhancing corner machining accuracy in WEDM has been an active area of research.

Numerical simulation approaches provide effective methodologies for gaining insights into the corner-cutting mechanisms in WEDM. Han et al. [4] developed a novel technique to simulate the relative motion between the wire path and numerical control (NC) path during rough corner cutting. By modeling wire vibration and the geometric relationship between wire and NC trajectories, they could successfully reproduce corner machining processes computationally. Ebisu et al. [5] employed computational fluid dynamics (CFD) to examine the influence of jet flushing on corner accuracy via two mechanisms, debris flushing at the cut zone and hydrodynamic wire deflection. Their analysis revealed significant changes in flow fields and reduced debris accumulation that transiently occurred upon change in cut direction at corners. This coincided with observations of wire deflection towards the inside corner. The results indicate that jet flushing has a significant influence on corner-shape accuracy in WEDM. Just after turning the corner, the transient changes in flow/pressure fields and reduced debris stagnation could exacerbate wire deflection towards the inside corner under the influence of hydrodynamic forces. While jet flushing is crucial for debris removal, excessive forces near the corner may need to be mitigated to improve dimensional precision. Aswin and Mote [6] developed numerical models to predict tip deformation during WEDM of narrow angles, capturing the dominant contribution of transient thermal stresses. The model was able to capture the dominant contribution of thermal effects to the tip deformation observed experimentally. However, the model underestimated the total deformation, suggesting additional deformation mechanisms like Lorentz forces were not fully accounted for. In another paper, the same group of authors investigated the geometric error and thermal deformation of thin wall structures during a WEDM process by developing three different models. They also conducted experiments to validate model predictions. Their findings showed that the coupled thermo-mechanical model and kerf-width model accurately captured geometrical errors during WEDM of thin walls. A significant overcut error due to kerf formation was predicted and observed. Also, distinct thermal cycles and gradients formed due to transient heat dissipation influenced by the kerf and cooling rates reduced on the wall crest due to kerf insulation, enhancing thermal deformation. Experimentally measured deformation correlated well with model predictions, validating their approach [7].

Besides numerical studies, extensive experimental investigations have analyzed the effects of WEDM parameters, wire properties, use of powder additives in dielectric, etc. on corner errors. Kumar et al. [8] compared performance of zinc-coated brass wire and uncoated wire during WEDM of Inconel 718 alloy, finding significantly reduced corner error and improved surface finish with the coated wire. The zinc coating on the brass wire electrode lowered its melting point and improved cooling, leading to better surface quality during the machining process. The coating also enhances debris removal, reducing thermal damage. Process parameters like higher wire tension and flushing pressure also reduced corner error by limiting wire vibration. On the other hand, an increase in pulse on-time and discharge current improved the material removal rate (MRR) but increased corner error. The results showed the effectiveness of zinc coating on the brass wire electrode, whilst also indicating that proper control of process parameters like wire tension and flushing pressure helps control wire vibration and position, minimizing geometrical errors at corners. While high energy parameters boost MRR, they need optimization to avoid degradation of machining accuracy. Saravanan et al. [9] optimized parameters like voltage, current, pulse times, wire size, and tension to maximize corner accuracy along with MRR and surface finish during WEDM machining of titanium grade 2. Their results showed that gap voltage, wire tension, and wire diameter were most influential. Manoj et al. [10] examined the effect of machining parameters like cutting speed on corner accuracies like corner radius and corner error during profiling of triangular shaped slots on Hastelloy X using WEDM. The slots were machined at 0° and 30° slant angles, and the cutting speed was recorded at different parameters and angles. They found that cutting speed decreased with an increase in slant angle due to increased cutting thickness. Corner inaccuracies increased due to higher wire deflection. The corners had lower radii and errors at lowest cutting speed. Corner radius increased with wire guide distance, wire offset, and cutting speed override, but the effect of corner dwell time varied with angle. Chakraborty et al. [11,12] conducted studies utilizing powder mixed WEDM of Ti6Al4V alloy with B_4_C, SiC, Al_2_O_3_ additions and achieved up to 43% lower corner error compared to plain dielectric. The powder particles facilitated wider spark gaps and continuous discharges at corners owing to their high thermal conductivity. Roan et al. [13,14] carried out an experimental study investigating the influence of non-electrical parameters on corner accuracies during WEDM of titanium alloy and concluded that optimized wire feed rate and wire tension play important roles in determining the corner accuracy during the machining of small kerfs and corners in complex profiles. The same group of authors proposed that identifying wire lag at the corners and compensating wire lag by modifying G-code based on a wire-lag model can minimize inaccuracy at the corner [15].

Multiple investigations employed statistical techniques like response surface methodology (RSM) and gray relational analysis (GRA) for multi-objective optimization of corner errors and other outputs [9,11]. Hybrid GRA with principal component analysis (PCA) outperformed RSM optimization in the study by Chakraborty et al. [11] indicating its superiority for handling complex parametric relationships. Sanchez et al. [16] adopted an alternative strategy of limiting cutting feed rate using controlled pulse off-times for improving corner accuracy after initial roughing. Though initial tests produced substantial reductions in error, the results were non-uniform for different corner zones. This highlighted the need for variable and adaptive feed rate control around corners based on local geometric analysis. A prevalent focus of WEDM gear cutting research has been investigating the effects of key process parameters and optimizing settings to improve productivity and quality. Mohapatra and Sahoo [17] optimized WEDM parameters, including pulse duration, pulse interval, and wire tension, for an Inconel 718 workpiece using the TOPSIS multi-criteria decision-making technique. They obtained a maximum MRR of 1.735 mm^3^/min and a minimum kerf width of 0.602 mm at optimal parameter settings of pulse on-time 119 μs, pulse off-time 57 μs, and wire tension 4 kg-f, enabled by a comprehensive Taguchi experimental design and statistical analysis, with pulse on-time being the most significant factor affecting the response. Mohapatra et al. [18] likewise applied Taguchi and gray-based Multi-Objective Optimization on the Basis of Ratio Analysis (MOORA) methods to determine the best parametric configuration using brass and coated wire electrodes on copper gears. Their analysis found pulse duration (pulse on-time) as the most dominant parameter influencing both MRR and kerf width in both types of wire electrodes. Several other studies have also effectively utilized Taguchi arrays or other design of experiments approaches combined with optimization techniques like desirability functions and principal component analysis to systematically ascertain the WEDM parameters yielding improved gear cutting performance [19,20,21]. The key parameters analyzed have included pulse duration, pulse interval, peak current, voltage, and wire feed/tension, with objectives ranging from maximizing MRR and minimizing surface roughness and kerf width to reducing cutting time or tool wear. The findings collectively demonstrate that by methodically varying WEDM settings through planned experiments and applying multi-objective optimization methods, substantial improvements in gear quality and productivity can be obtained over default parameters.

Beyond optimization, research efforts have also focused on experimentally evaluating the practical cutting performance and finished gear quality achieved with WEDM to demonstrate its feasibility as an alternative production method. Sari et al. [22] compared the resulting metallurgical, geometrical, and functional integrity of ground versus WEDM-machined spur gears. Despite similar initial surface roughness, microstructure, and residual stress conditions, WEDM-finished gears lasted 228% longer in rolling contact fatigue tests, surviving over 21 million cycles without failure. The randomized WEDM crater topography induced beneficial running-in behavior and wear resistance compared to traditional grinding. Likewise, Mohapatra et al. [23] developed a computational model in ANSYS (ANSYS 19.0) to simulate thermal and structural behavior in the wire electrode and workpiece during sparking. Their model results for temperature distribution and equivalent stress regions, further supported by XRD analysis of phases formed, provided insights into the complex material transformations occurring from the thermal cycles in WEDM.

A key consideration in assessing WEDM for gear production is examining the dimensional accuracy and precision achieved. Yusron et al. [24] analyzed profile errors along the involute curve section, finding increased pulse duration and discharge energy exacerbated negative deviations, while higher wire tension helped minimize distortion effects. Meanwhile, Wang et al. [25] addressed miniaturization challenges in fabricating micro-scale gears, developing a two-stage strategy with an intermediate self-centering fixture to improve location precision and achieve intact, conformal profiles. Their approach produced micro-gears with <1.5 μm dimensional errors and surface roughness around 0.9 μm, demonstrating that WEDM can manufacture complex micro-components given appropriate fixturing and process refinements. Beyond conventional WEDM, some studies have proposed integrating wire EDM with other processes in hybrid manufacturing set-ups to leverage potential synergies to enhance profile accuracies. Chen et al. [26] combined WEDM and precision forging, first using WEDM to fabricate micro-gear dies, then hot-forging gears with the dies to boost productivity over solo WEDM by 3.7 times while retaining precision. The modular hybrid process enabled rapid, low-cost micro-gear production. Huertas-Talón et al. [27] likewise programmed WEDM through generated MATLAB (MATLAB 7.10) gear geometry code, facilitating direct transition from design to machining difficult titanium alloys.

Several researchers have applied multi-objective optimization methods to simultaneously improve multiple performance characteristics like angular error, surface roughness, and cutting speed during WEDM taper cutting. Nayak and Mahapatra [28] utilized the utility concept along with the analytic hierarchy process to convert multi-responses into a single optimized utility index. Verma et al. [29] employed Taguchi’s gray relational analysis (TGRA) to optimize pulse on-time, wire tension, wire feed rate, and taper angle for maximizing material removal rate while minimizing taper error and surface roughness. In addition to traditional experimental techniques like Taguchi design, researchers have explored intelligent modeling approaches. Nayak and Mahapatra [30] developed a support vector regression (SVR) model that accurately predicted angular error based on process parameters. Nayak et al. [31] used genetic programming to model complex, non-linear relationships and predict angular error and surface roughness during taper cutting. Kiran et al. [32] developed a regression model using response surface methodology (RSM) and Central Composite Design (CCD) involving three variables (taper angle, part thickness, and servo voltage) at five levels to investigate the effect of the process parameters on responses such as cutting speed and angular error in WEDM taper cutting of AISI D2 tool steel. The results showed that part thickness played a major role in affecting both cutting speed and angular error. As the thickness increased, it led to more interactions between wire and workpiece, increasing machining time and angular deviations. Servo voltage also significantly impacted the responses and needs to be optimized based on other parameters. Some studies have focused on understanding and modeling the underlying mechanics that influence taper accuracy. Sanchez et al. [33] combined design of experiments (DoE) with finite element modeling (FEM) to predict angular error, accounting for non-linear wire deformation effects. Kinoshita et al. [34] proposed a new wire guide that prevents excessive wire bending to improve taper accuracy for steep inclinations.

Investigators have also examined innovative taper cutting methods. Bellotti et al. [35] demonstrated the feasibility of a twin static wire electrical discharge grinding (TS-WEDG) process for controllable taper micro rod fabrication. Jia et al. [36] employed a twin-mirroring-wire tangential feed EDG technique and optimized parameters to reduce taper in high aspect ratio micro-shafts. Furthermore, researchers have analyzed the effects of parameters like taper angle, part thickness, and machining settings on responses such as angular error, surface integrity, and cutting rate for different workpiece materials. Patil and Chanmanwar [37] found taper angle significantly influenced angular error during WEDM taper cutting of titanium. Although Kiran et al. [22] found out in their regression model using RSM that taper angle had the least significant effect on angular error among part thickness and servo voltage, angular error increased with increase in taper angle and part thickness. It initially increased and then decreased with an increase in servo voltage based on the taper angle. Joy et al. [38] studied how cutting speed and recast layer thickness varied with angle of cut in angular machining of Hastelloy X using a slant taper fixture. Gao et al. [39] developed a kinematic model and an accuracy model considering geometric errors for a proposed cradle-type WEDM configuration for machining large tapers. Sahu et al. [40] applied multi-objective particle swarm optimization along with non-linear modeling for simultaneous minimization of angular error and surface roughness. Nayak et al. [41] used the maximum deviation method integrated with Taguchi design to optimize a composite score combining multiple responses like white layer thickness and cutting rate.

While the literature review covers extensive research efforts in WEDM, there is still room for further investigation and advancement. One potential research area that could be addressed is the combined study of corner accuracy, taper cutting, and wire vibration effects for complex geometries. Most existing studies focus on these aspects individually or in limited combinations, but an integrated analysis considering the interplay of these factors could provide valuable insights. Furthermore, while the review mentions the application of WEDM for gear cutting, there is limited information specifically on the machining of tapered gear profiles using WEDM. Investigating the challenges, accuracy requirements, and potential solutions for producing tapered gears with WEDM could be a novel contribution to the field. To this end, this study aims to investigate the dimensional accuracy in straight and taper cutting operations, with a focus on internal and external angles, widths and lengths, taper angle, and curves and heights by means of gear functional parts, and to develop optimized conditions that could be implemented to enhance the dimensional accuracy as needed. The test-part geometry includes various features such as internal and external angles, cooling fin geometries, and gear profiles, allowing for a comprehensive evaluation of WEDM performance in machining complex shapes.

## 2. Experimental Details

### 2.1. Experimental Setup, Materials, and Parameters

**Figure 1:**
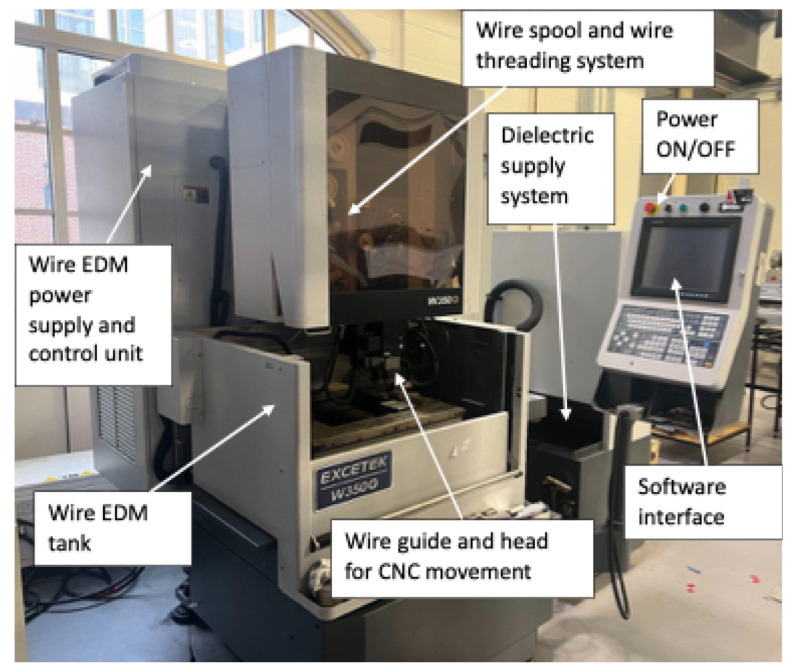
Excetek W350G WEDM Equipment.

For the current study, 6061 aluminum alloy was selected as the workpiece material. A 9.6 mm thick workpiece was considered to investigate the optimum WEDM parameter for corner accuracy of complex profiles and taper cut. A copper wire with a diameter of 0.25 mm (0.01 inch) was used as the tool electrode for the WEDM process. The experiments were carried out on an Excetek W350G WEDM machine, shown in Figure 1. This machine uses deionized water as the dielectric medium for the electrical discharge process. The key cutting conditions of the Excetek W350G are listed in Table 1. The machined samples were analyzed using an Olympus SZX-12 stereomicroscope (Takeshi Yamashita, Tokyo, Japan). Imaging processing and dimensional measurements were performed using ImageJ software (v 1.54p).

### 2.2. Design of Experiments

**Figure 2:**
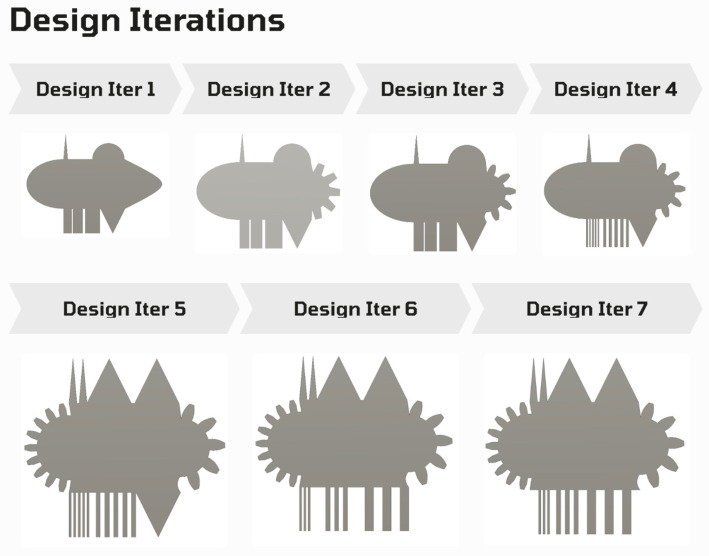
Design iterations to finalize an experimental sample that has multiple complex profiles.

The design of the experiment was set up with varying design iterations to produce one that would capture all complexities covering multiple application areas, as shown in Figure 2. With design iteration 7 being the optimum design used in the initial experiments, it was further modified later to reduce material use and machining time. It embodied three different sets of rectangular cooling fins of sizes 0.5 mm, 1 mm, and 2 mm, two different sets of triangular cooling fins, and two different half-gear profiles. The interval between each rectangular fin in a set was equal to the fin size. This current design provided the opportunity to measure different internal and external angles and compare the same angles among the same geometries or shapes. Also, it was cost-effective in terms of material use and decreased the time to set up an experiment if the various geometries mentioned above were to be machined individually. The tradeoff was the time it took for a machining operation to complete. Finally, this design allowed us to test the efficiency of the WEDM to machine/manufacture different geometries in one run.

To generate the NC code needed to operate the CNC WEDM machine, two approaches were used in this experiment. The CAM software Fusion 360 was used to simulate and generate the toolpaths, which were then post-processed into NC code. The alternative method of manually writing the code directly was also used to confirm accuracy of codes. Since Fusion 360’s CAM module lacks dedicated functionality for WEDM processes and tooling, we had to adapt what was available. The 2D cutting profile operation was used under fabrication in the manufacturing workspace and the laser tool settings were modified to approximately simulate a WEDM manufacturing process. The toolpath simulation data from this makeshift WEDM setup in Fusion 360 CAM could not directly generate wire EDM NC code. To convert the toolpath data into NC code readable by the WEDM machine, a third-party post-processor for WEDM from ‘Baxedm’ was downloaded and integrated into Fusion 360. After using this post-processor to generate the initial NC code from the toolpath simulation, additional manual editing and writing of the code was required to ensure compatibility with the specific WEDM machine used for the experiment and accurate model of the design.

Eighteen (18) experiments were conducted in total, considering 6 factors, 5 factors having 3 levels and 1 factor having 2 levels, as shown in Table 2. The goal was to employ the Taguchi orthogonal array (OA) to investigate effects of non-electrical and electrical parameters including wire tension (WT), feed rate (WF), flushing pressure (WA), pulse on-time (ON), pulse off-time (OFF), and open circuit voltage (OV) on the corner and profile accuracy and to compare accuracy and errors across different profiles for both straight and taper cuts to determine which parameter combination produces the optimum parameter settings that enhance accuracy. All other parameters were kept constant throughout the experiments. Wire tension affects the straightness and stability of the wire, influencing cutting accuracy, surface finish, and wire breakage rates. Feed rate determines the speed of machining and affects material removal rate and productivity, influencing surface roughness and dimensional accuracy. Flushing pressure controls the removal of debris from the cutting zone, affecting the stability of the electrical discharge and influencing surface finish and machining speed. Pulse on-time determines the duration of each spark, affecting material removal rate and surface roughness and influencing tool wear and heat-affected zone. Pulse off-time allows for flushing and cooling between sparks, affecting machining stability and wire breakage and influencing surface finish and dimensional accuracy. Open circuit voltage affects the intensity of the electrical discharge, influencing material removal rate and surface quality and impacting energy efficiency of the process [42,43]. By optimizing these key parameters, we can strike a balance between machining speed, surface quality, dimensional accuracy, and process stability. Based on the number of factors and levels considered, the Taguchi L18 orthogonal analysis L18 OA was used. Table 3 shows the L18 orthogonal array that gives the breakdown of number of experiments in relation to the factors considered.

### 2.3. Test Part Geometry and Measurements

**Figure 3:**
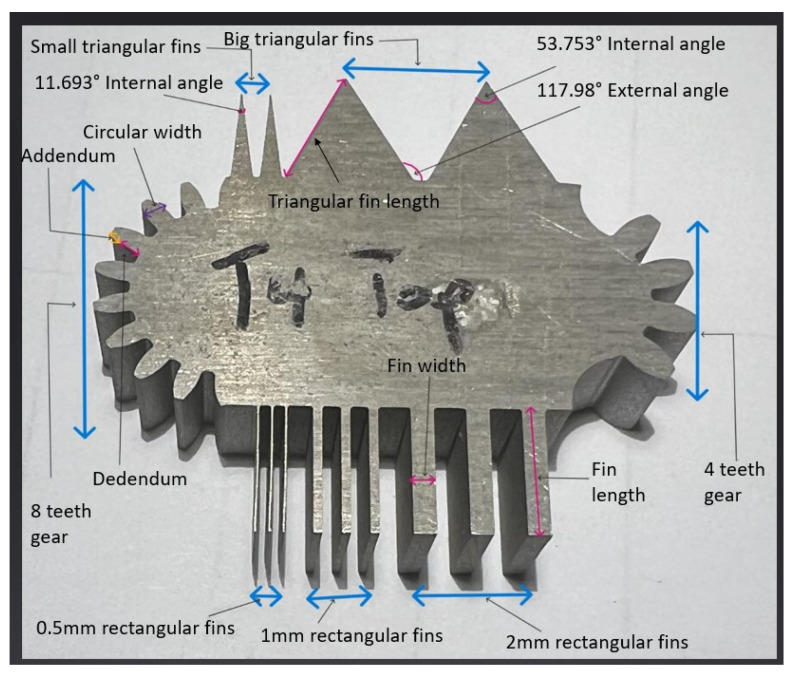
Marked-up image of machined part showing geometrical features and measurements of interest.

**Figure 4:**
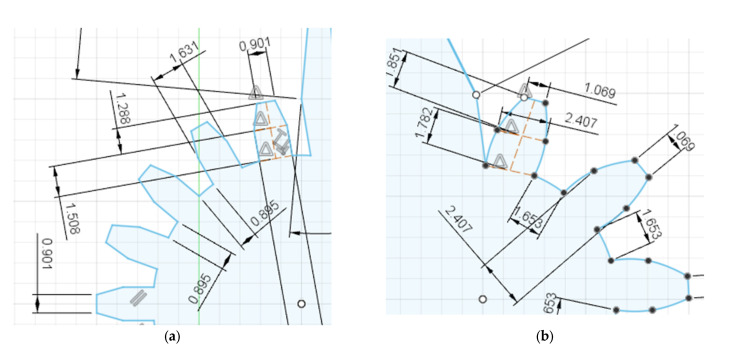
Dimensions for addendum, dedendum, and tooth thickness for (**a**) 8-teeth half gear and (**b**) 4-teeth half gear.

The test part geometry, as shown in Figure 3, was designed in Fusion 360 CAD software to incorporate various features, such as internal/external corners and angles, functional parts of a gear, extremely low dimensions, and straight sections for kerf width measurements. Table 4 and Table 5 shows the target dimensions for rectangular and triangular fins. The rectangular fins were designed to reach extremely low dimensions to test the efficiency of the WEDM to machine to that tight tolerance, especially for taper cut. The length of all rectangular fins was designed to 10 mm, and from right to left, the fins were categorized as 2 mm, 1 mm, and 0.5 mm. Table 6 shows the internal and external angles that make up the part geometry. From left to right, the triangular fins had internal angles of 11.693° and 53.753°. The external angles were measured in between the bigger triangular fins. The sides of the smaller triangular fins measured 9.888 mm and 9.887 mm, whilst those of the bigger triangular fins measured 10.938 mm and 11.030 mm. Two “half” spur gears of different profiles were considered. Table 7 and Figure 4 show the dimensions of gear parts. The addendum and dedendum dimensions of a gear are crucial for maintaining tooth strength, controlling backlash, preventing interference, distributing loads evenly, facilitating lubrication and cooling, and enabling proper manufacturing. Such accuracy and precision are essential for achieving the optimal performance, reliability, and durability of any gear system. Typically, the addendum is lower than the dedendum unless in certain applications, such as turbines and aerospace components, where high speed is required. The half gear on the right with 4 teeth was intentionally modeled while considering such applications.

To compensate for varying width of the fins along its length, measurements were taken at three different positions (top, middle and bottom) for each rectangular fin for both straight and taper cut. The average of these measurements was used as the fin size for each rectangular fin. For the triangular fin length, measurements were taken for both sides of each triangular fin, but only side of each was considered for analysis. It must be noted that some dimensions of interest were not taken because the part was defective, which resulted in missing data, presented later in the results section. Figure 3 shows the marked-up image of machined part showing geometrical features and illustration of how the measurements were taken for each dimension or measurement of interest. Images of all 18 machined samples have been included in the Supplementary Materials in order to save space in the main paper (Please see Figures S1 and S2).

### 2.4. Experimental Procedure

The experimental procedure followed the steps below:

1. The 5-axis Excetek W350G wire EDM machine was initialized, including finding the home position of all axes.
2. A workpiece made of 6061 aluminum alloy with the designated thickness of 9.6 mm was securely clamped onto the machine’s bed/fixture.
3. The edge of the workpiece was located and used to set the X-Y axis zero datum points.
4. The NC code containing the geometry/profile to be cut was transferred to the Excetek control from a flash drive.
5. The WEDM process parameters were verified before running.
6. The WEDM process was executed to cut the test part geometry from the workpiece material.
7. This cutting process was repeated for all the factor level combinations.
8. The machined samples were examined under the Olympus SZX-12 stereomicroscope, and images were captured at various locations, including corners, tapered sections, and straight sections.
9. ImageJ software was used to take dimensional measurements from the images, such as internal/external angles and taper angles.
10. The measurements were recorded, and statistical analysis performed to quantify deviations from the nominal geometry and evaluate the effects of the WEDM parameters on taper-cutting accuracy and corner geometry.

### 2.5. Data Analysis and Evaluation

The data collected from the experiments, including dimensional measurements and deviations from the nominal geometry, were analyzed using the response graph method. The response graph method is one of the many approaches used to analyze data collected from Taguchi orthogonal array (OA)-based experiments. The method uses orthogonal arrays to study the effect of multiple factors on performance and helps in optimizing parameters by analyzing how different levels of the factors (parameters) affect the output response.

The experimental results focus on quantifying and analyzing the deviations in various dimensions (rectangular fin size, triangular fin side length, angles, gear profile dimensions, etc.) for the different electrical and non-electrical parameters and cutting conditions (straight cut and 5° taper cut) by means of calculating the absolute percent deviations (error) for each measured dimension. Since the objective of the analysis is to understand the effects of the aforementioned parameters on the dimensional accuracy of complex profiles and taper cut, this technique was chosen to help identify the most influential factors and determine the optimal factor levels that will minimize the deviations, if any, to achieve measured values as close to the actual values as possible. To conduct parameter optimization using the response graph method, the following procedure was followed for each dimension of interest.

1. Create a response totals table for all factor effects by adding the response variables associated with each of the factor levels, considering replications if the experiment was conducted more than once.
2. Create an average response table for all factor levels by dividing their respective response totals by the total number of observations for that factor level, including observations for replications, if any.
3. Determine the effects of each factor by finding the absolute difference of each of the factor levels.
4. Rank these factor effects, with the highest difference ranked as 1 and so on.
5. Draw the response graph using the average responses.
6. Determine the optimum condition by choosing the lowest mean value of each factor for the case of minimization optimization and vice versa, taking into consideration the ranking to emphasize the effect of the factor.
7. Determine the predicted optimum response by taking the sum of the differences between the average response of the factors in the determined optimum condition and the overall mean response (total of all observations for dimension of interest, including observations for replications, if any, divided by the total number of observations), adding the total to the overall mean response.

## 3. Results and Discussion

### 3.1. Analysis of % Dimensional Accuracy and Percentage Deviations for L18

Table 8 shows the measurements of all the feature dimensions against the target dimensions and percentage deviation. Similar tables have been generated for each of the 18 experiments: T1–T9 for straight cuts and T10–T18 for taper cuts. All the tables have been included in the Supplementary Materials to save space in the journal article. Generally, there are consistent deviations from the intended dimensions across all samples. For rectangular fins, the width measurements show significant deviations, particularly for smaller intended widths. The 0.5 mm fins consistently show the largest percentage deviations, often exceeding 60%. Length measurements for rectangular fins tend to be more accurate, with deviations typically less than 3%, although T2 measurements (Table S2 in the Supplementary Materials) show some higher deviations for 0.5 mm fins. Triangular fins also show notable deviations, with the small triangular fins having larger percentage deviations (around 20%) compared to the big triangular fins (typically less than 5%). Angle measurements show varying levels of deviation, with internal angles of small triangular fins often showing higher percentage deviations compared to other angle measurements. For gear profiles, both eight-teeth and four-teeth gears show deviations in addendum, dedendum, and tooth thickness/width measurements, but these were generally less severe than the deviations seen in fin width measurements. Overall, the measurements shown in Tables S1–S9 in the Supplementary Materials suggests that smaller intended dimensions were more challenging to manufacture accurately for all straight-cut experiments, and there was a consistent trend of under-sizing in most measurements, particularly for fin widths. For T7 measurements (Table S7 in the Supplementary Materials), the 0.5 mm rectangular fins were severely deformed. As such, no measurements could be taken for either the widths or length.

Table 9 presents the measurements of all the feature dimensions against the target dimensions and percentage deviation for taper cut for experiment T10. The measurement data for experiments T10–T18 have been included in the Supplementary Materials to save space in the main article (see Tables S10–S18 in the Supplementary Materials). As can be seen from Table 9, For the 2 mm fins, the calculated taper angles were generally close to the intended 5°, with deviations typically less than 5%. However, the 1 mm fins consistently showed larger deviations from the intended taper angle, often exceeding 10% and sometimes reaching over 25%. The 0.5 mm fins were consistently defective, with measurements not taken, suggesting that manufacturing smaller fins with the desired taper is more challenging. The internal angles of the small triangular fins showed significant deviations, often around 10% or more, while the internal angles of the big triangular fins and the external angles showed smaller deviations, typically less than 4%. Across all samples, there was a consistent trend of undershooting the intended taper angle for the 1 mm fins, indicating difficulty in manufacturing these smaller features. Overall, the measurements shown Tables S10–S18 in the Supplementary Materials indicate that achieving the intended dimensions and angles is more difficult for smaller features, particularly for the 1 mm and 0.5 mm fins.

#### 3.1.1. L18% Deviation Plot Rectangular Fin Width

**Figure 5:**
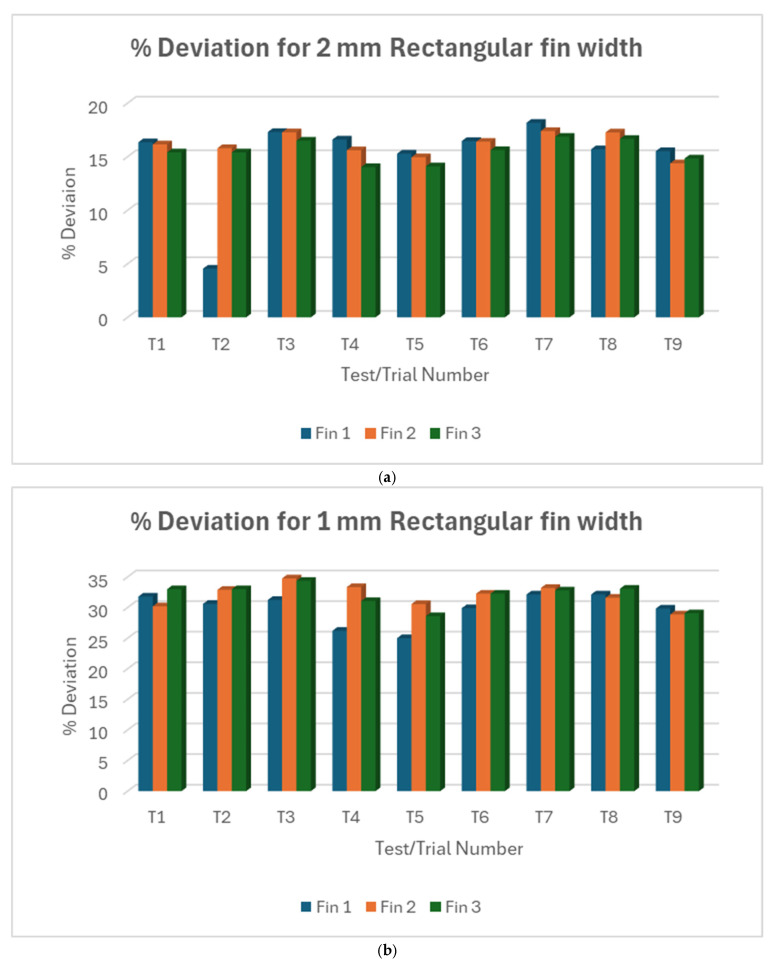

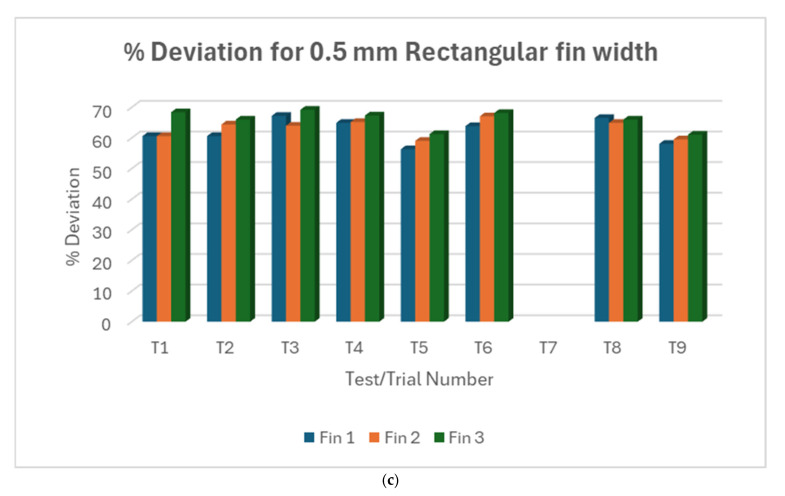
(**a**) Individual fin width % deviation for 2 mm rectangular fin width from T1 to T9, (**b**) individual fin width % deviation for 1 mm rectangular fin width from T1 to T9, (**c**) individual fin width % deviation for 1 mm rectangular fin width from T1 to T9.

**Figure 6:**
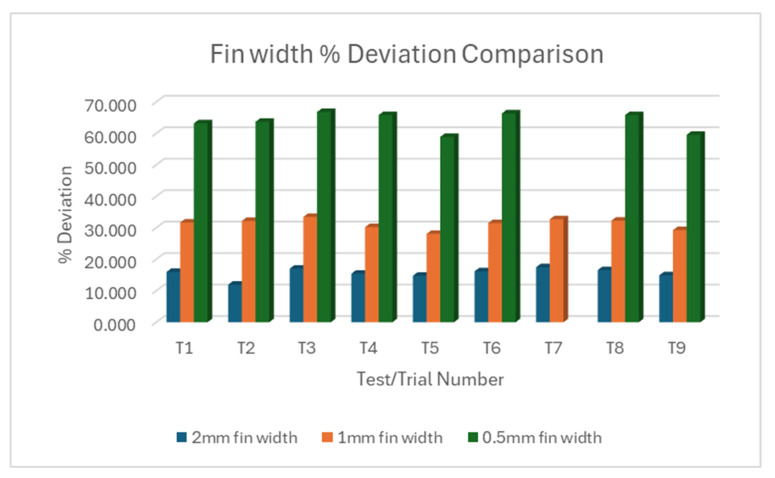
% Deviation comparison of all 3 fin-width sizes from T1 to T9.

Figure 5a–c show variations between individual fins within each trial for 2 mm, 1 mm, and 0.5 mm rectangular fins, respectively. Collectively, T2 showed the lowest deviation for 2 mm rectangular fin width across all nine trials for straight cut. T5 showed the lowest deviation for both 1 mm and 0.5 mm fins. It must be noted that the 0.5 mm rectangular fin was barely machined during trial 7, rendering it defective. Putting all three fin sizes against each other, as shown in Figure 6, the 0.5 mm fin size showed the largest width deviation, followed by the 1 mm fin size, emphasizing the trend of increasing deviation as fin width decreases.

**Figure 7:**
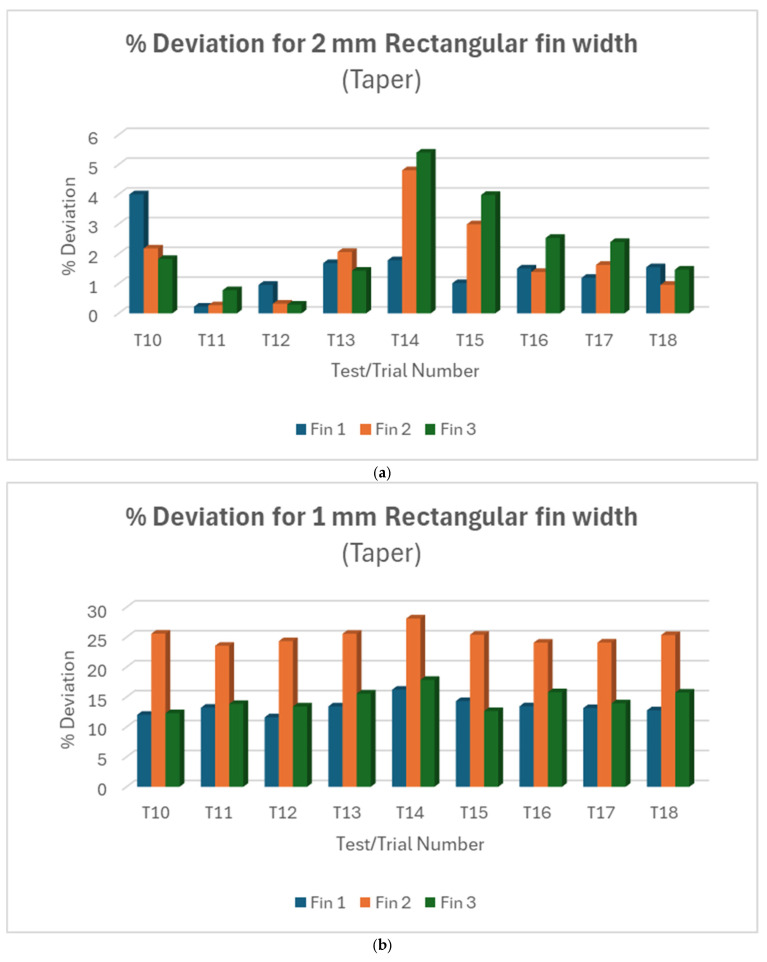
(**a**) Individual fin taper angle % deviation for 2 mm rectangular fin width from T10 to T18, (**b**) individual fin taper angle % deviation for 1 mm rectangular fin width from T10 to T18.

**Figure 8:**
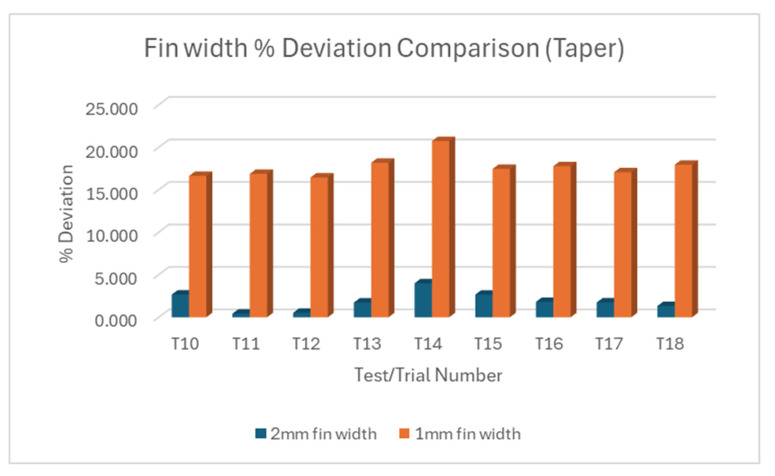
Taper angle % deviation comparison of 2 mm and 1 mm fin-width sizes from T10 to T18.

Like observations made in trials T1–T9 for rectangular fin width, taper angles measured across the 2 mm and 1 mm fin width for T10 to T18 showed variations between individual fins. Nonetheless, Figure 7a showed that the variations were more intense with the 2 mm fin compared to the variations in the 1 mm fin width shown in Figure 7b. Across all the experiments, the deviations in fin 2 were extremely high for taper angle in the 1 mm fin. Collectively, T11 yielded the lowest deviation for taper angle in the 2 mm fin. T12 is found to be the best case for the 1 mm fin, although the deviations were not extremely apart from each other across all the trials. Putting the measured taper angle of the two fin sizes against each other, Figure 8 showed consistent extreme deviations in taper angle in the 1 mm fin width.

In summary, smaller fin widths consistently show larger percentage deviations in both straight and taper cuts. This suggests that achieving accuracy becomes more challenging as feature size decreases. The variability between trials and individual fins within trials increases as the fin width decreases may indicate that the process becomes less stable and predictable for smaller features. The very large deviations for 0.5 mm fins in straight cuts (and the absence of taper cut data and response variable for 0.5 mm fins) suggest that this might be approaching the limit of what can be accurately machined with the current setup.

#### 3.1.2. L18% Deviation Plot Rectangular Fin Length

**Figure 9:**
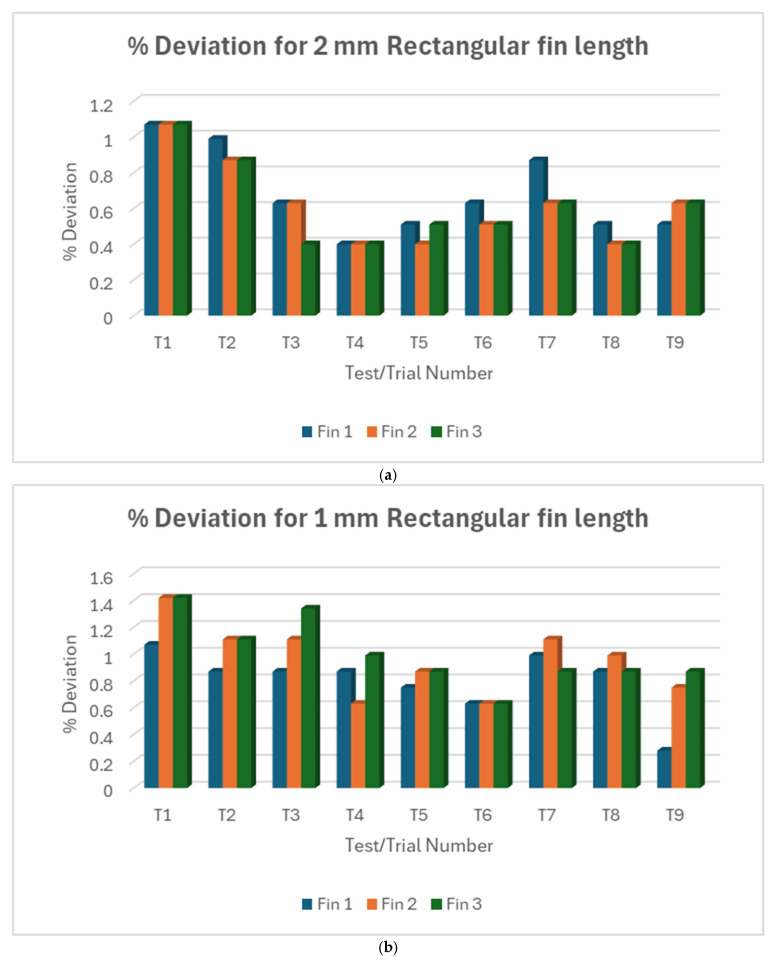

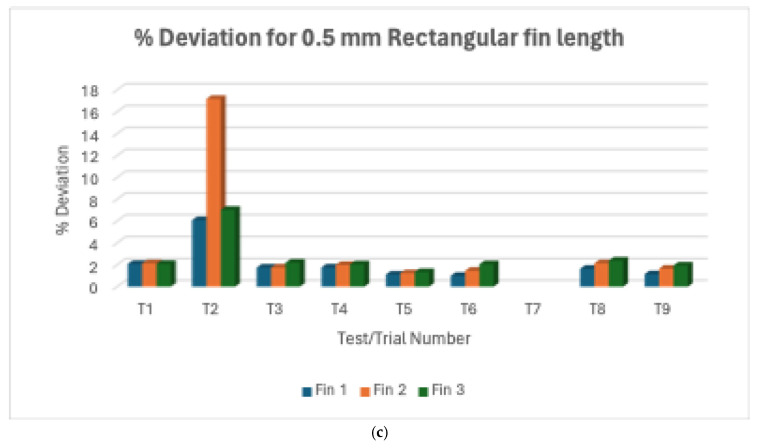
(**a**) Individual fin length % deviation for 2 mm rectangular fin length from T1 to T9, (**b**) individual fin length % deviation for 1 mm rectangular fin length from T1 to T9, (**c**) individual fin length % deviation for 0.5 mm rectangular fin length from T1 to T9.

**Figure 10:**
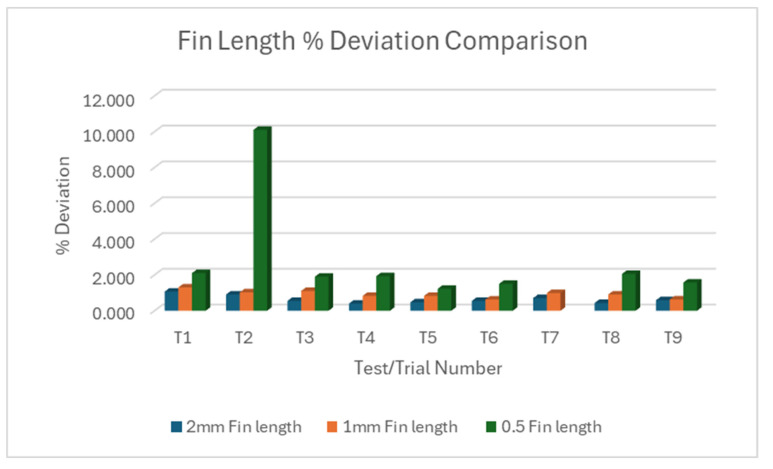
% Deviation comparison of all 3 fin-length sizes from T1 to T9.

Figure 9a shows some variability between individual fins within some trials of the 2 mm fin, although these are small deviations. Figure 9b shows slightly more variability in the 1 mm fin. The pattern of deviations across trials is different from the 2 mm fins, suggesting that factors affecting accuracy might interact differently with different fin sizes. Figure 9c showed more variability between trials and between individual fins within trials of the 0.5 mm fin compared to the 1 mm and 2 mm fins. Collectively, T4 has the lowest deviation in the 2 mm fin length, T6 has the lowest for 1 mm fin, and T5 had the lowest for 0.5 mm fin length. Putting all three fin sizes against each other as shown in Figure 10, all fin lengths show relatively small deviations compared to the width measurements. A slight trend of increasing deviation as the fin size decreases is observed. The 0.5 mm fins show the largest spread of deviations, indicating less consistency in achieving the target length for smaller features.

Overall, the length measurements show much better accuracy compared to the width measurements. This suggests that the WEDM process is more capable of achieving accurate cuts and more stable and predictable for controlling length compared to width. Unlike the width measurements, the length measurements show only a slight increase in deviation as the fin size decreases. This indicates that the process is more robust in maintaining length accuracy across different feature sizes.

#### 3.1.3. L18% Deviation Plot Triangular Fin Length

**Figure 11:**
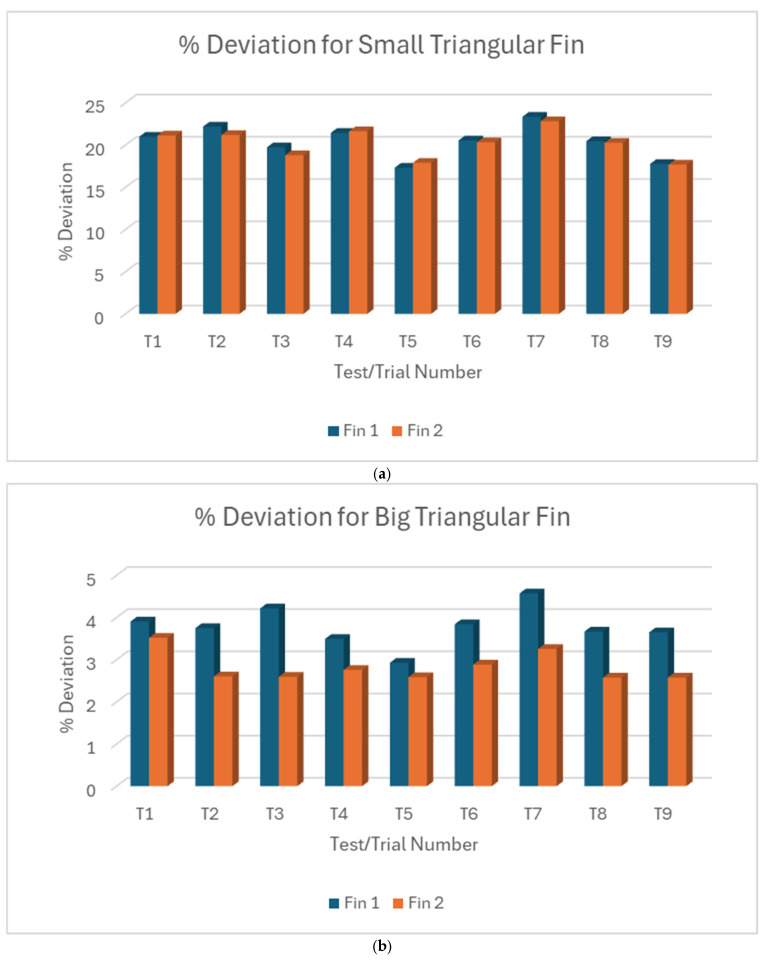
(**a**) Individual fin length % deviation for small triangular fin length from T1 to T9, (**b**) individual fin length % deviation for big triangular fin length from T1 to T9.

**Figure 12:**
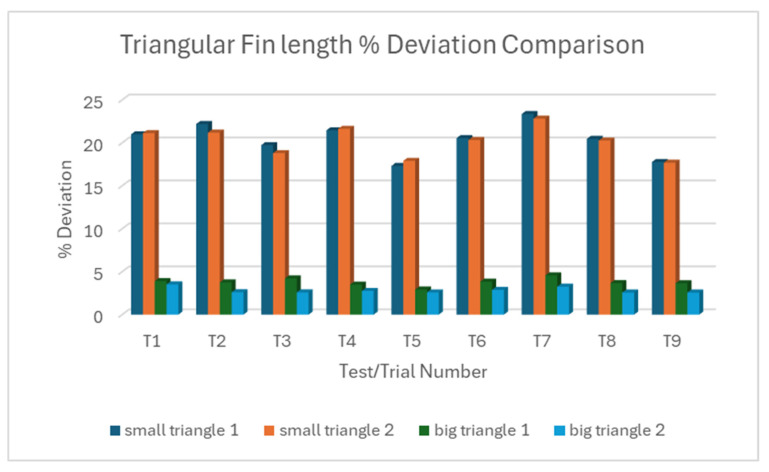
% Deviation comparison of all small and big triangular fin lengths from T1 to T9.

For small triangular fin length, the two individual measurements for each trial often show similar deviations, suggesting consistency within trials but variability between trials compared to the variations in fin length in the big triangular fin, as shown in Figure 11a,b. When they are put against each other as shown in Figure 12, there is a clear and significant difference in the magnitude of deviations between small and big triangular fins. Overall, there is a strong correlation between fin size and machine accuracy. The bigger triangular fins are cut much more accurately than the smaller ones. The significantly larger deviations and variability in the small triangular fins highlight the increased difficulty in accurately machining smaller features. The generally close agreement between the two measurements within each trial, especially in the small triangular fin, indicates good repeatability for individual cuts, even when there is variability between different trials. Compared with the rectangular fins, the deviation pattern suggests that the geometry of the feature and size may influence the machining accuracy.

#### 3.1.4. L18% Deviation Plot Internal and External Angles

**Figure 13:**
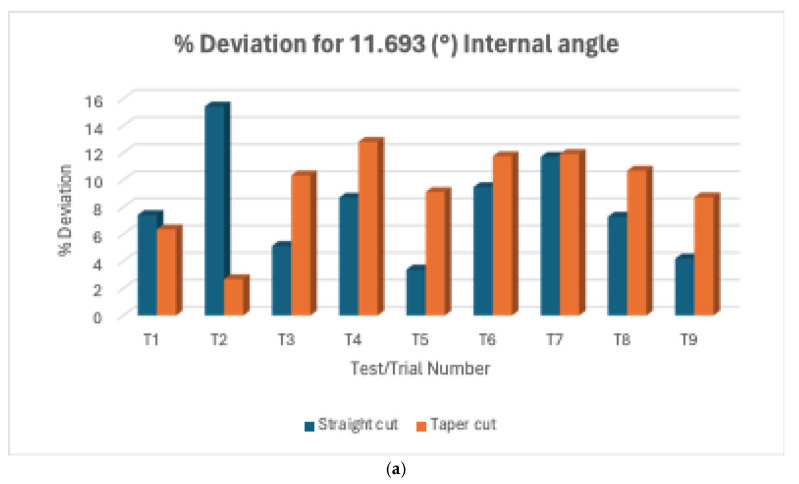

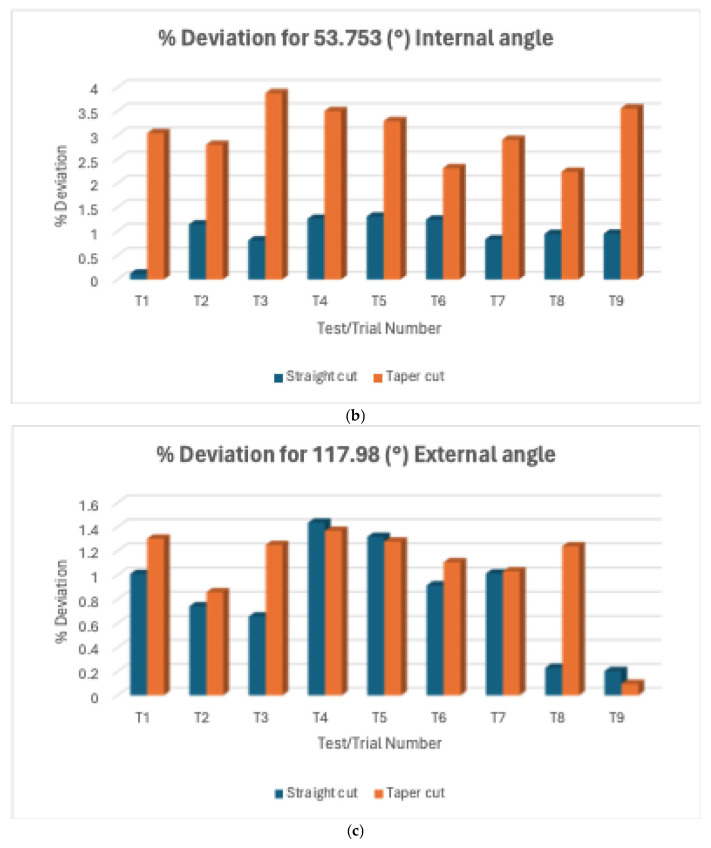
(**a**) % Deviation for 11.693 (°) internal angle from T1 to T9 and T9 to T18, (**b**) % deviation for 53.753 (°) internal angle from T1 to T9 and T9 to T18, (**c**) % deviation for 117.98 (°) external angle from T1 to T9 and T9 to T18 (experimental parameter for T1 = T10 except for T1 = straight cut, T10 = taper cut, and so on).

For internal and external angles, Figure 13a–c show variations in deviation between trials across all experiments (T1 to T9 for straight cut and T10 to T18 for taper cut), signifying the varying effects of the parameter/cutting conditions on these measurements of interest. Overall, the 11.693° internal angle showed the largest deviations and variability for both straight and taper cuts. 53.753° internal angle and 117.98° external angle showed much better accuracy and consistency. Comparative analysis suggests taper cuts consistently showed larger deviations and more variability compared to straight cuts.

#### 3.1.5. L18% Deviation Plot Gear Profile

**Figure 14:**
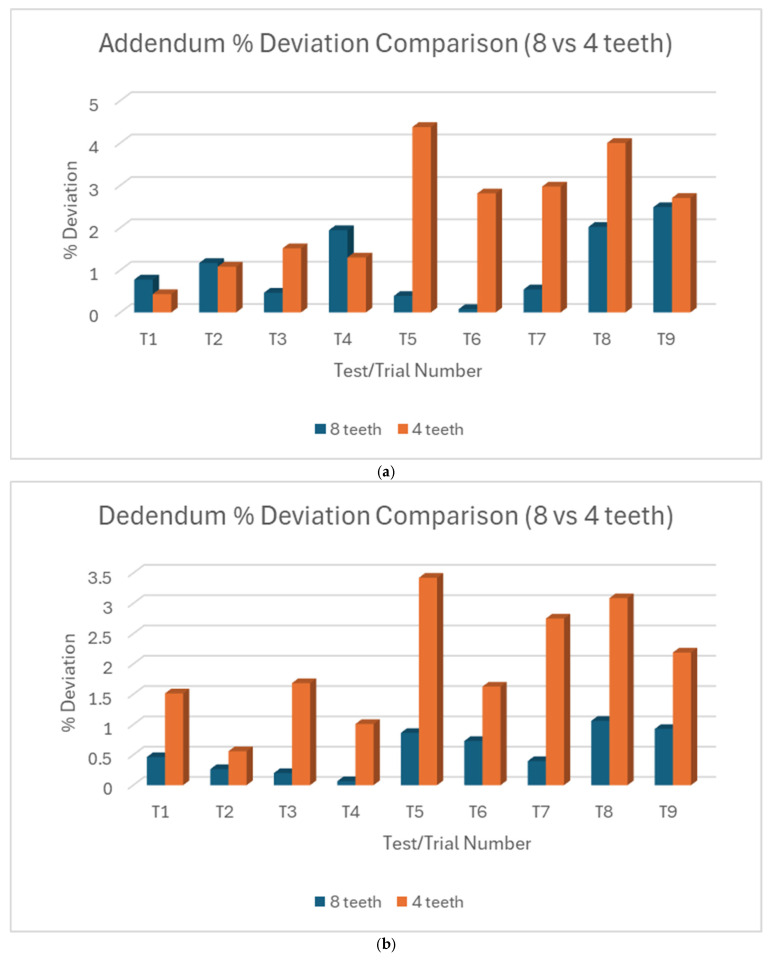

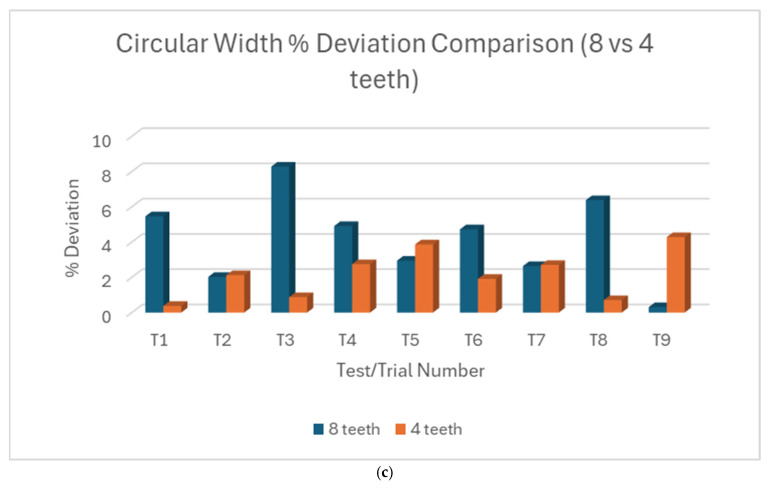
(**a**) Addendum % deviation comparison (8 teeth vs. 4 teeth) from T1 to T9, (**b**) dedendum % deviation comparison (8 teeth vs. 4 teeth) from T1 to T9, (**c**) circular width % deviation comparison (8 teeth vs. 4 teeth) from T1 to T9.

Figure 14a–c show comparison of the addendum, dedendum, and circular width of eight-teeth and four-teeth gear profile cutting using the wire-EDM process. For functional parts of the gear profile, the deviations were relatively low for both eight- and four-teeth gears. Like the observations made in the plots for internal and external angles, Figure 14 shows variations in deviation between trials across all experiments (T1 to T9) for the functional parts of both eight-teeth and four-teeth gear profiles, signifying the varying effects of the parameter/cutting conditions on these measurements of interest. The addendum and dedendum for the eight-teeth gear profile generally performed better in terms of being the lowest in deviation for most of the experiments compared to the circular width, where the four-teeth gear profile outperformed the eight-teeth gear profile.

### 3.2. Data Analysis and Parameter Optimization Using the Response Graph Method

The response graph method is one of the methods used hand in hand with the Taguchi orthogonal array (OA) design of experiments, which provides a visualization of the effect of different factor levels on the response variable, helping to identify optimal parameter settings. The different factors (parameters/cutting conditions) considered in this study included type cut, wire tension (WT), feed rate (WF), flushing pressure (WA), pulse on-time (ON), pulse off-time (OFF), and voltage of open circuit (OV). As noted in methodology, these parameters are key because of how they influence machining speed, accuracy, surface quality, and overall process stability.

Due to different feature sizes even among the same group of measurements of interest, and to adequately account for undercut and overcut, the percent deviation of the measured values (of all measurements of interest) from the nominal values were used as the response variable for this data analysis and optimization process. Since the test part embodies multiple geometrical features, there was the need to group the measured data under the following (also known as the measurements of interest): rectangular fin width, rectangular fin length, triangular fin length, internal and external angle, and gear profile. Once these groups were made, the steps provided in the experimental method were followed to conduct the analysis on optimizing the key factors and factor levels using the response graph method. The analysis of the response tables for various measurements of interest revealed complex relationships between machining parameters and outcomes.

It is important to note, from the measurement table for T7, that the 0.5 mm fin was severely deformed. As such, measurements could not be taken. This missing response posed a significant problem for the analysis and optimization of the 0.5 mm rectangular fin width and length. To handle this, the “NA” in the measurement was replaced with 100 for the response graph method analysis, signifying 100% deviation (error), to still accommodate the impact of the cutting conditions. This eventually increased the average responses for the corresponding factor levels and had a massive influence on the outcome of the analysis for this fin size. The rectangular fin length was affected the most because the percentage deviations were relatively low, with the average being about 2.8%, unlike the rectangular fin width, which had an average deviation of about 64%. Due to this, the addition of the 100% error could easily blend in the responses for the 0.5 mm rectangular fin width, minimally influencing the data. This was different for the 0.5 mm rectangular fin length.

#### 3.2.1. Optimum Conditions, Rectangular Fin Width

Table 10 shows the response table for 2 mm rectangular fin width. Similarly, the response tables for other features are included in the Supplementary Materials in order to save space in the manuscript. Tables S19–S23 in the Supplementary Materials show the response table for rectangular fin width. The most influential factors varied depending on the fin width and cut type. Pulse off-time and pulse on-time were significant for 2 mm straight cuts, while wire tension dominated for 1 mm cuts. In the case of 0.5 mm fins, voltage of open circuit became the most critical factor. Taper cuts showed flushing pressure was most influential for 2 mm fins, while wire tension was most influential for 1 mm fins. Feed rate mostly ranked low in influence across all fin sizes and cut types. The relative importance of voltage of open circuit varies widely between different scenarios.

**Figure 15:**
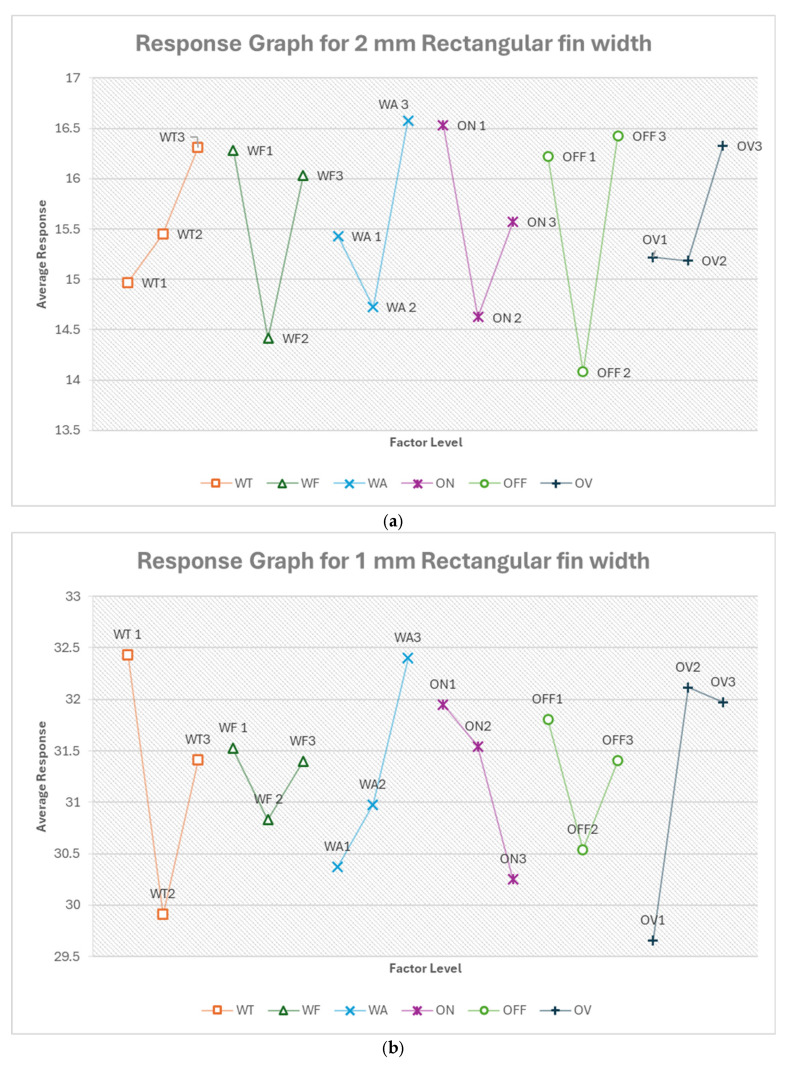

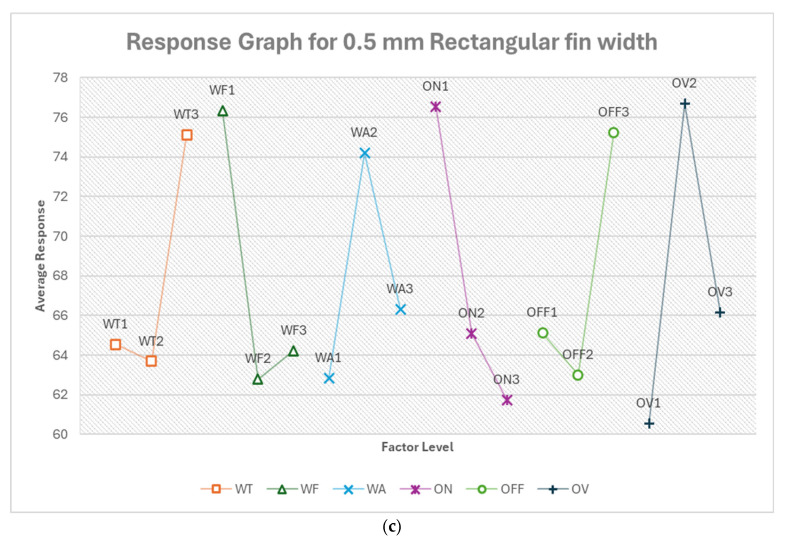
(**a**) Response graph for 2 mm rectangular fin width, (**b**) response graph for 1 mm rectangular fin width, (**c**) response graph for 0.5 mm rectangular fin width. (WT—wire tension, WF—feed rate, WA—flushing pressure, ON—pulse on-time, OFF—pulse off-time, OV—voltage of open circuit).

From Figure 15a, the optimum condition for 2 mm rectangular fin width in the order of their ranking is OFF2 ON2 WF2 WA2 WT1 OV2. The optimum condition prioritizes mid-range settings for most parameters. The most influential factor is pulse off-time (OFF) at level 2 (14 μs), which suggests that a moderate off-time allows for sufficient cooling and flushing without excessively slowing the cutting process. The second-most important factor is pulse on-time (ON) at level 2 (300 ns), indicating that a moderate on-time provides a balance between material removal rate and precision. The mid-range feed rate (WF2, 10.421 m/min) and flushing pressure (WA2, 6) further support this balanced approach. The lower wire tension (WT1, 500 g) may help reduce wire vibration, improving cutting accuracy for the relatively large 2 mm width. The moderate voltage (OV2, 78.421 V) provides sufficient energy for cutting without excessive thermal effects. The predicted optimum response is 10.11% deviation, which is about a 35% improvement in the deviation (error). It also suggests room for improvement but indicates a reasonable level of accuracy for this feature size.

From Figure 15b, the optimum condition for 1 mm rectangular fin width in the order of their ranking is WT2 OV1 WA1 ON3 OFF2 WF2. For this, wire tension becomes the most critical factor, with WT2 (700 g) providing the optimal balance between wire stability and flexibility for this small (compared to 2 mm fin) feature. The higher voltage (OV1, 92.632 V) may help maintain a stable cutting process for this more delicate feature. Lower flushing pressure (WA1, 4) could reduce the risk of deflecting the wire during cutting. The shortest pulse on-time (ON3, 100 ns) combined with moderate pulse off-time (OFF2, 14 μs) suggests a more precise, controlled cutting process suitable for the smaller width. The moderate feed rate (WF2, 10.421 m/min) balances cutting speed and accuracy. The predicted optimum response is 25.317% deviation, which is about a 19% improvement.

From Figure 15c, the optimum condition for 0.5 mm rectangular fin width in the order of their ranking is OV1 ON3 WF2 OFF2 WT2 WA1. For this, voltage becomes the most critical factor, with the highest setting (OV1, 92.632 V) providing the energy needed for precise cutting at this small scale. The shortest pulse on-time (ON3, 100 ns) allows for very controlled material removal, crucial for such a small feature. The moderate feed rate (WF2, 10.421 m/min) and pulse off-time (OFF2, 14 μs) balance speed and precision. The increased wire tension (WT2, 700 g) helps maintain wire stability for this delicate cut. Low flushing pressure (WA1, 4) minimizes the risk of wire deflection. The predicted optimum response is 35.701% deviation, which is about a 47% improvement.

**Figure 16:**
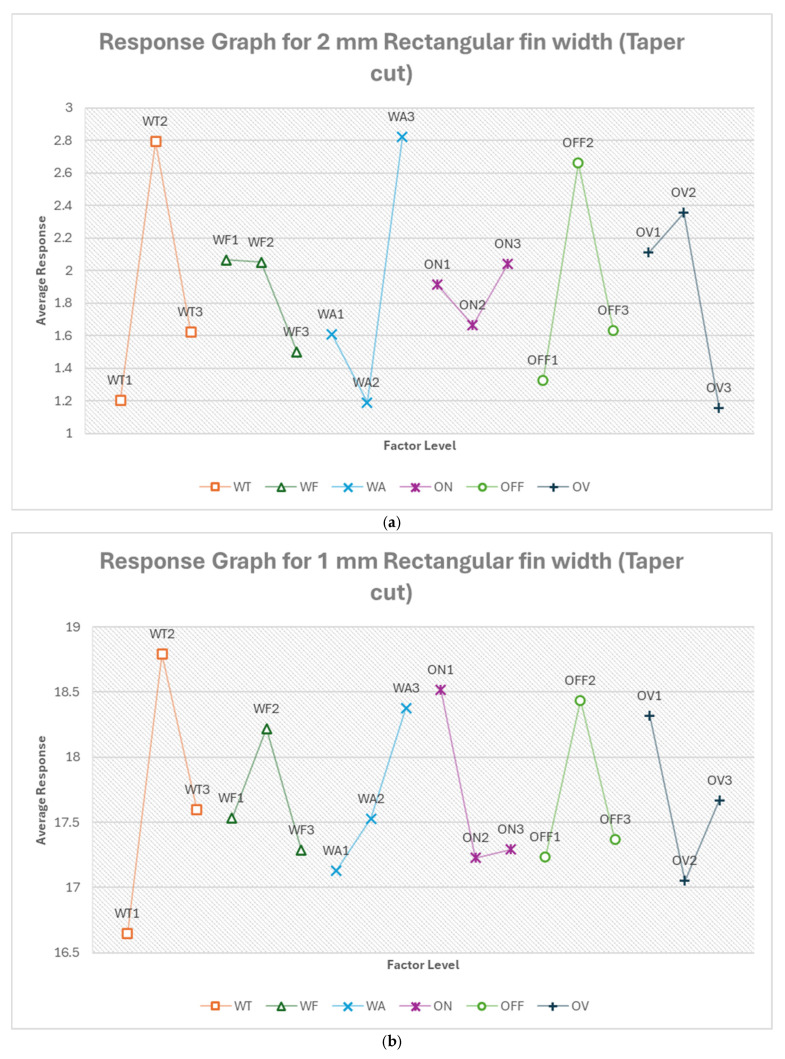
(**a**) Response graph for 2 mm rectangular fin width (taper cut), (**b**) response graph for 1 mm rectangular fin width (taper cut) (WT—wire tension, WF—feed rate, WA—flushing pressure, ON—pulse on-time, OFF—pulse off-time, OV—voltage of open circuit).

From Figure 16a, the optimum condition for 2 mm rectangular fin width (taper cut) in the order of their ranking is WA2 WT1 OFF1 OV3 WF3 ON2. For this, flushing pressure becomes the most critical factor, with a moderate setting (WA2, 6) likely providing optimal debris removal in the angled cut. Lower wire tension (WT1, 500 g) may allow the wire to better conform to the taper angle. The shortest pulse off-time (OFF1, 12 μs) combined with the lowest voltage (OV3, 68.947 V) and highest feed rate (WF3, 14.105 m/min) suggests a more aggressive cutting strategy, possibly to counteract the increased effective material thickness in a taper cut. The moderate pulse on-time (ON2, 300 ns) balances material removal and precision. The predicted optimum response is −1.324%, which is about a 29% improvement.

From Figure 16b, the optimum condition for 1 mm rectangular fin width (taper cut) in the order of their ranking is WT1 ON2 OV2 WA1 OFF1 WF3. For this, wire tension becomes the most critical factor again, as with the straight cut, with the lowest setting (WT1, 500 g) allowing for better wire conformity to the taper angle. The moderate pulse on-time (ON2, 300 ns) and voltage (OV2, 78.421 V) balance energy input for this smaller, angled cut. Lower flushing pressure (WA1, 4) may help prevent wire deflection in this more delicate cut. The shortest pulse off-time (OFF1, 12 μs) and highest feed rate (WF3, 14.105 m/min) suggest a more continuous, faster cutting process, possibly to maintain consistent wire contact with the workpiece throughout the taper. The predicted optimum response is 14.180% deviation, which is about a 20% improvement.

#### 3.2.2. Optimum Conditions, Rectangular Fin Length

Regarding rectangular fin length, wire tension was consistently among the top three most influential factors for all fin sizes and emerged as the most important factor for 2 mm and 1 mm straight cuts. However, for the smaller 0.5 mm fin length, voltage of open circuit became the dominant influence. Feed rate became more influential as the fin size decreased, ranking fifth for 2 mm, second for 1 mm, and second for 0.5 mm fins. Flushing pressure mostly ranked low in influence across all fin sizes. Tables S24–S26 in the Supplementary Materials show the response tables for each fin length.

**Figure 17:**
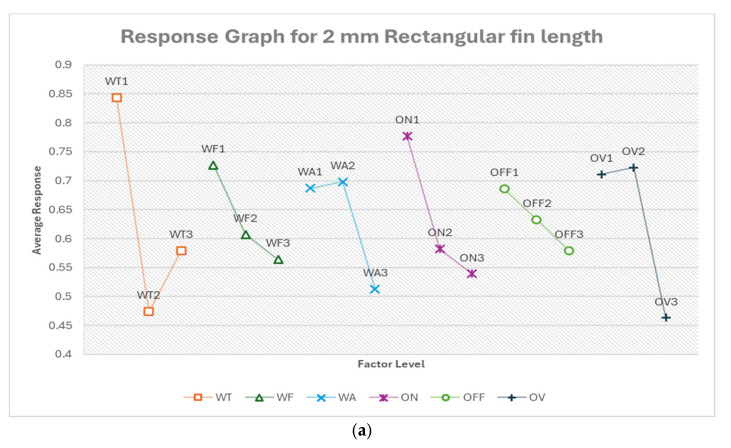

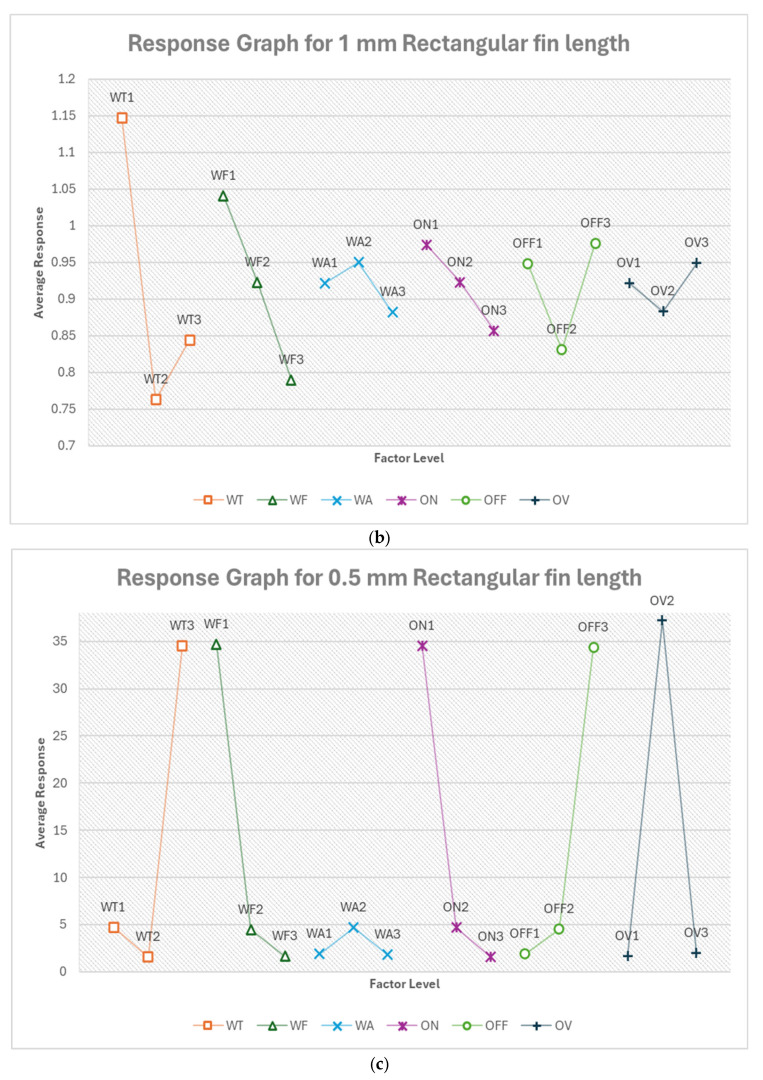
(**a**) Response graph for 2 mm rectangular fin length, (**b**) response graph for 1 mm rectangular fin), (**c**) response graph for 0.5 mm rectangular fin length (WT—wire tension, WF—feed rate, WA—flushing pressure, ON—pulse on-time, OFF—pulse off-time, OV—voltage of open circuit).

From Figure 17a, the optimum condition for 2 mm rectangular fin length in the order of their ranking is WT2 OV3 ON3 WA3 WF3 OFF3. For this, wire tension (WT) emerges as the most critical factor, with the setting WT2, 700 g providing the optimal balance between wire stability and flexibility. This tension likely helps maintain consistent cutting performance along the entire length of the fin. The lowest voltage (OV3, 68.947 V) and shortest pulse on-time (ON3, 100 ns) suggest a more controlled, lower-energy cutting process. This combination may help reduce thermal effects and minimize dimensional changes along the fin length, which may eventually affect the fin width. The highest settings for flushing pressure (WA3, 8), feed rate (WF3, 14.105 m/min), and pulse off-time (OFF3, 20 μs) indicate a strategy that prioritizes efficient debris removal and cooling. This approach could help maintain consistent cutting conditions throughout the length of the fin. The predicted optimum response is −0.030%, which is about a 95% improvement, indicating excellent accuracy. This is expected, as the rectangular fin length largely performed well, having low deviations.

From Figure 17b, the optimum condition for 1 mm rectangular fin length in the order of their ranking is WT2 WF3 OFF2 ON3 WA3 OV2. For this, wire tension remains the most critical factor, again at the setting WT2, 700 g. This consistency suggests that this tension level provides a good balance for different fin lengths. The highest feed rate (WF3, 14.105 m/min) becomes the second-most important factor, possibly helping to maintain a consistent cutting speed along the fin length. The pulse off-time (OFF2, 14 μs) balances cooling and cutting efficiency. The shortest pulse on-time (ON3, 100 ns) and highest flushing pressure (WA3, 8) continue to prioritize precise cutting and efficient debris removal. The moderate oltage (OV2, 78.421 V) provides sufficient energy for cutting without excessive thermal effects. The predicted optimum response is 0.416%, which is about a 55% improvement.

From Figure 17c, the optimum condition for 0.5 mm rectangular fin length in the order of their ranking is OV1 WF3 WT2 ON3 OFF1 WA3. For this, we see a significant shift in the importance of factors. Voltage becomes the most critical factor, with the highest setting (OV1, 92.632 V) being optimal. This high voltage may be necessary to maintain a stable cutting process for such a small feature. The highest feed rate (WF3, 14.105 m/min) remains important, possibly to minimize the time the wire spends cutting this small feature. The consistent medium wire tension (WT2, 700 g) continues to provide a good balance. The shortest pulse on-time (ON3, 100 ns) and shortest pulse off-time (OFF1, 12 μs) suggest a strategy of very short, frequent pulses. This approach could help maintain precise control over the cutting process for this small feature. The highest flushing pressure (WA3, 8) ensures efficient debris removal, which is crucial for maintaining constant conditions in such a small cut. The predicted optimum response is −57.875% which is notably large. It must be noted that this high value is influenced by the high error value introduced due to the missing response variable for T7 for the 0.5 mm rectangular fin size.

#### 3.2.3. Optimum Conditions, Triangular Fin Length

Triangular fin length measurements demonstrate the consistent importance of pulse on-time, regardless of fin size. For small triangular fins, voltage of open circuit follows closely in significance, while for larger fins, feed rate becomes the second-most influential factor. Feed rate was consistently among the top three most influential factors for both sizes. Tables S27 and S28 in the Supplementary Materials show the response tables for each triangular fin length.

**Figure 18:**
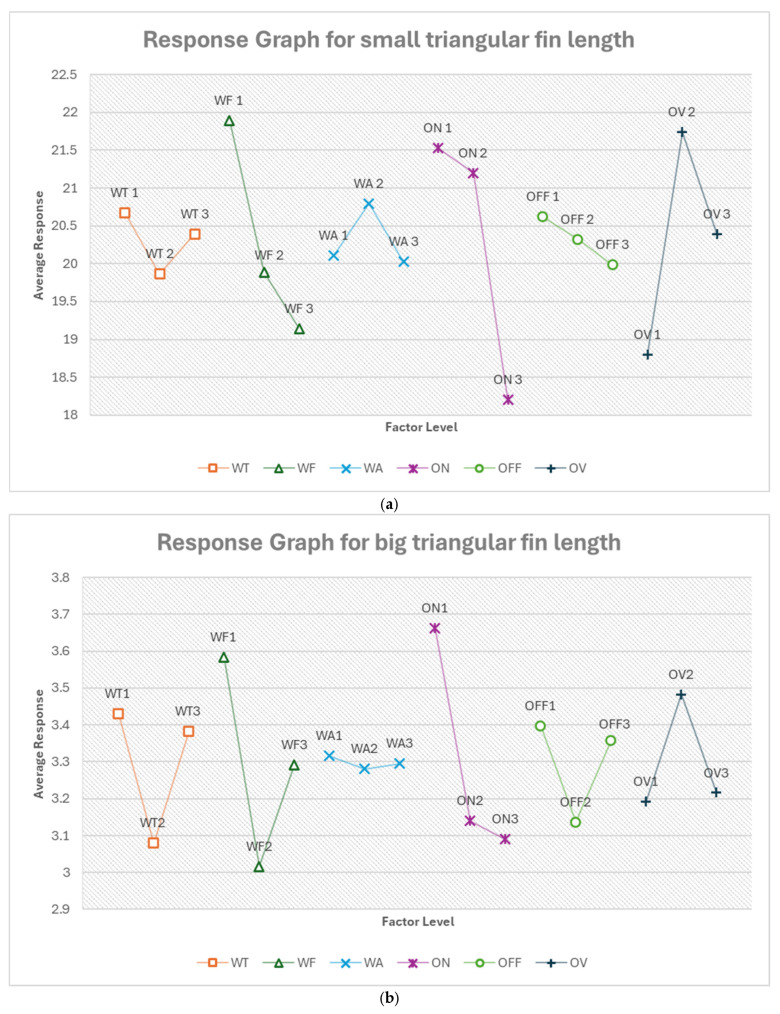
(**a**) Response graph for small triangular fin length, (**b**) response graph for big triangular fin length (WT—wire tension, WF—feed rate, WA—flushing pressure, ON—pulse on-time, OFF—pulse off-time, OV—voltage of open circuit).

From Figure 18a, the optimum condition for small triangular fin length in the order of their ranking is ON3 OV1 WF3 WT2 WA3 OFF3. For this, pulse on-time (ON) emerges as the most critical factor, with the shortest setting (ON3, 100 ns) being optimal. This short pulse duration likely allows for very precise control over the cutting process, which is crucial for the intricate geometry of a small triangular fin. The highest voltage (OV1, 92.632 V) is the second-most important factor. This high voltage may provide the necessary energy to maintain a stable cutting process for this complex shape, ensuring clean and accurate cuts along the triangular edges. The highest feed rate (WF3, 14.105 m/min) suggests that a faster cutting speed helps maintain consistency along the fin length, possibly reducing the risk of overheating or excessive erosion at any point. The medium wire tension (WT2, 700 g) continues to provide a good balance between wire stability and flexibility. The highest flushing pressure (WA3, 8) and longest pulse off-time (OFF3, 20 μs) indicate a strategy that prioritizes efficient debris removal and cooling. This is particularly important for the small triangular fin, where maintaining clean cutting conditions in tight spaces is crucial. The predicted optimum response is 14.473%, which is about a 29% improvement.

From Figure 18b, the optimum condition for big triangular fin length in the order of their ranking is ON3 WF2 WT2 OV1 OFF2 WA2. For this, pulse on-time (ON) remains the most critical factor, again with the shortest setting (ON3, 100 ns) being optimal. This consistency across both triangular fin sizes underscores the importance of precise, short pulses for accurately cutting angular geometries. The feed rate becomes the second-most important factor, with the medium setting (WF2, 10.421 m/min) being optimal. This moderate speed likely provides a good balance between cutting efficiency and maintaining accuracy over the longer length of the big triangular fin. The medium wire tension (WT2, 700 g) continues to be important, providing the right balance for cutting the longer edges of the big triangular fin accurately. The highest voltage (OV1, 92.632 V) remains optimal, ensuring sufficient energy for clean cuts along the entire length of the fin. The moderate pulse off-time (OFF2, 14 μs) and flushing pressure (WA2, 6) settings suggest that for the larger fin, a more balanced approach to cooling and debris removal is effective. The predicted optimum response is 2.311%, which is about a 30% improvement.

#### 3.2.4. Optimum Conditions, Internal and External Angles

The analysis of internal and external angles revealed the influence of factors varied significantly between different angles and cut types. Wire tension was consistently among the top three most influential factors for most angles and cut types, except for the 11.693° internal angle in straight cut and 53.753° internal angle in taper cut. Voltage of open circuit had a varying impact, being most influential for 11.693° internal angle in straight cut but less influential in other cases. Flushing pressure showed high influence for the 53.753° internal angle in taper cut but was generally less influential in other cases. Pulse on-time was consistently important across most angles and cut types. The optimal levels for each factor often changed between different angles and cut types, indicating that a one-size-fits-all approach is not suitable, just as observed in previous analysis. Tables S29–S34 in the Supplementary Materials show the response tables for each internal and external angel and cut type.

**Figure 19:**
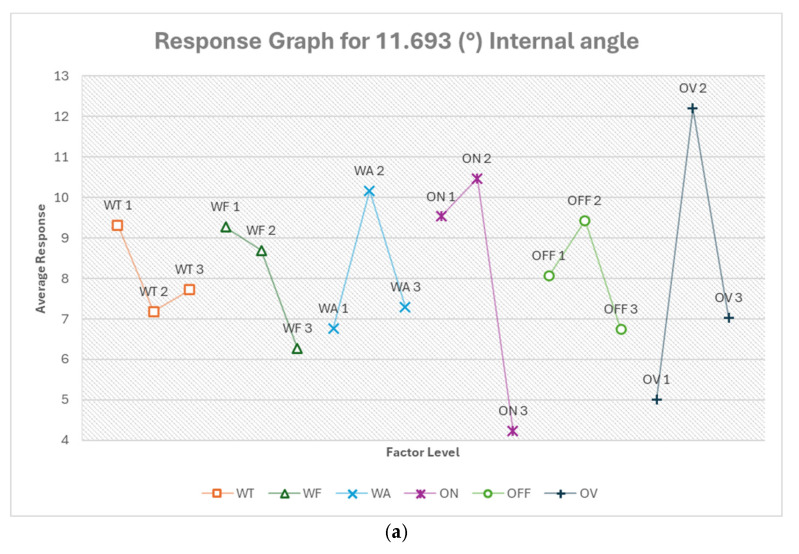

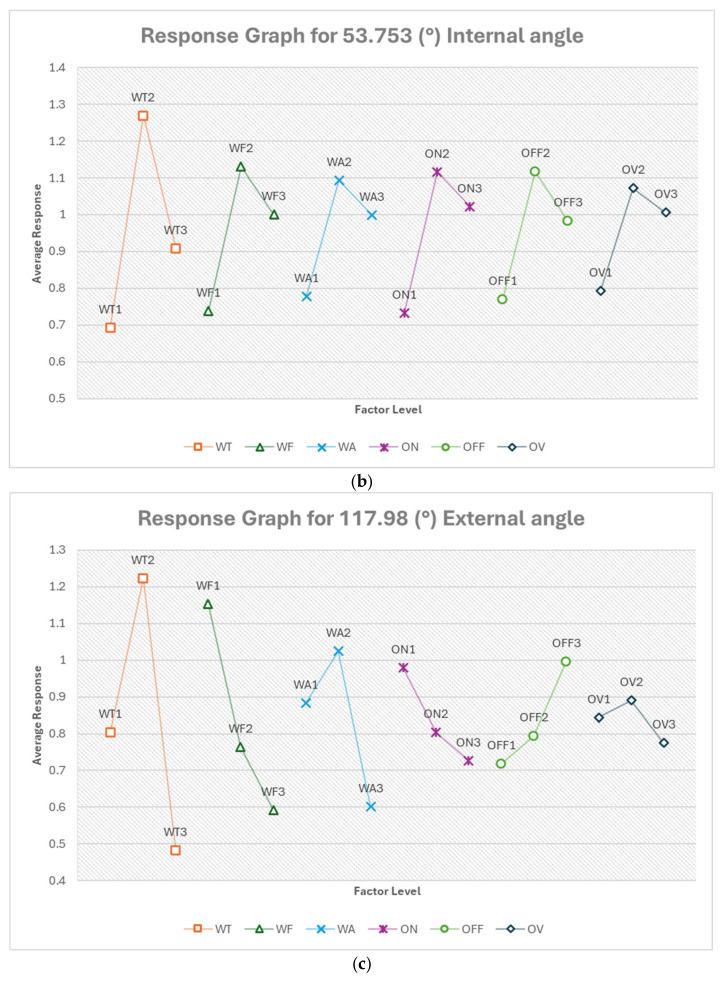
(**a**) Response graph for 11.693 (°) internal angle, (**b**) response graph for 53.753 (°) internal angle, (**c**) response graph for 117.98 (°) external angle (WT—wire tension, WF—feed rate, WA—flushing pressure, ON—pulse on-time, OFF—pulse off-time, OV—voltage of open circuit).

From Figure 19a, the optimum condition for 11.693° internal angle in the order of their ranking is OV1 ON3 WA1 WF3 OFF3 WT2. For this acute internal angle, voltage (OV) is the most critical factor, with the highest setting (OV1, 92.632 V) being optimal. This high voltage likely provides the energy needed for precise cutting in this tight angle. The shortest pulse on-time (ON3, 100 ns) allows for very controlled material removal, crucial for maintaining accuracy in such a small angle. The lowest flushing pressure (WA1, 4) may help prevent excessive erosion or deflection in this delicate area. The highest feed rate (WF3, 14.105 m/min) and longest pulse off-time (OFF3, 20 μs) suggest a strategy of quick passes with ample cooling time. The medium wire tension (WT2, 700 g) provides a balance between wire stability and flexibility. The predicted optimum response is −4.197%, which is about a 48% improvement.

From Figure 19b, the optimum condition for 53.753° internal angle in the order of their ranking is WT1 WF1 ON1 OFF1 WA1 OV1. For this wider internal angle, wire tension becomes the most critical factor, with the lowest setting (WT1, 500 g) being optimal. This lower tension may allow for better conformity to the angle. The slowest feed rate (WF1, 6.737 m/min), longest pulse on-time (ON1, 350 ns), and shortest pulse off-time (OFF1, 12 μs) suggest a slower, more continuous cutting process. The low flushing pressure (WA1, 4) and high voltage (OV1, 92.632 V) complete this gentle cutting approach. The predicted optimum response is −0.281%, which is about a 71% improvement.

From Figure 19c, the optimum condition for 117.98° external angle in the order of their ranking is WT3 WF3 WA3 OFF1 ON3 OV3. For this obtuse external angle, wire tension is again the most critical factor, but now at its highest setting (WT3, 800 g). This high tension likely helps maintain a straight, accurate cut for the external angle. The highest feed rate (WF3, 14.105 m/min) and flushing pressure (WA3, 8) suggest a more aggressive cutting approach, suitable for this less-constrained external angle. The shortest pulse off-time (OFF1, 12 μs) and pulse on-time (ON3, 100 ns) indicate a strategy of frequent, short pulses. The lowest voltage (OV3, 68.947 V) may help control thermal effects. The predicted optimum response is −0.284%, which is about a 66% improvement.

**Figure 20:**
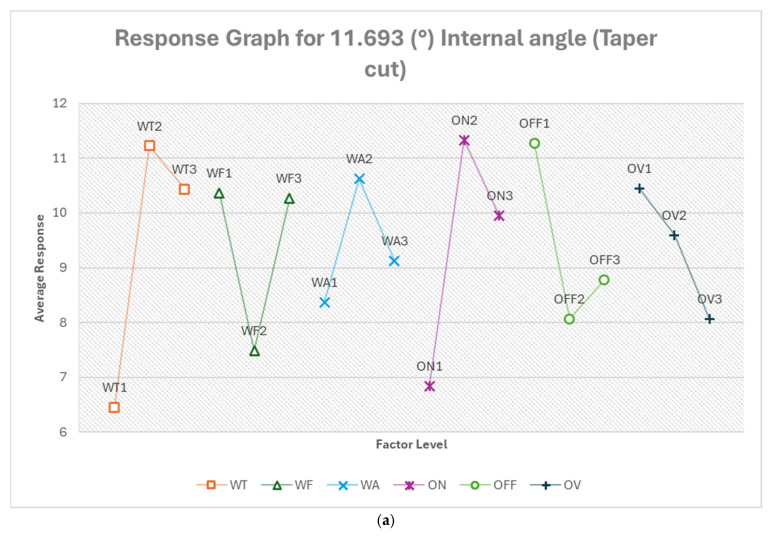

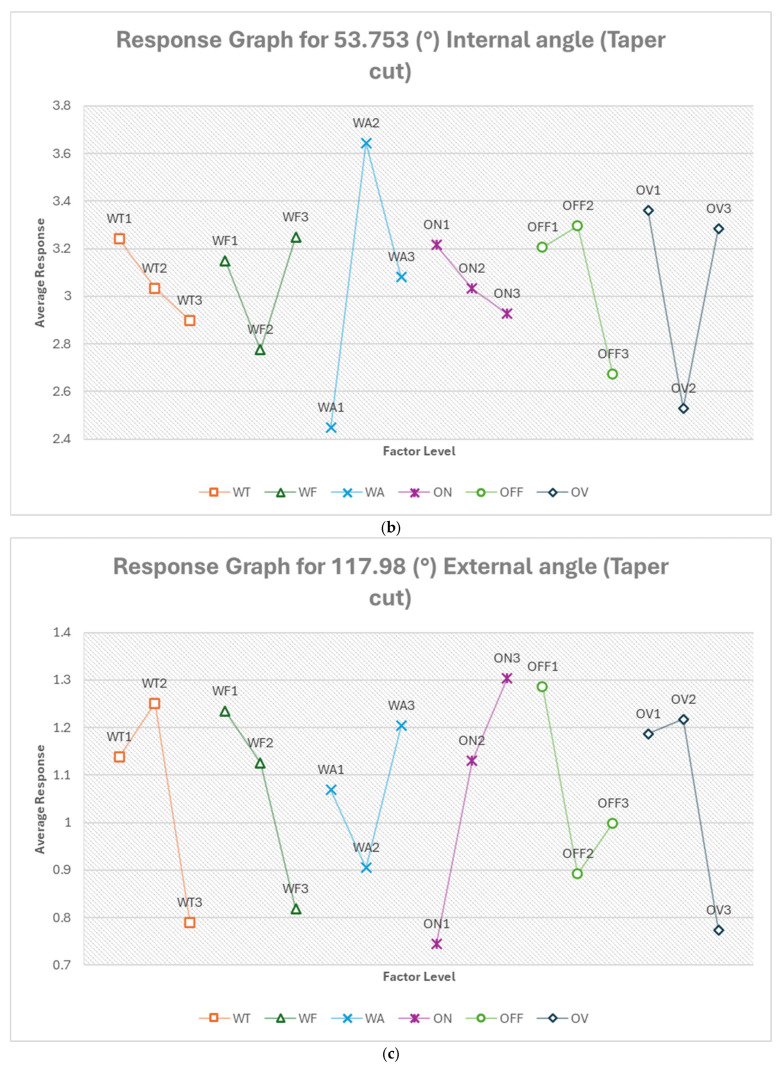
(**a**) Response graph for 11.693 (°) internal angle (Taper cut). (**b**) Response graph for 53.753 (°) internal angle (Taper cut). (**c**) Response graph for 117.98 (°) external angle (Taper cut). (WT—wire tension, WF—feed rate, WA—flushing pressure, ON—pulse on-time, OFF—pulse off-time, OV—voltage of open circuit).

From Figure 20a, the optimum condition for 11.693° internal angle (taper cut) in the order of their ranking is WT1 ON1 OFF2 WF2 OV3 WA1. For this acute internal angle in a taper cut, wire tension becomes the most critical factor, with the lowest setting (WT1, 500 g) being optimal. This low tension may allow the wire to better conform to the taper and tight angle. The longest pulse on-time (ON1, 350 ns) and moderate pulse off-time (OFF2, 14 μs) suggest a more continuous cutting process. The moderate feed rate (WF2, 10.421 m/min) balances speed and precision. The lowest voltage (OV3, 68.947 V) and flushing pressure (WA1, 4) indicate a gentler cutting approach, suitable for this challenging geometry. The predicted optimum response of −1.575%, which is about an 83% improvement.

From Figure 20b, the optimum condition for 53.753° internal angle (taper cut) in the order of their ranking is WA1 OV2 OFF3 WF2 WT3 ON3. For this wider internal angle in a taper cut, flushing pressure becomes the most critical factor, with the lowest setting (WA1, 4) being optimal. This low pressure may help prevent distortion in the tapered cut. The moderate voltage (OV2, 78.421 V) and longest pulse off-time (OFF3, 20 μs) suggest a balanced approach to energy input and cooling. The moderate feed rate (WF2, 10.421 m/min) maintains consistent cutting speed. The highest wire tension (WT3, 800 g) may help maintain wire stability in the tapered cut. The shortest pulse on-time (ON3, 100 ns) allows for precise control. The predicted optimum response is 0.962%, which is about a 69% improvement.

From Figure 20c, the optimum condition for 117.98° external angle (taper cut) in the order of their ranking is ON1 WT3 OV3 WF3 OFF2 WA2. For this obtuse external angle in a taper cut, pulse on-time becomes the most critical factor, with the longest setting (ON1, 350 ns) being optimal. This longer pulse may provide more consistent material removal along the taper. The highest wire tension (WT3, 800 g) helps maintain wire stability for this external angle. The lowest voltage (OV3, 68.947 V) may help control thermal effects in the taper cut. The highest feed rate (WF3, 14.105 m/min), moderate pulse off-time (OFF2, 14 μs), and moderate flushing pressure (WA2, 6) suggest a balanced approach to cutting speed, cooling, and debris removal. The predicted optimum response is −0.374%, which is about a 65% improvement.

#### 3.2.5. Optimum Conditions, Gear Profile

The analysis of gear profile response variables revealed intricate relationships between machining parameters and gear characteristics. For eight-teeth gears, pulse off-time and pulse on-time were the most influential factors for addendum deviation, while wire tension and feed rate dominated for dedendum deviation. Circular width deviation was most affected by flushing pressure and voltage of open circuit. In contrast, four-teeth gears showed wire tension as the primary influential factor for both addendum and dedendum deviations, with pulse off-time being most critical for circular width deviation. Notably, the optimal levels for each factor often differ between gear functional parts and sizes, underscoring the complexity of the manufacturing process. Wire tension and pulse off-time consistently ranked among the top influential factors across most scenarios, suggesting their critical role in gear profile optimization. However, the varying impact of factors like flushing pressure and voltage of open circuit across different gear functional parts and sizes indicates that a one-size-fits-all approach is unsuitable. These findings emphasize the need for dynamic process adjustments and careful parameter optimization based on specific gear sizes and characteristics to achieve optimal results in gear profile manufacturing. Tables S35–S40 in the Supplementary Materials show the response tables for each gear functional part for both eight- and four-teeth gear profiles.

**Figure 21:**
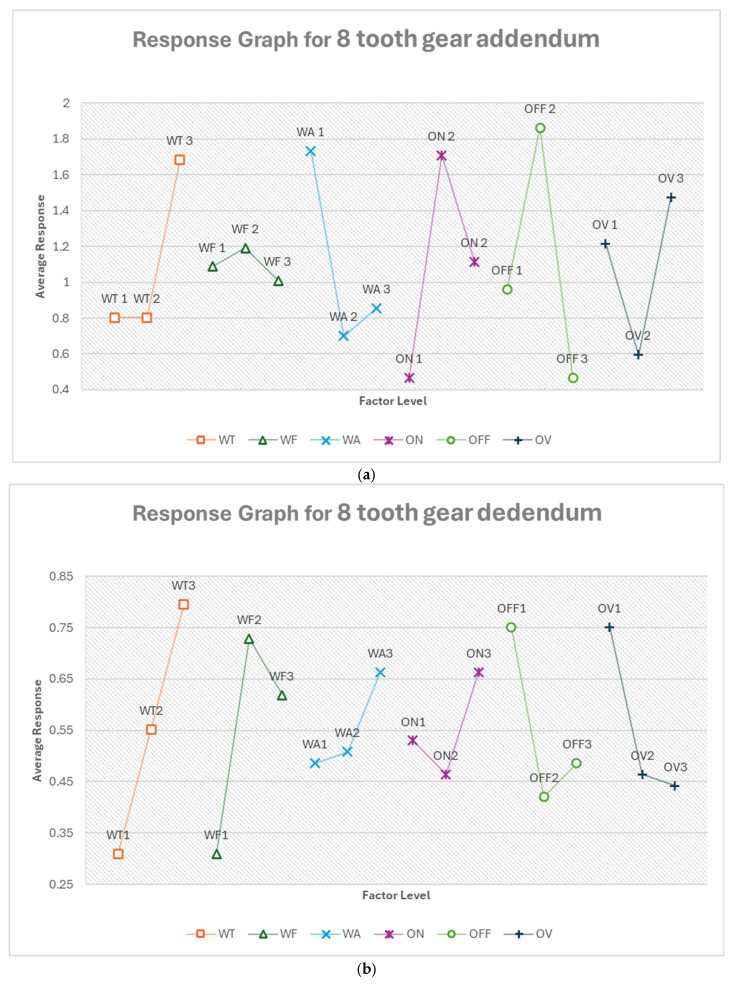

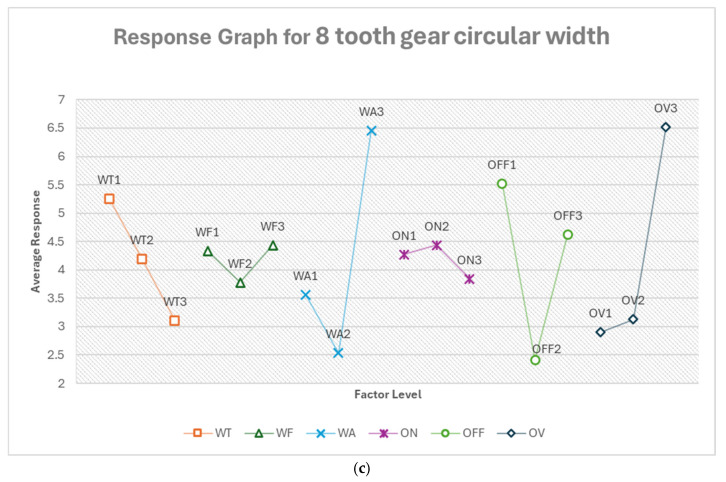
(**a**) Response graph for 8-teeth gear addendum. (**b**) Response graph for 8-teeth gear dedendum. (**c**) Response graph for 8-teeth gear circular width. (WT—wire tension, WF—feed rate, WA—flushing pressure, ON—pulse on-time, OFF—pulse off-time, OV—voltage of open circuit).

From Figure 21a, the optimum condition for eight-teeth gear addendum in the order of their ranking is OFF3 ON1 WA2 WT1 OV2 WF3. For the addendum of the eight-teeth gear, pulse off-time (OFF) is the most critical factor, with the longest setting (OFF3, 20 μs) being optimal. This extended off-time may help in achieving better surface finish and dimensional accuracy at the gear tips. The longest pulse on-time (ON1, 350 ns) suggests that longer energy pulses are beneficial for cutting the addendum profile. The moderate flushing pressure (WA2, 6) balances debris removal with minimal disturbance to the cut. The lowest wire tension (WT1, 500 g) may allow for better conformity to the curved addendum profile. The moderate voltage (OV2, 78.421 V) and highest feed rate (WF3, 14.105 m/min) complete the optimum condition. It must be noted that wire tension and voltage of open circuit effects were tied. Moreso, the factor level effects of WTI and WT2 remained the same. (Thus, their average responses were tied as well, as shown in Figure 21a). This means using WT2, 700 g in the optimum condition would yield the same effect.

The predicted optimum response is −1.441%. This prediction is a bit alarming, as it seems to be higher than the mean (1.096) of the measured responses. Typically, this is a sign of wrong calculations or data handling error, but after repeated calculations and cross-checking, the data being analyzed multiple times, it remains the same. The next was to check for extreme factor effects. Since there was none, it was determined that this could be the result of the limitation of the response graph method. It treats each parameter independently, without accounting for complex interactions between factors. As a result, when a combination of optimal levels is predicted, the response may not always fall within the measured range, especially if interaction effects or nonlinearities are present.

Despite this limitation, the model remains a valuable tool for parameter ranking and trend analysis. It effectively identifies the most influential factors and offers reliable guidance for process tuning, especially in multi-parameter environments. For future work, we plan to explore other models such as the grey relational analysis.

From Figure 21b, the optimum condition for eight-teeth gear dedendum in the order of their ranking is WT1 WF1 OFF2 OV3 ON2 WA1. For this, wire tension becomes the most critical factor, with the lowest setting (WT1, 500 g) being optimal. The slowest feed rate (WF1, 6.737 m/min) suggests a more careful approach is needed for the dedendum. The moderate pulse off-time (OFF2, 14 μs), lowest voltage (OV3, 68.947 V), and moderate pulse on-time (ON2, 225 ns) indicate a balanced cutting strategy. The lowest flushing pressure (WA1, 4) may help prevent erosion in the dedendum area. The predicted optimum response is −0.332%, which is about a 40% improvement.

From Figure 21c, the optimum condition for eight-teeth gear circular width in the order of their ranking is WA2 OV1 OFF2 WT3 WF2 ON3. For this, flushing pressure becomes the most critical factor, with the moderate setting (WA2, 6) being optimal. This may help in maintaining consistent cutting conditions along the tooth width. The highest voltage (OV1, 92.632 V) suggests that more energy is beneficial for achieving the correct width. The moderate pulse off-time (OFF2, 14 μs) balances cooling and cutting efficiency. The highest wire tension (WT3, 800 g) may help in maintaining a straight cut for the width. The moderate feed rate (WF2, 10.421 m/min) and shortest pulse on-time (ON3, 100 ns) complete the strategy. The predicted optimum response is −2.337%, which is about a 44% improvement.

**Figure 22:**
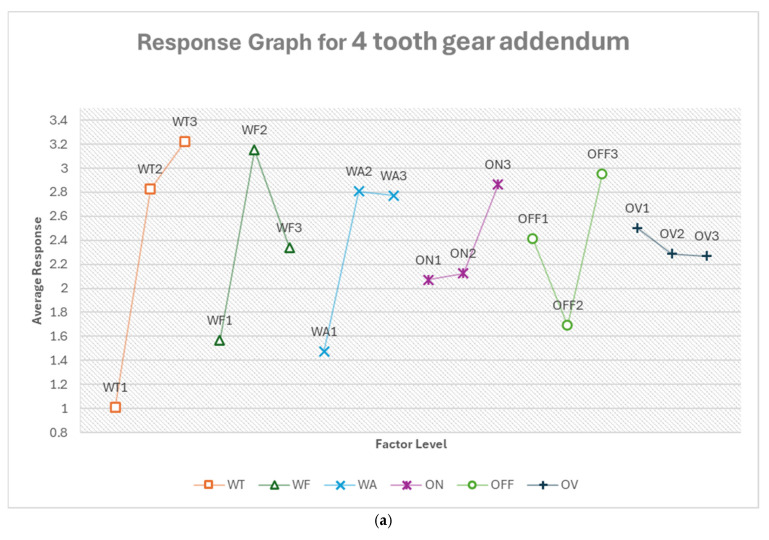

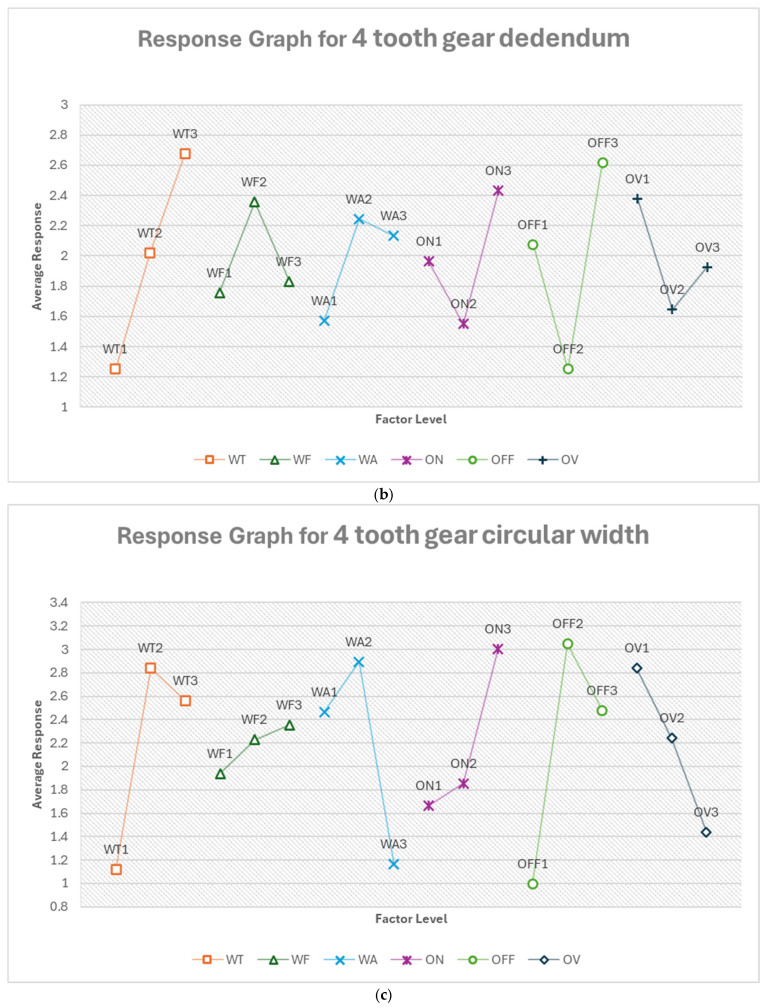
(**a**) Response graph for 4-teeth gear addendum. (**b**) Response graph for 4-teeth gear dedendum. (**c**) Response graph for 4-teeth gear circular width. (WT—wire tension, WF—feed rate, WA—flushing pressure, ON—pulse on-time, OFF—pulse off-time, OV—voltage of open circuit).

From Figure 22a, the optimum condition for four-teeth gear addendum in the order of their ranking is WT1 WF1 WA1 OFF2 ON1 OV3. For this, wire tension becomes the most critical factor, with the lowest setting (WT1, 500 g) being optimal. This low tension may allow for better conformity to the more curvature of the larger teeth. The slowest feed rate (WF1, 6.737 m/min) and lowest flushing pressure (WA1, 4) suggest a gentler approach is needed for these larger teeth. The moderate pulse off-time (OFF2, 14 μs), longest pulse on-time (ON1, 350 ns), and lowest voltage (OV3, 68.947 V) indicate a balanced, lower-energy cutting strategy. The predicted optimum response is −1.681%, which is about a 29% improvement.

From Figure 22b, the optimum condition for four-teeth gear dedendum in the order of their ranking is WT1 OFF2 ON2 OV2 WA1 WF2. For this, wire tension remains the most critical factor, again with the lowest setting (WT1, 500 g) being optimal. This consistency with the addendum suggests that low tension is generally beneficial for the larger tooth profiles. The moderate settings for pulse off-time (OFF2, 14 μs), pulse on-time (ON2, 300 ns), and voltage (OV2, 78.421 V) indicate a balanced cutting approach. The lowest flushing pressure (WA1, 4) may help prevent erosion in the dedendum area. The moderate feed rate (WF2, 10.421 m/min) balances cutting speed and precision. The predicted optimum response is −0.281%, which is about an 86% improvement.

From Figure 22c, the optimum condition for four-teeth gear circular width in the order of their ranking is OFF1 WA3 WT1 OV3 ON1 WF1. For this, pulse off-time becomes the most critical factor, with the shortest setting (OFF1, 12 μs) being optimal. This short off time may help in achieving a more consistent cut along the wider tooth. The highest flushing pressure (WA3, 8) suggests that efficient debris removal is crucial for maintaining accuracy across the wider tooth. The lowest wire tension (WT1, 500 g) may allow for better conformity to any slight curvature in the width. The lowest voltage (OV3, 68.947 V), longest pulse on-time (ON1, 350 ns), and slowest feed rate (WF1, 6.737 m/min) indicate a careful, lower-energy cutting approach. The predicted optimum response is −2.549%. Just like in the case of the eight-teeth gear addendum, this prediction shows a response that is higher than the mean value (2.174) of the measured data. This was equally attributed to the limitation of the method used. Again, as discussed earlier under the eight-teeth gear addendum of this section, the model remains reliable despite this limitation.

## 4. Effect of Workpiece Thickness

Workpiece thickness plays a critical role in determining machining accuracy in wire EDM, particularly for complex geometries involving taper cuts and sharp corners. To evaluate this effect, an auxiliary experiment was conducted using two thicknesses of 6061 aluminum alloy: 9.6 mm and 6.22 mm, under identical WEDM parameters listed in Table S41. Figure S3–S5 show the machined profiles in two different thicknesses. Tables S42–S55 present all the measurement data demonstrating effects of workpiece thicknesses on profile accuracies.

### 4.1. Rectangular Fins

Deviations in fin length were more pronounced in the 9.6 mm workpiece (up to −1.7 mm) than in the 6.22 mm (as low as −0.3 mm), suggesting that thinner materials facilitate more stable cutting, likely due to reduced wire deflection and vibration. Taper angles also deviated more in the thicker specimen, with thinner workpieces achieving values closer to the intended 5°.

### 4.2. Triangular Fins

Side lengths and internal angles of triangular fins showed higher deviations in thicker workpieces, with deformations and measurement difficulties occurring more frequently. This aligns with reports in literature attributing such deviations to amplified wire lag and thermal stress in thicker materials.

### 4.3. Gear Profiles

In contrast, gear features such as addendum, dedendum, and tooth width showed minimal deviations across both thicknesses, suggesting that certain symmetrical and robust geometries are less sensitive to thickness variations under consistent parameter control.

### 4.4. Impications

The findings reinforce that for high-precision cutting of micro-features or complex geometries, thinner workpieces are preferable. However, trade-offs with stiffness, heat dissipation, and part strength must be evaluated. The outcomes provide valuable guidance for material selection and fixture strategies. As this auxiliary experiment was performed earlier, this insight directly informed our decision to use the 9.6 mm thickness in the main study of this manuscript. The premise was to evaluate WEDM parameter optimization under challenging conditions where dimensional deviations are more likely. This approach would then allow us to identify robust process windows that could later be translated to less challenging conditions (e.g., thinner workpieces) with potentially even better outcomes.

## 5. Conclusions

This study investigated the accuracy of corners in machined complex geometrical parts and taper cuts using the L18 Taguchi orthogonal array design of experiments. The research aimed to determine the optimum cutting conditions/parameters that would produce better accuracy in the measured parts of complex geometry. Based on the comprehensive analysis presented in this study, several key conclusions can be drawn:

### 5.1. Parameter Optimization

The study successfully identified optimum cutting parameters for various feature sizes and geometries, including rectangular fins, triangular fins, internal and external angles, and gear profiles. These optimized parameters demonstrated significant improvements in minimizing percent deviations from target dimensions. Nonetheless, some of the optimum conditions, like the case of the eight-teeth gear addendum and four-teeth gear circular width, were concerning, because their predicted optimum response tended to be higher than the overall mean response of the measured data. This highlighted the limitation of the optimization technique used. Table 11 shows the compiled list of all 22 optimum conditions for minimizing the deviations in the measured data for all measurements of interest.

### 5.2. Taper Cut Complexity

Taper cuts introduced additional complexity to the machining process. The study revealed that different optimum conditions were required for taper cuts compared to straight cuts, even for the same nominal dimensions. This emphasizes the importance of tailoring cutting strategies to specific geometries. Table 11 shows that the optimum settings of parameters change when machining the same features with taper cutting.

### 5.3. Critical Parameters

While the importance of different parameters varied across features, some factors consistently emerged as critical. Wire tension (WT) was frequently among the most influential factors (top three ranking), highlighting its importance in maintaining cutting accuracy. This was followed by pulse on-time (ON). For the individual measurements of interest, pulse on-time (ON), feed rate (WF) and voltage of open circuit (OV) placed equally in the top three ranking of the rectangular fin width (straight cut). For taper cut, wire tension (WT) was the critical parameter. For rectangular fin length, wire tension (WT), and feed rate (WF), and voltage of open circuit (OV) were critical. For triangular fin length, pulse on-time (ON) and feed rate (WF) were critical. For internal and external angles, wire tension (WT) and pulse on-time (ON) were critical for both straight and taper cut. For gears, pulse off-time (OFF) was the critical parameter. Nonetheless, wire tension (WT) and flashing pressure (WA) equally showed a strong presence. Although these parameters are critical, their optimal levels often varied depending on the specific feature being cut, emphasizing the need for feature-specific optimization. Cooling and debris removal are important in the machining of gears and for parts with internal and external angles, as flashing pressure (WA) and pulse off-time (OFF) show strong presence.

### 5.4. Effects of Critical Parameters on Accuracy

Wire tension (WT) and pulse on-time (ON) have been found to be the most critical parameters for the optimization process, each appearing 15 and 12 times, respectively, in the top three ranks out of the 22 optimum conditions. As mentioned earlier, wire tension affects the straightness and stability of the wire, influencing cutting accuracy and surface finish. As such, how tight or loose the wire is directly affects the amplitude of the wire vibration. As already established, wire vibration is a major source of dimensional accuracy. This explains the dominance of wire tension. Moreover, given the complex nature of the geometrical features, it is imperative that the straightness and stability of the wire is accounted for to prevent large vibrational amplitudes and even wire breakage. Similarly, pulse on-time (ON) has been defined to determine the duration of each spark. Whilst long spark on a single area may affect surface roughness, wear the wire, and cause a large heat-affected zone, it is necessary for high material removal. As such, although long ON may be preferred, short ON takes dominance for extremely low and critical dimensions.

### 5.5. Physical Significance and Technological Recommendation

In conclusion, the findings from this study have practical relevance for manufacturers seeking to machine complex profiles with high accuracy using WEDM. Based on the parameter optimization results, it is recommended that manufacturers prioritize wire tension and pulse timing settings when cutting small, detailed features or tapered geometries. Specifically, lower wire tension may be more suitable for curved profiles to reduce tool deflection, while longer pulse on-times benefit material removal but require balancing with pulse off-times for stability.

For applications involving thicker workpieces, operators should expect higher wire vibration and may need to increase tension and flushing pressure to maintain precision. Designers are encouraged to account for these dependencies during material selection and profile design to reduce rework and post-processing. These insights can help both OEMs and subcontractors improve yield, reduce part rejection rates, and streamline WEDM process planning for high-precision applications in the aerospace, tooling, and biomedical sectors.

While this study focused on investigating corner and profile accuracies in a simple aluminum 6061 alloy without emphasis on workpiece, future studies could focus on optimizing parameters for some of the important materials that are being cut by WEDM process in industries, such as tungsten carbide [44], silicon [45], bulk metallic glass [46], steel [47], or even fiber-reinforced polymer composites [48]. Moreover, this study kept the wire electrode unchanged; therefore, future studies can focus on using coated electrode wire, such as zinc-coated brass wire electrode [49], to investigate the effects of electrode wire on profile accuracy. In addition, the corner and profile accuracies may also depend on the selection of EDM pulse generators, as resistor–capacitor (RC)-type pulse generators were found to supply comparatively lower discharge energy per pulse during machining [50].

Whilst this study focused on dimensional accuracy, the optimum condition that is efficient in accuracy may be deficient in surface roughness, energy efficiency, tool wear, and overall cut time. As such, future work could consider multiple objectives optimization that would simultaneously provide the optimum solution that will balance these other objectives. The grey relational analysis or desirability function approach may be employed for such purposes. The multi-objective optimization approach allows for the visualization of trade-offs between different objectives (e.g., accuracy vs. surface roughness), providing flexibility in prioritizing different objectives based on specific application requirements.

Although the primary objective of this study was to optimize dimensional accuracy in WEDM for complex geometries, it is important to consider the environmental implications of the process. WEDM consumes significant energy and materials, particularly through wire electrode usage and dielectric fluid consumption. Future work could explore sustainable machining strategies, such as reducing wire waste through optimized cutting paths, implementing dielectric fluid filtration and recycling systems, and using energy-efficient pulse parameter settings to minimize power consumption per cut. These strategies not only support environmental goals but also reduce operational costs, aligning WEDM process optimization with emerging sustainability standards in advanced manufacturing.

## Figures and Tables

**Table 1:** Cutting condition of Excetek wire-EDM.

Cutting Condition	Description
Cutting condition number—E PARK NO (NO)	-
Cutting mode, fine finish power selection (PM)	0~10
Voltage of open circuit (OV)	1~20; (voltage from 50–140 V)
On-time (ON)	1~24; (50–1200 ns)
Off-time (OFF)	4~50; (4–50 µs)
Arc on-time (AON)	1~16; (50–800 ns)
Arc off-time (AOFF)	4~50; (3–50 µs)
Servo voltage (SV)	16~75 V
Wire tension (WT)	1~20; (300–2220 g)
Wire feed (WF)	1~20; (6–20 m/min)
Water flow (WA)	1~8
Feedrate override (FR%)	1~400%
Feedrate (F)	0.1~500 mm^2^/min
M90/M91 (SM)	M90 (G95) auto servo feed cutting mode
Servo sensitivity (SC)	1~99

**Table 2:** Factors and levels description for Taguchi orthogonal array.

Factors	Level 1	Level 2	Level 3
Cut type	Straight	5° taper cut	N/A
Wire tension (WT)	3 (500 g)	5 (700 g)	6 (800 g)
Feed rate (WF)	2 (6.737 m/min)	7 (10.421 m/min)	12 (14.105 m/min)
Flushing pressure (WA)	4	6	8
Pulse on-time (ON)	7 (350 ns)	6 (300 ns)	2 (100 ns)
Pulse off-time (OFF)	12 us	14 us	20 us
Voltage of open circuit (OV)	10 (92.632 V)	7 (78.421 V)	5 (68.947 V)

**Table 3:** L18 Taguchi orthogonal array showing combination of parameters for each experiment.

Experiment No.	Cut Type	Wire Tension (WT)	Feed Rate (WF)	Flushing Pressure (WA)	Pulse On-Time (ON)	Pulse Off-Time (OFF) Us	Voltage of Open Circuit (OV)
1	Straight	3 (500 g)	2 (6.737 m/min)	4	7 (350 ns)	12	10 (92.632 V)
2	Straight	3 (500 g)	7 (10.421 m/min)	6	6 (300 ns)	14	7 (78.421 V)
3	Straight	3 (500 g)	12 (14.105 m/min)	8	2 (100 ns)	20	5 (68.947 V)
4	Straight	5 (700 g)	2 (6.737 m/min)	4	6 (300 ns)	14	5 (68.947 V)
5	Straight	5 (700 g)	7 (10.421 m/min)	6	2 (100 ns)	20	10 (92.632 V)
6	Straight	5 (700 g)	12 (14.105 m/min)	8	7 (350 ns)	12	7 (78.421 V)
7	Straight	6 (800 g)	2 (6.737 m/min)	6	7 (350 ns)	20	7 (78.421 V)
8	Straight	6 (800 g)	7 (10.421 m/min)	8	6 (300 ns)	12	5 (68.947 V)
9	Straight	6 (800 g)	12 (14.105 m/min)	4	2 (100 ns)	14	10 (92.632 V)
10	5° taper cut	3 (500 g)	2 (6.737 m/min)	8	2 (100 ns)	14	7 (78.421 V)
11	5° taper cut	3 (500 g)	7 (10.421 m/min)	4	7 (350 ns)	20	5 (68.947 V)
12	5° taper cut	3 (500 g)	12 (14.105 m/min)	6	6 (300 ns)	12	10 (92.632 V)
13	5° taper cut	5 (700 g)	2 (6.737 m/min)	6	2 (100 ns)	12	5 (68.947 V)
14	5° taper cut	5 (700 g)	7 (10.421 m/min)	8	7 (350 ns)	14	10 (92.632 V)
15	5° taper cut	5 (700 g)	12 (14.105 m/min)	4	6 (300 ns)	20	7 (78.421 V)
16	5° taper cut	6 (800 g)	2 (6.737 m/min)	8	6 (300 ns)	20	10 (92.632 V)
17	5° taper cut	6 (800 g)	7 (10.421 m/min)	4	2 (100 ns)	12	7 (78.421 V)
18	5° taper cut	6 (800 g)	12 (14.105 m/min)	6	7 (350 ns)	14	5 (68.947 V)

**Table 4:** Rectangular fin length and width target dimensions.

Fin Width (mm)	Fin Number	Fin Length (mm)
2.000	1	10.000
	2	10.000
	3	10.000
1.000	1	10.000
	2	10.000
	3	10.000
0.500	1	10.000
	2	0.500
	3	0.500

**Table 5:** Triangular fin length target dimensions.

Fin Side Length (mm)	Fin Number	Actual/Intended (mm)
Small triangular fin	1	9.888
	2	9.887
Big triangular fin	1	10.938
	2	11.030

**Table 6:** Internal and external angles for triangular fin part geometry target dimensions.

Internal Angle	External Angle
11.693°	117.98°
53.753°	

**Table 7:** Selected functional parts of the gear target dimensions.

	Addendum (mm)	Dedendum (mm)	Circular Thickness/Width (mm)
8 tooth half gear	1.288	1.508	1.631
4 tooth half gear	1.851	1.782	2.407

**Table 8:** T1 measurement and response.

(a) Measured Dimensions of Rectangular Fin Width				
Fin width (mm)	Fin Number	Actual/Intended (mm)	Measured (mm)	% Deviation
2	1	2.000	1.673	16.350
	2	2.000	1.677	16.150
	3	2.000	1.692	15.400
1	1	1.000	0.682	31.800
	2	1.000	0.698	30.200
	3	1.000	0.670	33.000
0.5	1	0.500	0.197	60.600
	2	0.500	0.197	60.600
	3	0.500	0.158	68.400
(b) Measured Dimensions of Rectangular Fin Length				
Fin length (mm)	Fin Number	Actual/Intended (mm)	Measured (mm)	% Deviation
2	1	10.000	9.893	1.070
	2	10.000	9.893	1.070
	3	10.000	9.893	1.070
1	1	10.000	9.893	1.070
	2	10.000	9.858	1.420
	3	10.000	9.858	1.420
0.5	1	10.000	9.792	2.080
	2	10.000	9.786	2.140
	3	10.000	9.791	2.090
(c) Measured Dimensions of Triangular Fins				
Fin side length (mm)	Fin Number	Actual/Intended (mm)	Measured (mm)	% Deviation
Small triangular fin	1	9.888	7.813	20.985
	2	9.887	7.798	21.129
Big triangular fin	1	10.938	10.511	3.904
	2	11.030	10.642	3.518
(d) Measured Internal and External Angles				
Angles (°)	Actual/Intended (°)	Measured (°)	% Deviation	
Internal—small triangular fin	11.693	12.560	7.415	
Internal—big triangular fin	53.753	53.819	0.123	
External	117.980	116.789	1.009	
(e) Measured Dimensions of 8-Teeth Gear Profile				
Profile type (mm)	Actual/Intended (mm)	Measured (mm)	% Deviation	
Addendum	1.288	1.278	0.776	
Dedendum	1.508	1.501	0.464	
Tooth thickness/width	1.631	1.542	5.457	
(f) Measured Dimensions of 4-Teeth Gear Profile				
Profile type (mm)	Actual/Intended (mm)	Measured (mm)	% Deviation	
Addendum	1.851	1.843	0.432	
Dedendum	1.782	1.755	1.515	
Tooth thickness/width	2.407	2.398	0.374	

**Table 9:** T10 measurement and response.

(a) Measured Dimensions of Rectangular Fins (5° Taper Cut)						
Fin Size (mm)	Fin Number	Measured Top Face (mm)	Measured Bottom Face (mm)	Calculated Taper Angle (rad)	Calculated Taper Angle (°)	% Deviation
2	1	2.815	1.203	0.084	4.800	3.996
	2	2.850	1.207	0.085	4.891	2.179
	3	2.854	1.205	0.086	4.909	1.823
1	1	1.706	0.229	0.077	4.399	12.021
	2	1.483	0.235	0.065	3.721	25.580
	3	1.713	0.240	0.077	4.385	12.298
0.5	1	Part defective. Measurement not taken	Part defective. Measurement not taken	NA	NA	NA
	2					
	3					
(b) Measured Internal and External Angles (5° Taper Cut)						
Angles (°)	Actual/Intended (°)	Measured (°)	% Deviation			
Internal—small triangular fin	11.695	10.951	6.362			
Internal—big triangular fin	54.734	53.069	3.042			
External	117.980	116.443	1.303			

**Table 10:** Response table for 2 mm rectangular fin width.

Straight Cut	Factors	Wire Tension (WT)	Feed Rate (WF)	Flushing Pressure (WA)	Pulse On-Time (ON)	Pulse Off-Time (OFF) Us	Voltage of Open Circuit (OV)
	Level 1	134.733	146.567	138.866	148.818	146.018	136.966
	Level 2	139.050	129.717	132.517	131.617	126.716	136.668
	Level 3	146.801	144.300	149.201	140.149	147.850	146.950
	Average Response						
	Factors	Wire Tension (WT)	Feed Rate (WF)	Flushing Pressure (WA)	Pulse On-Time (ON)	Pulse Off-Time (OFF) Us	Voltage of Open Circuit (OV)
	Level 1	14.970	16.285	15.430	16.535	16.224	15.218
	Level 2	15.450	14.413	14.724	14.624	14.080	15.185
	Level 3	16.311	16.033	16.578	15.572	16.428	16.328
	Difference	1.341	1.872	1.854	1.911	2.348	1.142
	Rank	5	3	4	2	1	6

**Table 11:** List of optimum conditions in order of their ranking for all measurements of interest.

Measurement of Interest	Optimum Condition	Respective Optimum Condition Values
Rectangular Fin Width		
2 mm fin width	OFF2 ON2 WF2 WA2 WT1 OV2	14 us, 300 ns, 10.421 m/min, 6, 500 g, 78.421 V
1 mm fin width	WT2 OV1 WA1 ON3 OFF2 WF2	700 g, 92.632 V, 4, 100 ns, 14 us, 10.421 m/min
0.5 mm fin width	OV1 ON3 WF2 OFF2 WT2 WA1	92.632 V, 100 ns, 10.421 m/min, 14 us, 700 g, 4

2 mm fin width (taper cut)	WA2 WT1 OFF1 OV3 WF3 ON2	6, 500 g, 12 us, 68.947 V, 14.405 m/min, 300 ns
1 mm fin width (taper cut)	WT1 ON2 OV2 WA1 OFF1 WF3	500 g, 300 ns, 78.421 V, 4, 12 us, 14.105 m/min
Rectangular Fin Length		
2 mm fin length	WT2 OV3 ON3 WA3 WF3 OFF3	700 g, 68.947 V, 100 ns, 8, 14.105 m/min, 20 us
1 mm fin length	WT2 WF3 OFF2 ON3 WA3 OV2	700 g, 14.105 m/min, 14 us, 100 ns, 8, 78.421 V
0.5 mm fin length	OV1 WF3 WT2 ON3 OFF1 WA3	92.632 V, 14.105 m/min, 700 g, 100 ns, 12 us, 8
Triangular Fin Length		
Small Triangular Fin	ON3 OV1 WF3 WT2 WA3 OFF3	100 ns, 92.632 V, 14.105 m/min, 700 g, 8, 20 us
Big Triangular Fin	ON3 WF2 WT2 OV1 OFF2 WA2	100 ns, 10.421 m/min, 700 g, 92.632 V, 14 us, 6
Internal and External Angles		
11.693 (°) internal angle	OV1 ON3 WA1 WF3 OFF3 WT2	92.632 V, 100 ns, 4, 14.105 m/min, 20 us, 700 g
53.753 (°) internal angle	WT1 WF1 ON1 OFF1 WA1 OV1	300 g, 6.737 m/min, 350 ns, 12 us, 4, 92.632 V
117.98 (°) external angle	WT3 WF3 WA3 OFF1 ON3 OV3	800 g, 14.105 m/min, 8, 12 us, 100 ns, 68.947 V
11.693 (°) internal angle	WT1 ON1 OFF2 WF2 OV3 WA1	500 g, 350 ns, 14 us, 10.421 m/min, 68.947 V, 4
53.753 (°) internal angle	WA1 OV2 OFF3 WF2 WT3 ON3	4, 78.421 V, 20us, 10.421 m/min, 800 g, 100 ns
117.98 (°) external angle	ON1 WT3 OV3 WF3 OFF2 WA2	350 ns, 800 g, 68.947 V, 14.105 m/min, 14 us, 6
Gear Profile		
8-teeth gear addendum	OFF3 ON1 WA2 WT1 OV2 WF3	20 us, 350 ns, 6, 500 g, 78.421 V, 14.105 m/min,
8-teeth gear dedendum	WT1 WF1 OFF2 OV3 ON2 WA1	500 g, 6.737 m/min, 14 us, 68.947 V, 300 ns, 4
8-teeth gear circular width	WA2 OV1 OFF2 WT3 WF2 ON3	6, 92.632 V, 14 us, 800 g, 10.421 m/min, 100 ns
4-teeth gear addendum	WT1 WF1 WA1 OFF2 ON1 OV3	500 g, 6.737 m/min, 4, 14 us, 350 ns, 68.947 V,
4-teeth gear dedendum	WT1 OFF2 ON2 OV2 WA1 WF2	500 g, 14 us, 300 ns, 78.421 V, 4, 10.421 m/min
4-teeth gear circular width	OFF1 WA3 WT1 OV3 ON1 WF1	12 us, 8, 500 g, 68.947 V, 350 ns, 6.737 m/min

## Data Availability

Data are available through this paper and Supplementary Materials. Additional data will be available on request.

## Supplementary Materials

The following supporting information can be downloaded at https://www.mdpi.com/article/10.3390/mi16050547/s1.
