# Supplementary material for: Study of Corner and Shape Accuracies in Wire Electro-Discharge Machining of Fin and Gear Profiles and Taper Cutting
Source: Micromachines (Basel). 2025 Apr 30;16(5):547. doi: 10.3390/mi16050547 (PMC12114324; doi:10.3390/mi16050547)
Supplement: Supplementary file 1 [file micromachines-16-00547-s001.zip › micromachines-3578309-supplementary.pdf]

Supplementary document with additional data:

Images of all 18 machined samples.

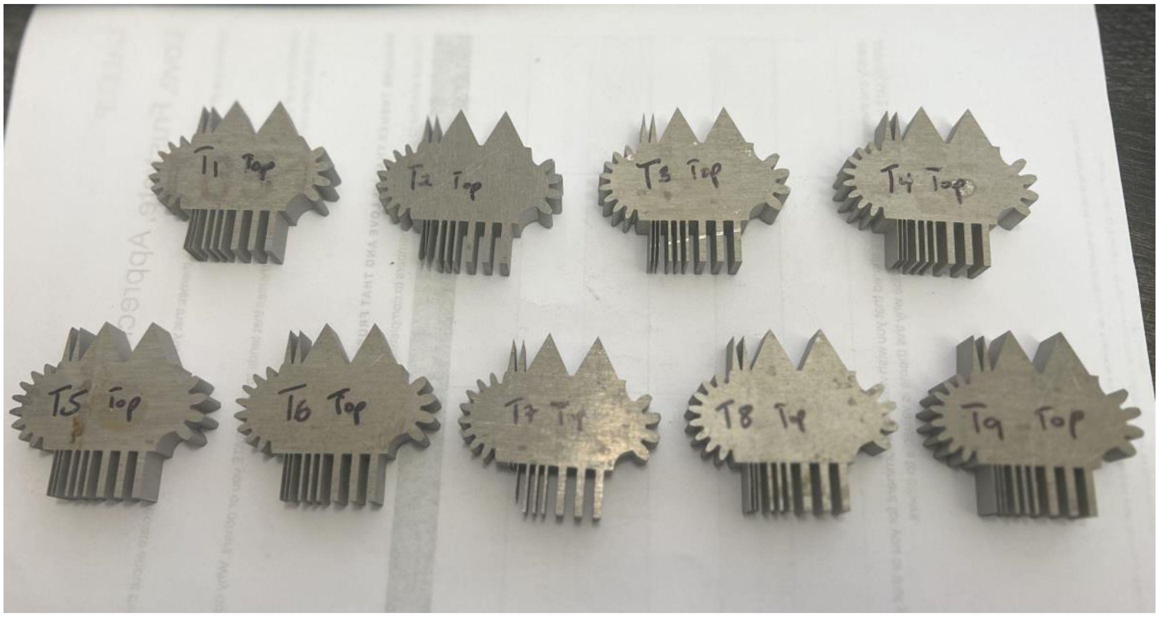

Figure S1. Images of Machined parts for T1 - T9

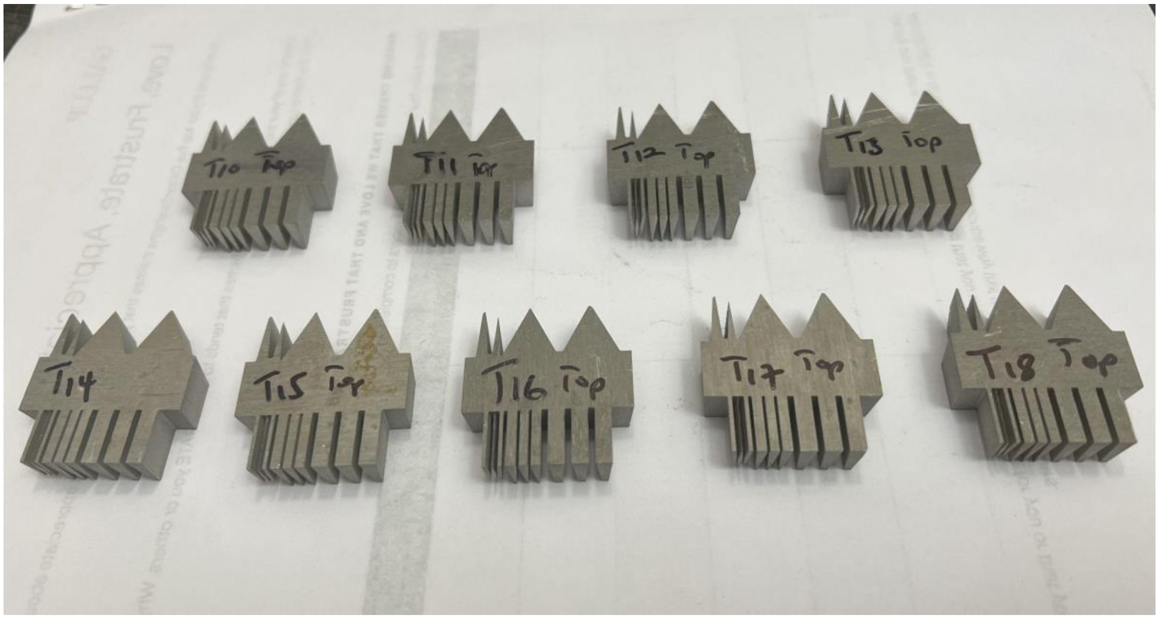

Figure S2. Images of Machined parts for T10 - T18

T1 - T9 Measurements and Response.

Table S1. T1 Measurement and Response

T1 Measurement and Response				
**Table 1: Measured Dimensions of Rectangular Fins Width**				
**Fin width (mm)**	**Fin Number**	**Actual/Intended (mm)**	**Measured (mm)**	**% Deviation**
2	1	2.000	1.673	16.350
	2	2.000	1.677	16.150
	3	2.000	1.692	15.400
1	1	1.000	0.682	31.800
	2	1.000	0.698	30.200
	3	1.000	0.670	33.000
0.5	1	0.500	0.197	60.600
	2	0.500	0.197	60.600
	3	0.500	0.158	68.400
**Table 2: Measured Dimensions of Rectangular Fins Length**				
**Fin length (mm)**	**Fin Number**	**Actual/Intended (mm)**	**Measured (mm)**	**% Deviation**
2	1	10.000	9.893	1.070
	2	10.000	9.893	1.070
	3	10.000	9.893	1.070
1	1	10.000	9.893	1.070
	2	10.000	9.858	1.420
	3	10.000	9.858	1.420
0.5	1	10.000	9.792	2.080

	2	10.000	9.786	2.140
	3	10.000	9.791	2.090

Table 3: Measured Dimensions of Triangular Fins

Fin side length (mm)	Fin Number	Actual/Intended (mm)	Measured (mm)	% Deviation
Small triangular fin	1	9.888	7.813	20.985
	2	9.887	7.798	21.129
Big triangular fin	1	10.938	10.511	3.904
	2	11.030	10.642	3.518

Table 4: Measured Internal and External Angles

Angles (°)	Actual/Intended (°)	Measured (°)	% Deviation
Internal - Small triangular fin	11.693	12.560	7.415
Internal - Big triangular fin	53.753	53.819	0.123
External	117.980	116.789	1.009

Table 5: Measured Dimensions of 8teeth gear Profile

Profile type (mm)	Actual/Intended (mm)	Measured (mm)	% Deviation
Addendum	1.288	1.278	0.776
Dedendum	1.508	1.501	0.464
Tooth thickness/width	1.631	1.542	5.457

Table 6: Measured Dimensions of 4teeth gear Profile

Profile type (mm)	Actual/Intended	Measured (mm)	% Deviation

	(mm)			
Addendum	1.851	1.843	0.432	
Dedendum	1.782	1.755	1.515	
Tooth thickness/width	2.407	2.398	0.374	

Table S2. T2 Measurement and Response

T2 Measurement and Response				
**Table 1: Measured Dimensions of Rectangular Fins Width**				
**Fin width (mm)**	**Fin Number**	**Actual/Intended (mm)**	**Measured (mm)**	**% Deviation**
2	1	2.000	1.909	4.550
	2	2.000	1.684	15.800
	3	2.000	1.692	15.400
1	1	1.000	0.694	30.600
	2	1.000	0.671	32.900
	3	1.000	0.670	33.000
0.5	1	0.500	0.197	60.600
	2	0.500	0.178	64.400
	3	0.500	0.170	66.000
**Table 2: Measured Dimensions of Rectangular Fins Length**				
**Fin length (mm)**	**Fin Number**	**Actual/Intended (mm)**	**Measured (mm)**	**% Deviation**
2	1	10.000	9.901	0.990
	2	10.000	9.913	0.870
	3	10.000	9.913	0.870
1	1	10.000	9.913	0.870
	2	10.000	9.889	1.110

	3	10.000	9.889	1.110
0.5	1	10.000	9.391	6.090
	2	10.000	8.281	17.190
	3	10.000	9.297	7.030

Table 3: Measured Dimensions of Triangular Fins

Fin side length (mm)	Fin Number	Actual/Intended (mm)	Measured (mm)	% Deviation
Small triangular fin	1	9.888	7.694	22.189
	2	9.887	7.792	21.189
Big triangular fin	1	10.938	10.528	3.748
	2	11.030	10.743	2.602

Table 4: Measured Internal and External Angles

Angles (°)	Actual/Intended (°)	Measured (°)	% Deviation
Internal - Small triangular fin	11.693	13.496	15.419
Internal - Big triangular fin	53.753	54.367	1.142
External	117.980	118.856	0.742

Table 5: Measured Dimensions of 8teeth gear Profile

Profile type (mm)	Actual/Intended (mm)	Measured (mm)	% Deviation
Addendum	1.288	1.273	1.165
Dedendum	1.508	1.504	0.265
Tooth thickness/width	1.631	1.598	2.023

Table 6: Measured Dimensions of 4teeth gear Profile				
Profile type (mm)	Actual/Intended (mm)	Measured (mm)	% Deviation	
Addendum	1.851	1.871	1.080	
Dedendum	1.782	1.792	0.561	
Tooth thickness/width	2.407	2.458	2.119	

Table S3. T3 Measurement and Response

T3 Measurement and Response				
**Table 1: Measured Dimensions of Rectangular Fins Width**				
**Fin width (mm)**	**Fin Number**	**Actual/Intended (mm)**	**Measured (mm)**	**% Deviation**
2	1	2.000	1.654	17.300
	2	2.000	1.654	17.283
	3	2.000	1.670	16.500
1	1	1.000	0.688	31.233
	2	1.000	0.652	34.767
	3	1.000	0.656	34.367
0.5	1	0.500	0.164	67.200
	2	0.500	0.180	64.000
	3	0.500	0.154	69.200
**Table 2: Measured Dimensions of Rectangular Fins Length**				
**Fin length (mm)**	**Fin Number**	**Actual/Intended (mm)**	**Measured (mm)**	**% Deviation**
2	1	10.000	9.937	0.630
	2	10.000	9.937	0.630
	3	10.000	9.960	0.400

1	1	10.000	9.913	0.870
	2	10.000	9.889	1.110
	3	10.000	9.866	1.340
0.5	1	10.000	9.825	1.750
	2	10.000	9.824	1.760
	3	10.000	9.780	2.200

Table 3: Measured Dimensions of Triangular Fins

Fin side length (mm)	Fin Number	Actual/Intended (mm)	Measured (mm)	% Deviation
Small triangular fin	1	9.888	7.937	19.731
	2	9.887	8.028	18.802
Big triangular fin	1	10.938	10.477	4.215
	2	11.030	10.744	2.593

Table 4: Measured Internal and External Angles

Angles (°)	Actual/Intended (°)	Measured (°)	% Deviation
Internal - Small triangular fin	11.693	12.293	5.131
Internal - Big triangular fin	53.753	54.189	0.811
External	117.980	117.204	0.658

Table 5: Measured Dimensions of 8teeth gear Profile

Profile type (mm)	Actual/Intended (mm)	Measured (mm)	% Deviation
Addendum	1.288	1.294	0.466
Dedendum	1.508	1.511	0.199

Tooth thickness/width	1.631	1.496	8.277	
**Table 6: Measured Dimensions of 4teeth gear Profile**				
**Profile type (mm)**	**Actual/Intended (mm)**	**Measured (mm)**	**% Deviation**	
Addendum	1.851	1.879	1.513	
Dedendum	1.782	1.812	1.684	
Tooth thickness/width	2.407	2.428	0.872	

Table S4. T4 Measurement and Response

**T4 Measurement and Response**				
**Table 1: Measured Dimensions of Rectangular Fins Width**				
**Fin width (mm)**	**Fin Number**	**Actual/Intended (mm)**	**Measured (mm)**	**% Deviation**
2	1	2.000	1.668	16.600
	2	2.000	1.688	15.600
	3	2.000	1.719	14.033
1	1	1.000	0.738	26.200
	2	1.000	0.667	33.333
	3	1.000	0.689	31.067
0.5	1	0.500	0.175	64.933
	2	0.500	0.174	65.200
	3	0.500	0.163	67.333
**Table 2: Measured Dimensions of Rectangular Fins Length**				
**Fin length (mm)**	**Fin Number**	**Actual/Intended (mm)**	**Measured (mm)**	**% Deviation**
2	1	10.000	9.960	0.400
	2	10.000	9.960	0.400

	3	10.000	9.960	0.400
1	1	10.000	9.913	0.870
	2	10.000	9.937	0.630
	3	10.000	9.901	0.990
0.5	1	10.000	9.824	1.760
	2	10.000	9.801	1.990
	3	10.000	9.795	2.050

Table 3: Measured Dimensions of Triangular Fins

Fin side length (mm)	Fin Number	Actual/Intended (mm)	Measured (mm)	% Deviation
Small triangular fin	1	9.888	7.767	21.450
	2	9.887	7.748	21.634
Big triangular fin	1	10.938	10.556	3.492
	2	11.030	10.726	2.756

Table 4: Measured Internal and External Angles

Angles (°)	Actual/Intended (°)	Measured (°)	% Deviation
Internal - Small triangular fin	11.693	12.709	8.689
Internal - Big triangular fin	53.753	54.431	1.261
External	117.980	116.286	1.436

Table 5: Measured Dimensions of 8teeth gear Profile

Profile type (mm)	Actual/Intended (mm)	Measured (mm)	% Deviation

Addendum	1.288	1.313	1.941	
Dedendum	1.508	1.507	0.066	
Tooth thickness/width	1.631	1.551	4.905	
**Table 6: Measured Dimensions of 4teeth gear Profile**				
**Profile type (mm)**	**Actual/Intended (mm)**	**Measured (mm)**	**% Deviation**	
Addendum	1.851	1.875	1.297	
Dedendum	1.782	1.800	1.010	
Tooth thickness/width	2.407	2.473	2.742	

Table S5. T5 Measurement and Response

**T5 Measurement and Response**				
**Table 1: Measured Dimensions of Rectangular Fins Width**				
**Fin width (mm)**	**Fin Number**	**Actual/Intended (mm)**	**Measured (mm)**	**% Deviation**
2	1	2.000	1.694	15.283
	2	2.000	1.701	14.950
	3	2.000	1.718	14.100
1	1	1.000	0.750	25.000
	2	1.000	0.694	30.567
	3	1.000	0.714	28.600
0.5	1	0.500	0.218	56.333
	2	0.500	0.205	59.067
	3	0.500	0.194	61.267
**Table 2: Measured Dimensions of Rectangular Fins Length**				
**Fin length (mm)**	**Fin Number**	**Actual/Intended (mm)**	**Measured (mm)**	**% Deviation**

2	1	10.000	9.949	0.510
	2	10.000	9.960	0.400
	3	10.000	9.949	0.510
1	1	10.000	9.925	0.750
	2	10.000	9.913	0.870
	3	10.000	9.913	0.870
0.5	1	10.000	9.889	1.110
	2	10.000	9.877	1.230
	3	10.000	9.866	1.340

Table 3: Measured Dimensions of Triangular Fins

Fin side length (mm)	Fin Number	Actual/Intended (mm)	Measured (mm)	% Deviation
Small triangular fin	1	9.888	8.176	17.314
	2	9.887	8.117	17.902
Big triangular fin	1	10.938	10.618	2.926
	2	11.030	10.745	2.584

Table 4: Measured Internal and External Angles

Angles (°)	Actual/Intended (°)	Measured (°)	% Deviation
Internal - Small triangular fin	11.693	12.089	3.387
Internal - Big triangular fin	53.753	54.455	1.306
External	117.980	116.425	1.318

Table 5: Measured Dimensions of 8teeth gear Profile

Profile type (mm)	Actual/Intended (mm)	Measured (mm)	% Deviation
Addendum	1.288	1.283	0.388
Dedendum	1.508	1.495	0.862
Tooth thickness/width	1.631	1.583	2.943

Table 6: Measured Dimensions of 4teeth gear Profile			
**Profile type (mm)**	**Actual/Intended (mm)**	**Measured (mm)**	**% Deviation**
Addendum	1.851	1.932	4.376
Dedendum	1.782	1.843	3.423
Tooth thickness/width	2.407	2.500	3.864

Table S6. T6 Measurement and Response

T6 Measurement and Response				
**Table 1: Measured Dimensions of Rectangular Fins Width**				
**Fin width (mm)**	**Fin Number**	**Actual/Intended (mm)**	**Measured (mm)**	**% Deviation**
2	1	2.000	1.671	16.467
	2	2.000	1.672	16.400
	3	2.000	1.688	15.617
1	1	1.000	0.701	29.900
	2	1.000	0.677	32.267
	3	1.000	0.677	32.267
0.5	1	0.500	0.181	63.867
	2	0.500	0.165	67.067
	3	0.500	0.159	68.133

Table 2: Measured Dimensions of Rectangular Fins Length				
**Fin length (mm)**	**Fin Number**	**Actual/Intended (mm)**	**Measured (mm)**	**% Deviation**
2	1	10.000	9.937	0.630
	2	10.000	9.949	0.510
	3	10.000	9.949	0.510
1	1	10.000	9.937	0.630
	2	10.000	9.937	0.630
	3	10.000	9.937	0.630
0.5	1	10.000	9.901	0.990
	2	10.000	9.854	1.460
	3	10.000	9.794	2.060

Table 3: Measured Dimensions of Triangular Fins				
**Fin side length (mm)**	**Fin Number**	**Actual/Intended (mm)**	**Measured (mm)**	**% Deviation**
Small triangular fin	1	9.888	7.856	20.550
	2	9.887	7.877	20.330
Big triangular fin	1	10.938	10.518	3.840
	2	11.030	10.712	2.883

Table 4: Measured Internal and External Angles			
**Angles (°)**	**Actual/Intended (°)**	**Measured (°)**	**% Deviation**
Internal - Small triangular fin	11.693	12.801	9.476
Internal - Big triangular fin	53.753	54.420	1.241
External	117.980	116.900	0.915

**Table 5: Measured Dimensions of 8teeth gear Profile**			
**Profile type (mm)**	**Actual/Intended (mm)**	**Measured (mm)**	**% Deviation**
Addendum	1.288	1.289	0.078
Dedendum	1.508	1.497	0.729
Tooth thickness/width	1.631	1.554	4.721

**Table 6: Measured Dimensions of 4teeth gear Profile**			
**Profile type (mm)**	**Actual/Intended (mm)**	**Measured (mm)**	**% Deviation**
Addendum	1.851	1.903	2.809
Dedendum	1.782	1.811	1.627
Tooth thickness/width	2.407	2.453	1.911

Table S7. T7 Measurement and Response

**T7 Measurement and Response**				
**Table 1: Measured Dimensions of Rectangular Fins Width**				
**Fin width (mm)**	**Fin Number**	**Actual/Intended (mm)**	**Measured (mm)**	**% Deviation**
2	1	2.000	1.637	18.167
	2	2.000	1.652	17.400
	3	2.000	1.663	16.867
1	1	1.000	0.679	32.133
	2	1.000	0.668	33.200
	3	1.000	0.672	32.800
0.5	1	0.500	severely deformed. part barely machined.	NA
	2	0.500		

	3	0.500	Measurement could not be taken	

Table 2: Measured Dimensions of Rectangular Fins Length

Fin length (mm)	Fin Number	Actual/Intended (mm)	Measured (mm)	% Deviation
2	1	10.000	9.913	0.870
	2	10.000	9.937	0.630
	3	10.000	9.937	0.630
1	1	10.000	9.901	0.990
	2	10.000	9.889	1.110
	3	10.000	9.913	0.870
0.5	1	10.000	severely deformed. part barely machined. Measurement could not be taken	NA
	2	10.000		
	3	10.000		

Table 3: Measured Dimensions of Triangular Fins

Fin side length (mm)	Fin Number	Actual/Intended (mm)	Measured (mm)	% Deviation
Small triangular fin	1	9.888	7.579	23.352
	2	9.887	7.631	22.818
Big triangular fin	1	10.938	10.438	4.571
	2	11.030	10.671	3.255

Table 4: Measured Internal and External Angles

Angles (°)	Actual/Intended (°)	Measured (°)	% Deviation
Internal - Small triangular fin	11.693	13.061	11.699
Internal - Big triangular fin	53.753	54.201	0.833

External	117.980	116.785	1.013

Table 5: Measured Dimensions of 8teeth gear Profile

Profile type (mm)	Actual/Intended (mm)	Measured (mm)	% Deviation
Addendum	1.288	1.281	0.543
Dedendum	1.508	1.514	0.398
Tooth thickness/width	1.631	1.588	2.636

Table 6: Measured Dimensions of 4teeth gear Profile

Profile type (mm)	Actual/Intended (mm)	Measured (mm)	% Deviation
Addendum	1.851	1.906	2.971
Dedendum	1.782	1.831	2.750
Tooth thickness/width	2.407	2.472	2.700

Table S8. T8 Measurement and Response

T8 Measurement and Response

Table 1: Measured Dimensions of Rectangular Fins Width

Fin width (mm)	Fin Number	Actual/Intended (mm)	Measured (mm)	% Deviation
2	1	2.000	1.686	15.700
	2	2.000	1.655	17.267
	3	2.000	1.667	16.667
1	1	1.000	0.679	32.133
	2	1.000	0.684	31.600
	3	1.000	0.669	33.067

0.5	1	0.500	0.167	66.533
	2	0.500	0.175	64.933
	3	0.500	0.170	66.000

Table 2: Measured Dimensions of Rectangular Fins Length

Fin length (mm)	Fin Number	Actual/Intended (mm)	Measured (mm)	% Deviation
2	1	10.000	9.949	0.510
	2	10.000	9.960	0.400
	3	10.000	9.960	0.400
1	1	10.000	9.913	0.870
	2	10.000	9.901	0.990
	3	10.000	9.913	0.870
0.5	1	10.000	9.836	1.640
	2	10.000	9.786	2.140
	3	10.000	9.761	2.390

Table 3: Measured Dimensions of Triangular Fins

Fin side length (mm)	Fin Number	Actual/Intended (mm)	Measured (mm)	% Deviation
Small triangular fin	1	9.888	7.865	20.459
	2	9.887	7.883	20.269
Big triangular fin	1	10.938	10.537	3.666
	2	11.030	10.746	2.575

Table 4: Measured Internal and External Angles

Angles (°)	Actual/Intended (°)	Measured (°)	% Deviation
Internal - Small triangular	11.693	12.545	7.286

fin			
Internal - Big triangular fin	53.753	54.261	0.945
External	117.980	117.707	0.231

Table 5: Measured Dimensions of 8teeth gear Profile

Profile type (mm)	Actual/Intended (mm)	Measured (mm)	% Deviation
Addendum	1.288	1.314	2.019
Dedendum	1.508	1.524	1.061
Tooth thickness/width	1.631	1.527	6.376

Table 6: Measured Dimensions of 4teeth gear Profile

Profile type (mm)	Actual/Intended (mm)	Measured (mm)	% Deviation
Addendum	1.851	1.925	3.998
Dedendum	1.782	1.837	3.086
Tooth thickness/width	2.407	2.390	0.706

Table S9. T9 Measurement and Response

T9 Measurement and Response

Table 1: Measured Dimensions of Rectangular Fins Width

Fin width (mm)	Fin Number	Actual/Intended (mm)	Measured (mm)	% Deviation
2	1	2.000	1.690	15.517
	2	2.000	1.712	14.383
	3	2.000	1.703	14.833
1	1	1.000	0.702	29.833

	2	1.000	0.711	28.867
	3	1.000	0.709	29.067
0.5	1	0.500	0.210	58.067
	2	0.500	0.202	59.533
	3	0.500	0.195	61.067

Table 2: Measured Dimensions of Rectangular Fins Length

Fin length (mm)	Fin Number	Actual/Intended (mm)	Measured (mm)	% Deviation
2	1	10.000	9.949	0.510
	2	10.000	9.937	0.630
	3	10.000	9.937	0.630
1	1	10.000	9.972	0.280
	2	10.000	9.925	0.750
	3	10.000	9.913	0.870
0.5	1	10.000	9.886	1.140
	2	10.000	9.835	1.650
	3	10.000	9.806	1.940

Table 3: Measured Dimensions of Triangular Fins

Fin side length (mm)	Fin Number	Actual/Intended (mm)	Measured (mm)	% Deviation
Small triangular fin	1	9.888	8.131	17.769
	2	9.887	8.138	17.690
Big triangular fin	1	10.938	10.539	3.648
	2	11.030	10.746	2.575

Table 4: Measured Internal and External Angles

Angles (°)	Actual/Intended (°)	Measured (°)	% Deviation
Internal - Small triangular fin	11.693	12.183	4.191
Internal - Big triangular fin	53.753	54.263	0.949
External	117.980	117.738	0.205

Table 5: Measured Dimensions of 8teeth gear Profile

Profile type (mm)	Actual/Intended (mm)	Measured (mm)	% Deviation
Addendum	1.288	1.320	2.484
Dedendum	1.508	1.522	0.928
Tooth thickness/width	1.631	1.626	0.307

Table 6: Measured Dimensions of 4teeth gear Profile

Profile type (mm)	Actual/Intended (mm)	Measured (mm)	% Deviation
Addendum	1.851	1.901	2.701
Dedendum	1.782	1.821	2.189
Tooth thickness/width	2.407	2.510	4.279

T10 - T18 Measurements and Response.

Table S10. T10 Measurement and Response

T10 Measurement and Response

Table 1: Measured Dimensions of Rectangular Fins (5° Taper Cut)

Fin size (mm)	Fin Number	Measured Top Face (mm)	Measured Bottom Face (mm)	Calculated Taper Angle (rad)	Calculated Taper Angle (°)	% Deviation
2	1	2.815	1.203	0.084	4.800	3.996
	2	2.850	1.207	0.085	4.891	2.179
	3	2.854	1.205	0.086	4.909	1.823
1	1	1.706	0.229	0.077	4.399	12.021
	2	1.483	0.235	0.065	3.721	25.580
	3	1.713	0.240	0.077	4.385	12.298
0.5	1	Part defective. Measurement not taken	Part defective. Measurement not taken	NA	NA	NA
	2					
	3					

Table 2: Measured Internal and External Angles (5° Taper Cut)

Angles (°)	Actual/Intended (°)	Measured (°)	% Deviation
Internal - Small triangular fin	11.695	10.951	6.362
Internal - Big triangular fin	54.734	53.069	3.042
External	117.980	116.443	1.303

Table S11. T11 Measurement and Response

T11 Measurement and Response						
**Table 1: Measured Dimensions of Rectangular Fins (5° Taper Cut)**						
**Fin size (mm)**	**Fin Number**	**Measured Top Face (mm)**	**Measured Bottom Face (mm)**	**Calculated Taper Angle (rad)**	**Calculated Taper Angle (°)**	**% Deviation**
2	1	2.837	1.161	0.087	4.989	0.224
	2	2.866	1.181	0.088	5.013	0.270
	3	2.881	1.215	0.087	4.961	0.777
1	1	1.668	0.211	0.076	4.340	13.208
	2	1.473	0.190	0.067	3.822	23.560
	3	1.636	0.190	0.075	4.308	13.841
0.5	1	Part defective. Measurement not taken	Part defective. Measurement not taken	NA	NA	NA
	2					
	3					

**Table 2: Measured Internal and External Angles (5° Taper Cut)**			
**Angles (°)**	**Actual/Intended (°)**	**Measured (°)**	**% Deviation**
Internal - Small triangular fin	11.695	11.382	2.676
Internal - Big triangular fin	54.734	53.201	2.801
External	117.980	116.967	0.859

Table S12. T12 Measurement and Response

T12 Measurement and Response

Table 1: Measured Dimensions of Rectangular Fins (5° Taper Cut)

Fin size (mm)	Fin Number	Measured Top Face (mm)	Measured Bottom Face (mm)	Calculated Taper Angle (rad)	Calculated Taper Angle (°)	% Deviation
2	1	2.845	1.181	0.086	4.952	0.955
	2	2.873	1.199	0.087	4.984	0.323
	3	2.897	1.213	0.088	5.014	0.289
1	1	1.714	0.230	0.077	4.420	11.606
	2	1.501	0.231	0.066	3.783	24.333
	3	1.676	0.222	0.076	4.330	13.406
0.5	1	Part defective. Measurement not taken	Part defective. Measurement not taken	NA	NA	NA
	2					
	3					

Table 2: Measured Internal and External Angles (5° Taper Cut)

Angles (°)	Actual/Intended (°)	Measured (°)	% Deviation
Internal - Small triangular fin	11.695	10.487	10.329
Internal - Big triangular fin	54.734	52.609	3.882
External	117.980	116.504	1.251

Table S13. T13 Measurement and Response

T13 Measurement and Response						
**Table 1: Measured Dimensions of Rectangular Fins (5° Taper Cut)**						
**Fin size (mm)**	**Fin Number**	**Measured Top Face (mm)**	**Measured Bottom Face (mm)**	**Calculated Taper Angle (rad)**	**Calculated Taper Angle (°)**	**% Deviation**
2	1	2.877	1.226	0.086	4.916	1.685
	2	2.893	1.248	0.085	4.897	2.060
	3	2.904	1.248	0.086	4.929	1.429
1	1	1.711	0.258	0.076	4.329	13.426
	2	1.506	0.257	0.065	3.722	25.561
	3	1.685	0.268	0.074	4.222	15.562
0.5	1	Part defective. Measurement not taken	Part defective. Measurement not taken	NA	NA	NA
	2					
	3					

Table 2: Measured Internal and External Angles (5° Taper Cut)			
**Angles (°)**	**Actual/Intended (°)**	**Measured (°)**	**% Deviation**
Internal - Small triangular fin	11.695	10.197	12.809
Internal - Big triangular fin	54.734	52.819	3.499
External	117.980	116.366	1.368

Table S14. T14 Measurement and Response

T14 Measurement and Response						
**Table 1: Measured Dimensions of Rectangular Fins (5° Taper Cut)**						
**Fin size (mm)**	**Fin Number**	**Measured Top Face (mm)**	**Measured Bottom Face (mm)**	**Calculated Taper Angle (rad)**	**Calculated Taper Angle (°)**	**% Deviation**
2	1	2.848	1.199	0.086	4.911	1.784
	2	2.845	1.246	0.083	4.760	4.806
	3	2.851	1.263	0.083	4.730	5.399
1	1	1.677	0.271	0.073	4.189	16.215
	2	1.485	0.279	0.063	3.594	28.117
	3	1.647	0.268	0.072	4.107	17.858
0.5	1	Part defective. Measurement not taken	Part defective. Measurement not taken	NA	NA	NA
	2					
	3					

**Table 2: Measured Internal and External Angles (5° Taper Cut)**			
**Angles (°)**	**Actual/Intended (°)**	**Measured (°)**	**% Deviation**
Internal - Small triangular fin	11.695	10.629	9.115
Internal - Big triangular fin	54.734	52.932	3.292
External	117.980	116.471	1.279

Table S15. T15 Measurement and Response

T15 Measurement and Response						
**Table 1: Measured Dimensions of Rectangular Fins (5° Taper Cut)**						
**Fin size (mm)**	**Fin Number**	**Measured Top Face (mm)**	**Measured Bottom Face (mm)**	**Calculated Taper Angle (rad)**	**Calculated Taper Angle (°)**	**% Deviation**
2	1	2.864	1.202	0.086	4.949	1.014
	2	2.868	1.239	0.085	4.851	2.989
	3	2.875	1.262	0.084	4.801	3.976
1	1	1.706	0.268	0.075	4.284	14.316
	2	1.476	0.224	0.065	3.730	25.402
	3	1.699	0.233	0.076	4.367	12.654
0.5	1	Part defective. Measurement not taken	Part defective. Measurement not taken	NA	NA	NA
	2					
	3					

**Table 2: Measured Internal and External Angles (5° Taper Cut)**			
**Angles (°)**	**Actual/Intended (°)**	**Measured (°)**	**% Deviation**
Internal - Small triangular fin	11.695	10.321	11.749
Internal - Big triangular fin	54.734	53.469	2.311
External	117.980	116.674	1.107

Table S16. T16 Measurement and Response

T16 Measurement and Response

Table 1: Measured Dimensions of Rectangular Fins (5° Taper Cut)

Fin size (mm)	Fin Number	Measured Top Face (mm)	Measured Bottom Face (mm)	Calculated Taper Angle (rad)	Calculated Taper Angle (°)	% Deviation
2	1	2.862	1.207	0.086	4.925	1.508
	2	2.885	1.229	0.086	4.931	1.389
	3	2.888	1.251	0.085	4.873	2.534
1	1	1.709	0.256	0.076	4.328	13.445
	2	1.514	0.240	0.066	3.796	24.075
	3	1.673	0.260	0.073	4.210	15.800
0.5	1	Part defective. Measurement not taken	Part defective. Measurement not taken	NA	NA	NA
	2					
	3					

Table 2: Measured Internal and External Angles (5° Taper Cut)

Angles (°)	Actual/Intended (°)	Measured (°)	% Deviation
Internal - Small triangular fin	11.695	10.302	11.911
Internal - Big triangular fin	54.734	53.144	2.905
External	117.980	116.764	1.031

Table S17. T17 Measurement and Response

T17 Measurement and Response						
**Table 1: Measured Dimensions of Rectangular Fins (5° Taper Cut)**						
**Fin size (mm)**	**Fin Number**	**Measured Top Face (mm)**	**Measured Bottom Face (mm)**	**Calculated Taper Angle (rad)**	**Calculated Taper Angle (°)**	**% Deviation**
2	1	2.889	1.229	0.086	4.940	1.192
	2	2.889	1.237	0.086	4.919	1.626
	3	2.900	1.261	0.085	4.880	2.396
1	1	1.707	0.249	0.076	4.343	13.149
	2	1.509	0.235	0.066	3.795	24.095
	3	1.688	0.243	0.075	4.303	13.940
0.5	1	Part defective. Measurement not taken	Part defective. Measurement not taken	NA	NA	NA
	2					
	3					

Table 2: Measured Internal and External Angles (5° Taper Cut)			
**Angles (°)**	**Actual/Intended (°)**	**Measured (°)**	**% Deviation**
Internal - Small triangular fin	11.695	10.446	10.680
Internal - Big triangular fin	54.734	53.510	2.236
External	117.980	116.517	1.240

Table S18. T18 Measurement and Response

T18 Measurement and Response						
**Table 1: Measured Dimensions of Rectangular Fins (5° Taper Cut)**						
**Fin size (mm)**	**Fin Number**	**Measured Top Face (mm)**	**Measured Bottom Face (mm)**	**Calculated Taper Angle (rad)**	**Calculated Taper Angle (°)**	**% Deviation**
2	1	2.856	1.203	0.086	4.923	1.547
	2	2.868	1.205	0.086	4.952	0.955
	3	2.872	1.217	0.086	4.927	1.468
1	1	1.696	0.231	0.076	4.361	12.773
	2	1.488	0.235	0.065	3.733	25.343
	3	1.661	0.247	0.074	4.213	15.740
0.5	1	Part defective. Measurement not taken	Part defective. Measurement not taken	NA	NA	NA
	2					
	3					

**Table 2: Measured Internal and External Angles (5° Taper Cut)**			
**Angles (°)**	**Actual/Intended (°)**	**Measured (°)**	**% Deviation**
Internal - Small triangular fin	11.695	10.675	8.722
Internal - Big triangular fin	54.734	52.789	3.554
External	117.980	117.868	0.095

Rectangular Fin Width Response Table

Table S19. Response table for 2mm rectangular fin width

	Response Totals for 2 mm rectangular fin width						
Straight Cut	Factors	Wire Tension (WT)	Feed Rate (WF)	Flushing Pressure (WA)	Pulse On-Time (ON)	Pulse Off-Time (OFF) us	Voltage of open circuit (OV)
	Level 1	134.733	146.567	138.866	148.818	146.018	136.966
	Level 2	139.050	129.717	132.517	131.617	126.716	136.668
	Level 3	146.801	144.300	149.201	140.149	147.850	146.950
	Average Response						
	Factors	Wire Tension (WT)	Feed Rate (WF)	Flushing Pressure (WA)	Pulse On-Time (ON)	Pulse Off-Time (OFF) us	Voltage of open circuit (OV)
	Level 1	14.970	16.285	15.430	16.535	16.224	15.218
	Level 2	15.450	14.413	14.724	14.624	14.080	15.185
	Level 3	16.311	16.033	16.578	15.572	16.428	16.328
	Difference	1.341	1.872	1.854	1.911	2.348	1.142
	**Rank**	**5**	**3**	**4**	**2**	**1**	**6**

Table S20. Response table for 1mm rectangular fin width

	Response Totals for 1 mm rectangular fin width						
Straight Cut	Factors	**Wire Tension (WT)**	**Feed Rate (WF)**	**Flushing Pressure (WA)**	**Pulse On-Time (ON)**	**Pulse Off-Time (OFF) us**	**Voltage of open circuit (OV)**
	Level 1	291.867	283.733	273.367	287.567	286.234	266.934
	Level 2	269.201	277.467	278.800	283.900	274.867	289.067
	Level 3	282.700	282.568	291.601	272.301	282.667	287.767
	Average Response						
	Factors	**Wire Tension (WT)**	**Feed Rate (WF)**	**Flushing Pressure (WA)**	**Pulse On-Time (ON)**	**Pulse Off-Time (OFF) us**	**Voltage of open circuit (OV)**
	Level 1	32.430	31.526	30.374	31.952	31.804	29.659
	Level 2	29.911	30.830	30.978	31.544	30.541	32.119
	Level 3	31.411	31.396	32.400	30.256	31.407	31.974
	Difference	2.518	0.696	2.026	1.696	1.263	2.459
	**Rank**	**1**	**6**	**3**	**4**	**5**	**2**

Table S21. Response table for 0.5mm rectangular fin width

	Response Totals for 0.5 mm rectangular fin width						
**Straight Cut**	Factors	**Wire Tension (WT)**	**Feed Rate (WF)**	**Flushing Pressure (WA)**	**Pulse On-Time (ON)**	**Pulse Off-Time (OFF) us**	**Voltage of open circuit (OV)**
	Level 1	581.000	687.066	565.733	688.667	586.133	544.934
	Level 2	573.200	565.133	667.667	585.932	567.133	690.067
	Level 3	676.133	578.134	596.933	555.734	677.067	595.332
	Average Response						
	Factors	**Wire Tension (WT)**	**Feed Rate (WF)**	**Flushing Pressure (WA)**	**Pulse On-Time (ON)**	**Pulse Off-Time (OFF) us**	**Voltage of open circuit (OV)**
	Level 1	64.556	76.341	62.859	76.519	65.126	60.548
	Level 2	63.689	62.793	74.185	65.104	63.015	76.674
	Level 3	75.126	64.237	66.326	61.748	75.230	66.148
	Difference	11.437	13.548	11.326	14.770	12.215	16.126
	**Rank**	**5**	**3**	**6**	**2**	**4**	**1**

Table S22. Response table for 2mm rectangular fin width

**Taper Cut**	Response Totals for 2 mm rectangular fin width						
	Factors	**Wire Tension (WT)**	**Feed Rate (WF)**	**Flushing Pressure (WA)**	**Pulse On-Time (ON)**	**Pulse Off-Time (OFF) us**	**Voltage of open circuit (OV)**
	Level 1	10.836	18.603	14.464	17.230	11.955	18.987
	Level 2	25.142	18.474	10.711	14.977	23.957	21.191
	Level 3	14.615	13.516	25.418	18.386	14.681	10.415
	Average Response						
	Factors	**Wire Tension (WT)**	**Feed Rate (WF)**	**Flushing Pressure (WA)**	**Pulse On-Time (ON)**	**Pulse Off-Time (OFF) us**	**Voltage of open circuit (OV)**
	Level 1	1.204	2.067	1.607	1.914	1.328	2.110
	Level 2	2.794	2.053	1.190	1.664	2.662	2.355
	Level 3	1.624	1.502	2.824	2.043	1.631	1.157
	Difference	1.590	0.565	1.634	0.379	1.334	1.197
	**Rank**	**2**	**5**	**1**	**6**	**3**	**4**

Table S23. Response table for 1mm rectangular fin width

	Response Totals for 1 mm rectangular fin width						
**Taper Cut**	Factors	**Wire Tension (WT)**	**Feed Rate (WF)**	**Flushing Pressure (WA)**	**Pulse On-Time (ON)**	**Pulse Off-Time (OFF) us**	**Voltage of open circuit (OV)**
	Level 1	149.853	157.768	154.165	166.655	155.078	164.855
	Level 2	169.111	163.983	157.750	155.037	165.945	153.455
	Level 3	158.360	155.573	165.409	155.632	156.301	159.014
	Average Response						
	Factors	**Wire Tension (WT)**	**Feed Rate (WF)**	**Flushing Pressure (WA)**	**Pulse On-Time (ON)**	**Pulse Off-Time (OFF) us**	**Voltage of open circuit (OV)**
	Level 1	16.650	17.530	17.129	18.517	17.231	18.317
	Level 2	18.790	18.220	17.528	17.226	18.438	17.051
	Level 3	17.596	17.286	18.379	17.292	17.367	17.668
	Difference	2.140	0.934	1.249	1.291	1.207	1.267
	**Rank**	**1**	**6**	**4**	**2**	**5**	**3**

Rectangular Fin Length Response Table

Table S24. Response table for 2mm rectangular fin length

	Response Totals for 2 mm rectangular fin length						
Straight Cut	Factors	**Wire Tension (WT)**	**Feed Rate (WF)**	**Flushing Pressure (WA)**	**Pulse On-Time (ON)**	**Pulse Off-Time (OFF) us**	**Voltage of open circuit (OV)**
	Level 1	7.600	6.540	6.180	6.990	6.170	6.400
	Level 2	4.270	5.460	6.280	5.240	5.700	6.510
	Level 3	5.210	5.080	4.620	4.850	5.210	4.170
	Average Response						
	Factors	**Wire Tension (WT)**	**Feed Rate (WF)**	**Flushing Pressure (WA)**	**Pulse On-Time (ON)**	**Pulse Off-Time (OFF) us**	**Voltage of open circuit (OV)**
	Level 1	0.844	0.727	0.687	0.777	0.686	0.711
	Level 2	0.474	0.607	0.698	0.582	0.633	0.723
	Level 3	0.579	0.564	0.513	0.539	0.579	0.463
	Difference	0.370	0.162	0.184	0.238	0.107	0.260
	**Rank**	**1**	**5**	**4**	**3**	**6**	**2**

Table S25. Response table for 1mm rectangular fin length

	Response Totals for 1 mm rectangular fin length						
**Straight Cut**	Factors	**Wire Tension (WT)**	**Feed Rate (WF)**	**Flushing Pressure (WA)**	**Pulse On-Time (ON)**	**Pulse Off-Time (OFF) us**	**Voltage of open circuit (OV)**
	Level 1	10.320	9.370	8.300	8.770	8.530	8.300
	Level 2	6.870	8.310	8.550	8.310	7.480	7.950
	Level 3	7.600	7.110	7.940	7.710	8.780	8.540
	Average Response						
	Factors	**Wire Tension (WT)**	**Feed Rate (WF)**	**Flushing Pressure (WA)**	**Pulse On-Time (ON)**	**Pulse Off-Time (OFF) us**	**Voltage of open circuit (OV)**
	Level 1	1.147	1.041	0.922	0.974	0.948	0.922
	Level 2	0.763	0.923	0.950	0.923	0.831	0.883
	Level 3	0.844	0.790	0.882	0.857	0.976	0.949
	Difference	0.383	0.251	0.068	0.118	0.144	0.066
	**Rank**	**1**	**2**	**5**	**4**	**3**	**6**

Table S26. Response table for 0.5mm rectangular fin length

Straight Cut	Response Totals for 0.5 mm rectangular fin length						
	Factors	**Wire Tension (WT)**	**Feed Rate (WF)**	**Flushing Pressure (WA)**	**Pulse On-Time (ON)**	**Pulse Off-Time (OFF) us**	**Voltage of open circuit (OV)**
	Level 1	42.330	312.110	16.840	310.820	16.990	14.720
	Level 2	13.990	40.160	42.280	42.280	40.840	334.820
	Level 3	310.900	14.950	16.390	14.120	309.390	17.680
	Average Response						
	Factors	**Wire Tension (WT)**	**Feed Rate (WF)**	**Flushing Pressure (WA)**	**Pulse On-Time (ON)**	**Pulse Off-Time (OFF) us**	**Voltage of open circuit (OV)**
	Level 1	4.703	34.679	1.871	34.536	1.888	1.636
	Level 2	1.554	4.462	4.698	4.698	4.538	37.202
	Level 3	34.544	1.661	1.821	1.569	34.377	1.964
	Difference	32.990	33.018	2.877	32.967	32.489	35.567
	**Rank**	**3**	**2**	**6**	**4**	**5**	**1**

Triangular Fin Length Response Table

Table S27. Response table for small triangular fin length

	Response Totals for Small Triangular fin length						
	Factors	**Wire Tension (WT)**	**Feed Rate (WF)**	**Flushing Pressure (WA)**	**Pulse On-Time (ON)**	**Pulse Off-Time (OFF) us**	**Voltage of open circuit (OV)**
	Level 1	124.025	131.368	120.657	129.164	123.722	112.789
	Level 2	119.180	119.322	124.764	127.190	121.921	130.428
	Level 3	122.357	114.872	120.141	109.208	119.919	122.345
**Straight Cut**							
	Average Response						
	Factors	**Wire Tension (WT)**	**Feed Rate (WF)**	**Flushing Pressure (WA)**	**Pulse On-Time (ON)**	**Pulse Off-Time (OFF) us**	**Voltage of open circuit (OV)**
	Level 1	20.671	21.895	20.110	21.527	20.620	18.798
	Level 2	19.863	19.887	20.794	21.198	20.320	21.738
	Level 3	20.393	19.145	20.024	18.201	19.987	20.391
	Difference	0.808	2.749	0.770	3.326	0.634	2.940
	**Rank**	**4**	**3**	**5**	**1**	**6**	**2**

Table S28. Response table for big triangular fin length

	Response Totals for Big Triangular fin length						
Straight Cut	Factors	**Wire Tension (WT)**	**Feed Rate (WF)**	**Flushing Pressure (WA)**	**Pulse On-Time (ON)**	**Pulse Off-Time (OFF) us**	**Voltage of open circuit (OV)**
	Level 1	20.580	21.496	19.893	21.971	20.386	19.155
	Level 2	18.481	18.101	19.686	18.839	18.821	20.899
	Level 3	20.290	19.754	19.772	18.541	20.144	19.297
	Average Response						
	Factors	**Wire Tension (WT)**	**Feed Rate (WF)**	**Flushing Pressure (WA)**	**Pulse On-Time (ON)**	**Pulse Off-Time (OFF) us**	**Voltage of open circuit (OV)**
	Level 1	3.430	3.583	3.316	3.662	3.398	3.193
	Level 2	3.080	3.017	3.281	3.140	3.137	3.483
	Level 3	3.382	3.292	3.295	3.090	3.357	3.216
	Difference	0.350	0.566	0.035	0.572	0.261	0.291
	**Rank**	**3**	**2**	**6**	**1**	**5**	**4**

Internal and External Angles Response Table.

Table S29. Response table for 11.693 (°) internal angle

	Response Totals for 11.693 (°) Internal angle						
Straight Cut	Factors	**Wire Tension (WT)**	**Feed Rate (WF)**	**Flushing Pressure (WA)**	**Pulse On-Time (ON)**	**Pulse Off-Time (OFF) us**	**Voltage of open circuit (OV)**
	Level 1	27.965	27.803	20.295	28.590	24.177	14.993
	Level 2	21.552	26.092	30.505	31.394	28.299	36.594
	Level 3	23.176	18.798	21.893	12.709	20.217	21.106
	Average Response						
	Factors	**Wire Tension (WT)**	**Feed Rate (WF)**	**Flushing Pressure (WA)**	**Pulse On-Time (ON)**	**Pulse Off-Time (OFF) us**	**Voltage of open circuit (OV)**
	Level 1	9.322	9.268	6.765	9.530	8.059	4.998
	Level 2	7.184	8.697	10.168	10.465	9.433	12.198
	Level 3	7.725	6.266	7.298	4.236	6.739	7.035
	Difference	2.138	3.002	3.403	6.228	2.694	7.200
	**Rank**	**6**	**4**	**3**	**2**	**5**	**1**

Table S30. Response table for 53.753 (°) Internal angle

	Response Totals for 53.753 (°) Internal angle						
**Straight Cut**	Factors	**Wire Tension (WT)**	**Feed Rate (WF)**	**Flushing Pressure (WA)**	**Pulse On-Time (ON)**	**Pulse Off-Time (OFF) us**	**Voltage of open circuit (OV)**
	Level 1	2.076	2.217	2.333	2.197	2.309	2.378
	Level 2	3.808	3.393	3.281	3.348	3.352	3.216
	Level 3	2.727	3.001	2.997	3.066	2.950	3.017
	Average Response						
	Factors	**Wire Tension (WT)**	**Feed Rate (WF)**	**Flushing Pressure (WA)**	**Pulse On-Time (ON)**	**Pulse Off-Time (OFF) us**	**Voltage of open circuit (OV)**
	Level 1	0.692	0.739	0.778	0.732	0.770	0.793
	Level 2	1.269	1.131	1.094	1.116	1.117	1.072
	Level 3	0.909	1.000	0.999	1.022	0.983	1.006
	Difference	0.577	0.392	0.316	0.384	0.348	0.279
	**Rank**	**1**	**2**	**5**	**3**	**4**	**6**

Table S31. Response table for 117.98 (°) External angle

	Response Totals for 117.98 (°) External angle						
**Straight Cut**	Factors	**Wire Tension (WT)**	**Feed Rate (WF)**	**Flushing Pressure (WA)**	**Pulse On-Time (ON)**	**Pulse Off-Time (OFF) us**	**Voltage of open circuit (OV)**
	Level 1	2.409	3.458	2.650	2.937	2.155	2.532
	Level 2	3.669	2.291	3.073	2.409	2.383	2.670
	Level 3	1.449	1.778	1.804	2.181	2.989	2.325
	Average Response						
	Factors	**Wire Tension (WT)**	**Feed Rate (WF)**	**Flushing Pressure (WA)**	**Pulse On-Time (ON)**	**Pulse Off-Time (OFF) us**	**Voltage of open circuit (OV)**
	Level 1	0.803	1.153	0.883	0.979	0.718	0.844
	Level 2	1.223	0.764	1.024	0.803	0.794	0.890
	Level 3	0.483	0.593	0.601	0.727	0.996	0.775
	Difference	0.740	0.560	0.423	0.252	0.278	0.115
	**Rank**	**1**	**2**	**3**	**5**	**4**	**6**

Table S32. Response table for 11.693 (°) internal angle

	Response Totals for 11.693 (°) Internal angle						
	Factors	**Wire Tension (WT)**	**Feed Rate (WF)**	**Flushing Pressure (WA)**	**Pulse On-Time (ON)**	**Pulse Off-Time (OFF) us**	**Voltage of open circuit (OV)**
	Level 1	19.367	31.082	25.105	20.513	33.818	31.355
	Level 2	33.673	22.471	31.860	33.989	24.199	28.791
	Level 3	31.313	30.800	27.388	29.851	26.336	24.207
**Taper Cut**	Average Response						
	Factors	**Wire Tension (WT)**	**Feed Rate (WF)**	**Flushing Pressure (WA)**	**Pulse On-Time (ON)**	**Pulse Off-Time (OFF) us**	**Voltage of open circuit (OV)**
	Level 1	6.456	10.361	8.368	6.838	11.273	10.452
	Level 2	11.224	7.490	10.620	11.330	8.066	9.597
	Level 3	10.438	10.267	9.129	9.950	8.779	8.069
	Difference	4.769	2.870	2.252	4.492	3.206	2.383
	**Rank**	**1**	**4**	**6**	**2**	**3**	**5**

Table S33. Response table for 53.753 (°) Internal angle

	Response Totals for 53.753 (°) Internal angle						
**Taper Cut**	Factors	**Wire Tension (WT)**	**Feed Rate (WF)**	**Flushing Pressure (WA)**	**Pulse On-Time (ON)**	**Pulse Off-Time (OFF) us**	**Voltage of open circuit (OV)**
	Level 1	9.725	9.446	7.348	9.647	9.617	10.079
	Level 2	9.102	8.329	10.935	9.098	9.888	7.589
	Level 3	8.695	9.747	9.239	8.777	8.017	9.854
	Average Response						
	Factors	**Wire Tension (WT)**	**Feed Rate (WF)**	**Flushing Pressure (WA)**	**Pulse On-Time (ON)**	**Pulse Off-Time (OFF) us**	**Voltage of open circuit (OV)**
	Level 1	3.242	3.149	2.449	3.216	3.206	3.360
	Level 2	3.034	2.776	3.645	3.033	3.296	2.530
	Level 3	2.898	3.249	3.080	2.926	2.672	3.285
	Difference	0.343	0.473	1.196	0.290	0.624	0.830
	**Rank**	**5**	**4**	**1**	**6**	**3**	**2**

Table S34. Response table for 117.98 (°) External angle

	Response Totals for 117.98 (°) External angle						
Taper Cut	Factors	**Wire Tension (WT)**	**Feed Rate (WF)**	**Flushing Pressure (WA)**	**Pulse On-Time (ON)**	**Pulse Off-Time (OFF) us**	**Voltage of open circuit (OV)**
	Level 1	3.413	3.702	3.206	2.233	3.859	3.561
	Level 2	3.754	3.378	2.714	3.389	2.677	3.650
	Level 3	2.366	2.453	3.613	3.911	2.997	2.322
	Average Response						
	Factors	**Wire Tension (WT)**	**Feed Rate (WF)**	**Flushing Pressure (WA)**	**Pulse On-Time (ON)**	**Pulse Off-Time (OFF) us**	**Voltage of open circuit (OV)**
	Level 1	1.138	1.234	1.069	0.744	1.286	1.187
	Level 2	1.251	1.126	0.905	1.130	0.892	1.217
	Level 3	0.789	0.818	1.204	1.304	0.999	0.774
	Difference	0.463	0.416	0.300	0.559	0.394	0.443
	**Rank**	**2**	**4**	**6**	**1**	**5**	**3**

Table S35. Response Table for 8 teeth Gear % Dev. Addendum

	Response Totals for % Dev. Addendum 8 teeth Gear						
**Straight Cut**	Factors	**Wire Tension (WT)**	**Feed Rate (WF)**	**Flushing Pressure (WA)**	**Pulse On-Time (ON)**	**Pulse Off-Time (OFF) us**	**Voltage of open circuit (OV)**
	Level 1	2.407	3.260	5.201	1.397	2.873	3.648
	Level 2	2.407	3.572	2.096	5.125	5.590	1.786
	Level 3	5.046	3.028	2.563	3.338	1.397	4.426
	Average Response						
	Factors	**Wire Tension (WT)**	**Feed Rate (WF)**	**Flushing Pressure (WA)**	**Pulse On-Time (ON)**	**Pulse Off-Time (OFF) us**	**Voltage of open circuit (OV)**
	Level 1	0.802	1.087	1.734	0.466	0.958	1.216
	Level 2	0.802	1.191	0.699	1.708	1.863	0.595
	Level 3	1.682	1.009	0.854	1.113	0.466	1.475
	Difference	0.880	0.181	1.035	1.243	1.398	0.880
	**Rank**	**4**	**6**	**3**	**2**	**1**	**4**

Table S36. Response Table for 8 teeth Gear % Dev. Dedendum

**Straight Cut**	**Response Totals for % Dev. Dedendum 8 teeth Gear**						
	Factors	**Wire Tension (WT)**	**Feed Rate (WF)**	**Flushing Pressure (WA)**	**Pulse On-Time (ON)**	**Pulse Off-Time (OFF) us**	**Voltage of open circuit (OV)**
	Level 1	0.928	0.928	1.458	1.591	2.254	2.254
	Level 2	1.657	2.188	1.525	1.392	1.259	1.392
	Level 3	2.387	1.856	1.989	1.989	1.459	1.326
	Average Response						
	Factors	**Wire Tension (WT)**	**Feed Rate (WF)**	**Flushing Pressure (WA)**	**Pulse On-Time (ON)**	**Pulse Off-Time (OFF) us**	**Voltage of open circuit (OV)**
	Level 1	0.309	0.309	0.486	0.530	0.751	0.751
	Level 2	0.552	0.729	0.508	0.464	0.420	0.464
	Level 3	0.796	0.619	0.663	0.663	0.486	0.442
	Difference	0.486	0.420	0.177	0.199	0.332	0.309
	**Rank**	**1**	**2**	**6**	**5**	**3**	**4**

Table S37. Response Table for 8 teeth Gear % Dev. Circular Width

	Response Totals for % Dev. Circular Width 8 teeth Gear						
**Straight Cut**	Factors	**Wire Tension (WT)**	**Feed Rate (WF)**	**Flushing Pressure (WA)**	**Pulse On-Time (ON)**	**Pulse Off-Time (OFF) us**	**Voltage of open circuit (OV)**
	Level 1	15.757	12.998	10.669	12.814	16.554	8.707
	Level 2	12.569	11.342	7.602	13.304	7.235	9.380
	Level 3	9.319	13.305	19.374	11.527	13.856	19.558
	Average Response						
	Factors	**Wire Tension (WT)**	**Feed Rate (WF)**	**Flushing Pressure (WA)**	**Pulse On-Time (ON)**	**Pulse Off-Time (OFF) us**	**Voltage of open circuit (OV)**
	Level 1	5.252	4.333	3.556	4.271	5.518	2.902
	Level 2	4.190	3.781	2.534	4.435	2.412	3.127
	Level 3	3.106	4.435	6.458	3.842	4.619	6.519
	Difference	2.146	0.654	3.924	0.592	3.106	3.617
	**Rank**	**4**	**5**	**1**	**6**	**3**	**2**

Table S38. Response Table for 4 teeth Gear % Dev. Addendum

	Response Totals for % Dev. Addendum 4 teeth Gear						
**Straight Cut**	Factors	**Wire Tension (WT)**	**Feed Rate (WF)**	**Flushing Pressure (WA)**	**Pulse On-Time (ON)**	**Pulse Off-Time (OFF) us**	**Voltage of open circuit (OV)**
	Level 1	3.025	4.700	4.430	6.212	7.239	7.509
	Level 2	8.482	9.454	8.427	6.375	5.078	6.860
	Level 3	9.670	7.023	8.320	8.590	8.860	6.808
	Average Response						
	Factors	**Wire Tension (WT)**	**Feed Rate (WF)**	**Flushing Pressure (WA)**	**Pulse On-Time (ON)**	**Pulse Off-Time (OFF) us**	**Voltage of open circuit (OV)**
	Level 1	1.008	1.567	1.477	2.071	2.413	2.503
	Level 2	2.827	3.151	2.809	2.125	1.693	2.287
	Level 3	3.223	2.341	2.773	2.863	2.953	2.269
	Difference	2.215	1.585	1.332	0.793	1.261	0.234
	**Rank**	**1**	**2**	**3**	**5**	**4**	**6**

Table S39. Response Table for 4 teeth Gear % Dev. Dedendum

**Straight Cut**	**Response Totals for % Dev. Dedendum 4 teeth Gear**						
	Factors	**Wire Tension (WT)**	**Feed Rate (WF)**	**Flushing Pressure (WA)**	**Pulse On-Time (ON)**	**Pulse Off-Time (OFF) us**	**Voltage of open circuit (OV)**
	Level 1	3.760	5.275	4.714	5.892	6.228	7.127
	Level 2	6.060	7.070	6.734	4.657	3.760	4.938
	Level 3	8.025	5.500	6.397	7.296	7.857	5.780
	Average Response						
	Factors	**Wire Tension (WT)**	**Feed Rate (WF)**	**Flushing Pressure (WA)**	**Pulse On-Time (ON)**	**Pulse Off-Time (OFF) us**	**Voltage of open circuit (OV)**
	Level 1	1.253	1.758	1.571	1.964	2.076	2.376
	Level 2	2.020	2.357	2.245	1.552	1.253	1.646
	Level 3	2.675	1.833	2.132	2.432	2.619	1.927
	Difference	1.422	0.598	0.673	0.880	1.366	0.730
	**Rank**	**1**	**6**	**5**	**3**	**2**	**4**

Table S40. Response Table for 4 teeth Gear % Dev. Circular Width

	Response Totals for % Dev. Circular Width 4 teeth Gear						
	Factors	**Wire Tension (WT)**	**Feed Rate (WF)**	**Flushing Pressure (WA)**	**Pulse On-Time (ON)**	**Pulse Off-Time (OFF) us**	**Voltage of open circuit (OV)**
	Level 1	3.365	5.816	7.395	4.985	2.991	8.517
	Level 2	8.517	6.689	8.683	8.683	9.140	6.730
	Level 3	7.685	7.062	3.489	9.015	7.436	4.320
**Straight Cut**							
	Average Response						
	Factors	**Wire Tension (WT)**	**Feed Rate (WF)**	**Flushing Pressure (WA)**	**Pulse On-Time (ON)**	**Pulse Off-Time (OFF) us**	**Voltage of open circuit (OV)**
	Level 1	1.122	1.939	2.465	1.662	0.997	2.839
	Level 2	2.839	2.230	2.894	2.894	3.047	2.243
	Level 3	2.562	2.354	1.163	3.005	2.479	1.440
	Difference	1.717	0.415	1.731	1.343	2.050	1.399
	**Rank**	**3**	**6**	**2**	**5**	**1**	**4**

Tables and Figures for auxiliary Experiment on Effect of Workpiece Thickness.

Table S41. WEDM parameters used for auxiliary experiments studying effect of Workpiece thickness

**Cutting Condition**	**Description/value (Unit)**
NO	3151
PM	10
OV	10 (92.63 V)
ON	7 (350 ns)
OFF	12 µs
AON	4 (200 ns)
AOFF	12 (11.17 µs)
SV	42V
WT	3 (502.11g)
WF	2 (6.74 meter/min)
WA	4
FR%	100 %
F	1.9685 mm^2/min
SM	M90
SC	14

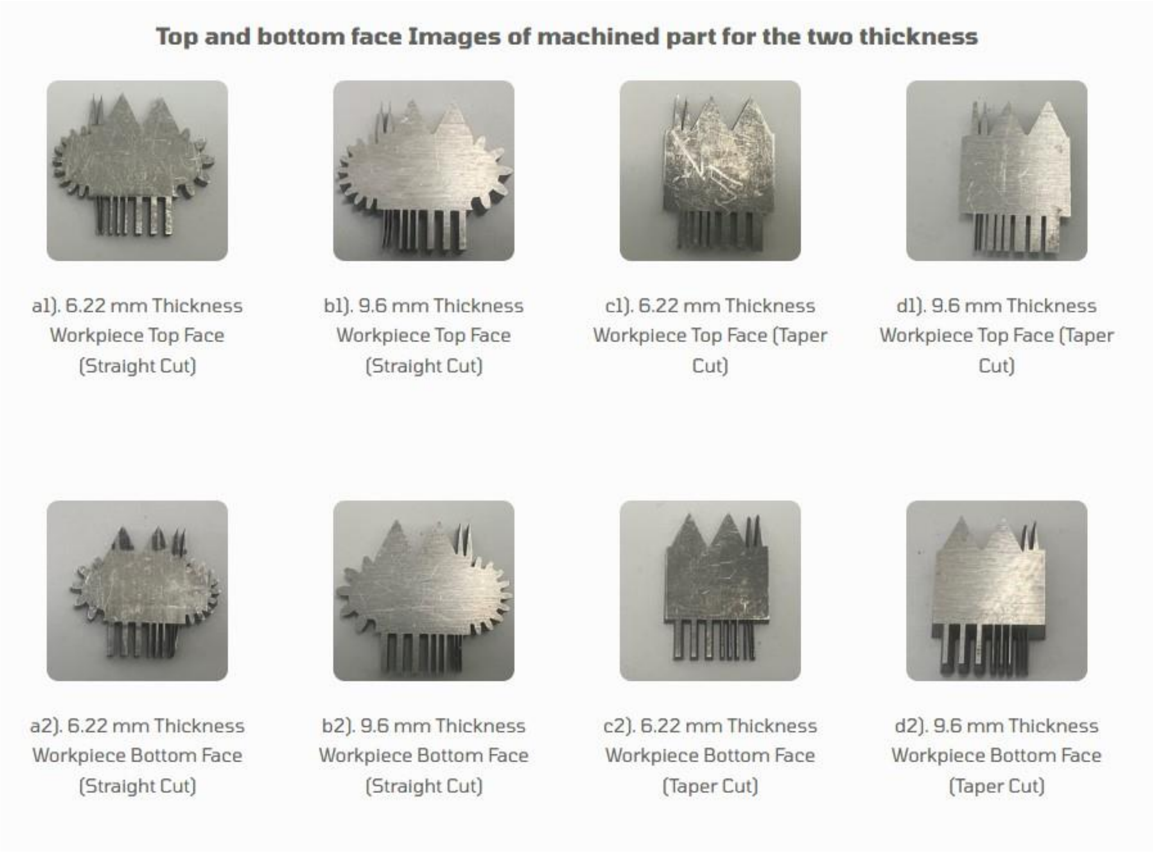

Figure S3. Top and bottom Images of machined parts for 6.22 mm and 9.6 mm thickness

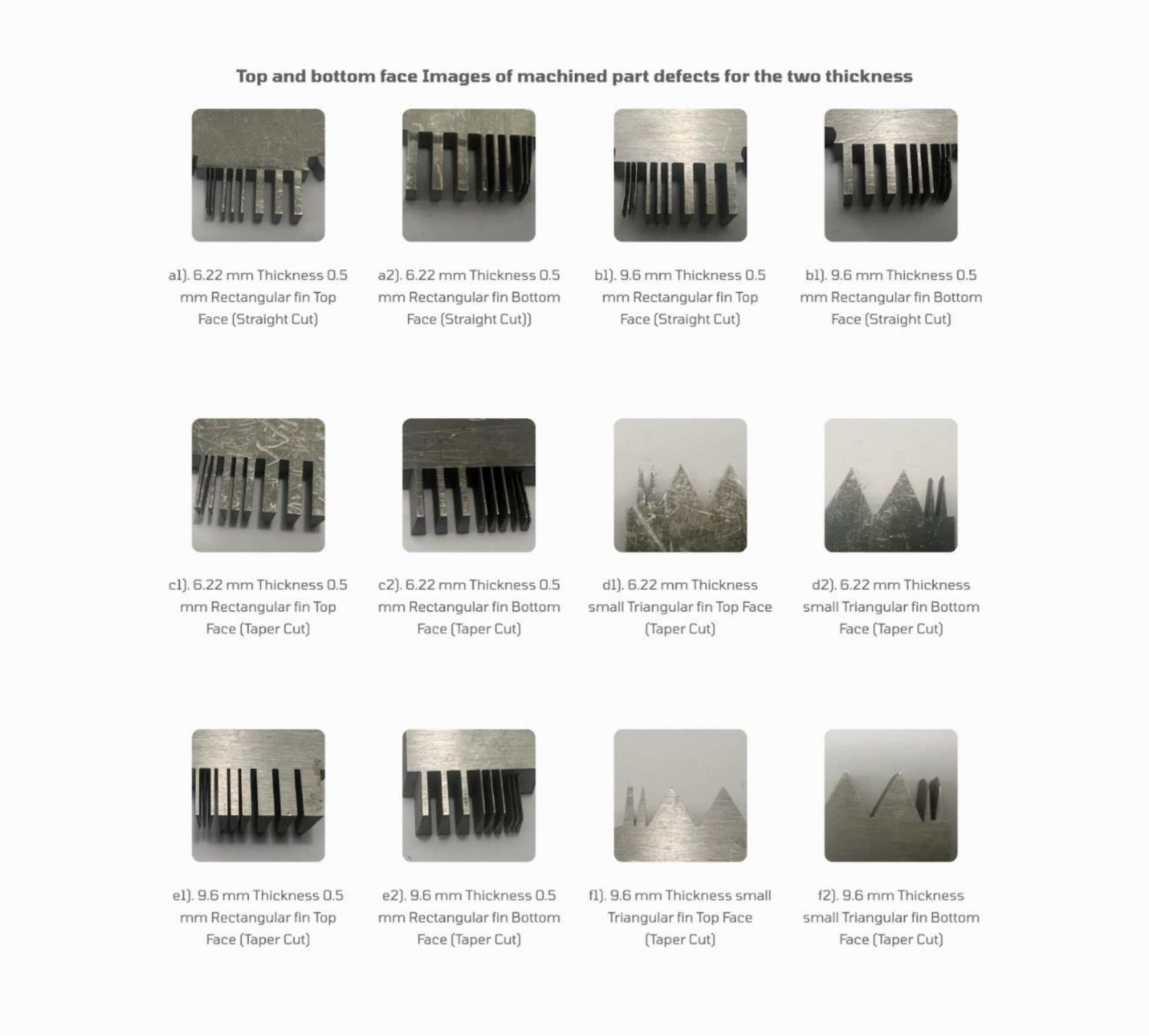

Figure S4. Top and bottom Images of machined parts defect for 6.22 mm and 9.6 mm thickness

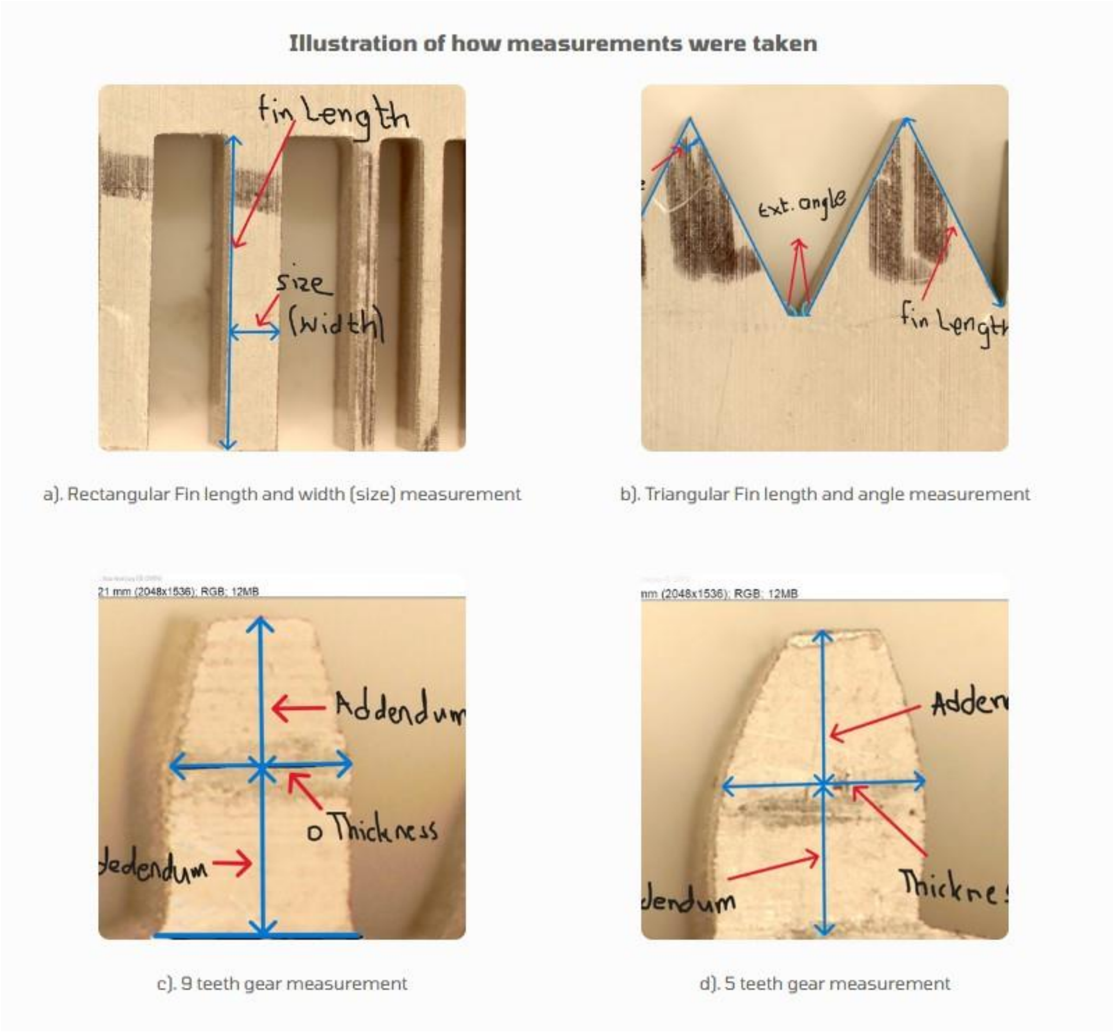

Figure S5. Illustration of how measurements were taken 6.22 mm and 9.6 mm thickness.

Table S42. Measured Dimensions of Rectangular Fins (9.6 mm Thickness, Straight Cut)

Measured Dimensions of Rectangular Fins (9.6 mm Thickness, Straight Cut)								
**Fin size (mm)**	**Fin Number**	**Actual/Intended (mm)**	**Measured Top Face (mm)**	**Measured Bottom Face (mm)**	**Deviation (mm) - (Based on top face only)**	**Average Deviation (mm)**	**Mean (mm)**	**Standard Deviation (mm)**
2	1	2	1.7	1.6	-0.3	-0.366	1.633	0.057
	2	2	1.6	1.6	-0.4			
	3	2	1.6	1.6	-0.4			
1	1	1	0.6	0.6	-0.4	-0.366	0.633	0.057
	2	1	0.6	0.6	-0.4			
	3	1	0.7	0.6	-0.3			
0.5	1	0.5	0.2	0.2	-0.3	-0.3	0.2	0
	2	0.5	0.2	0.2	-0.3			
	3	0.5	0.2	0.2	-0.3			

Table S43. Measured Dimensions of Rectangular Fins Length (9.6 mm Thickness, Straight Cut)

Measured Dimensions of Rectangular Fins (9.6 mm Thickness, Straight Cut)								
**Fin length (mm)**	**Fin Number**	**Actual/Intended (mm)**	**Measured Top Face (mm)**	**Measured Bottom Face (mm)**	**Deviation (mm) - (Based on top face only)**	**Average Deviation (mm)**	**Mean (mm)**	**Standard Deviation (mm)**
2	1	10	9.7	9.6	-0.3	-0.3	9.7	0
	2	10	9.7	9.7	-0.3			
	3	10	9.7	9.7	-0.3			
1	1	10	9.7	9.7	-0.3	-0.3	9.7	0
	2	10	9.7	9.7	-0.3			

	3	10	9.7	9.7	-0.3			
	1	10	8.3	3.1	-1.7			
	2	10	8.8	2.9	-1.2			
0.5	3	10	9.2	3.6	-0.8	-1.233	8.766	0.450

Table S44. Measured Dimensions of Rectangular Fins (6.22 mm Thickness, Straight Cut)

Measured Dimensions of Rectangular Fins (6.22 mm Thickness, Straight Cut)								
**Fin size (mm)**	**Fin Number**	**Actual/Intended (mm)**	**Measured Top Face (mm)**	**Measured Bottom Face (mm)**	**Deviation (mm) - (Based on top face only)**	**Average Deviation (mm)**	**Mean (mm)**	**Standard Deviation (mm)**
	1	2	1.6	1.6	-0.4			
	2	2	1.6	1.6	-0.4			
2	3	2	1.6	1.6	-0.4	-0.4	1.6	0
	1	1	0.6	0.6	-0.4			
	2	1	0.6	0.6	-0.4			
1	3	1	0.6	0.6	-0.4	-0.4	0.6	0
	1	0.5	0.2	0.1	-0.3			
	2	0.5	0.2	0.2	-0.3			
0.5	3	0.5	0.2	0.2	-0.3	-0.3	0.2	0

Table S45. Measured Dimensions of Rectangular Fins Length (6.22 mm Thickness, Straight Cut)

Measured Dimensions of Rectangular Fins (6.22 mm Thickness, Straight Cut)								
**Fin length (mm)**	**Fin Number**	**Actual/Intended (mm)**	**Measured Top Face (mm)**	**Measured Bottom Face (mm)**	**Deviation (mm) - (Based on top face only)**	**Average Deviation (mm)**	**Mean (mm)**	**Standard Deviation (mm)**
2	1	10	9.8	9.8	-0.2	-0.133	9.866	0.057
	2	10	9.9	9.8	-0.1			
	3	10	9.9	9.8	-0.1			
1	1	10	9.8	9.8	-0.2	-0.2	9.8	0
	2	10	9.8	9.8	-0.2			
	3	10	9.8	9.8	-0.2			
0.5	1	10	9.8	8.3	-0.2	-0.266	9.733	0.057
	2	10	9.7	8.4	-0.3			
	3	10	9.7	7.9	-0.3			

Table S46. Measured Dimensions of Triangular Fins Side Length (9.6 mm Thickness, Straight Cut)

Measured Dimensions of Triangular Fins (9.6 mm Thickness, Straight Cut)								
**Fin side length (mm)**	**Fin Number**	**Actual/Intended (mm)**	**Measured Top Face (mm)**	**Measured Bottom Face (mm)**	**Deviation (mm) - (Based on top face only)**	**Average Deviation (mm)**	**Mean (mm)**	**Standard Deviation (mm)**
10	1	10	9	9	-1	-1	9	0
	2	10	9	9	-1			
11.18	1	11.18	10.5	10.4	-0.68	-0.43	10.75	0.353
	2	11.18	11	11	-0.18			

Table S47. Measured Dimensions of Triangular Fins Side Length (6.22 mm Thickness, Straight Cut)

Measured Dimensions of Triangular Fins (6.22 mm Thickness, Straight Cut)								
**Fin side length (mm)**	**Fin Number**	**Actual/Intended (mm)**	**Measured Top Face (mm)**	**Measured Bottom Face (mm)**	**Deviation (mm) - (Based on Bottom face only)**	**Average Deviation (mm)**	**Mean (mm)**	**Standard Deviation (mm)**
10	1	10	8.9	9.2	-0.8	-0.85	9.15	0.070
	2	10	8.9	9.1	-0.9			
11.18	1	11.18	10.6	10.5	-0.68	-0.38	10.8	0.424
	2	11.18	11	11.1	-0.08			

Table S48. Measured Internal and External Angles (9.6 mm Thickness, Straight Cut)

Measured Internal and External Angles (9.6 mm Thickness, Straight Cut)							
**Angles (°)**	**Actual/ Intended (°)**	**Measured Top Face (mm)**	**Measured Bottom Face (mm)**	**Deviation (mm) - (Based on Bottom face only)**	**Average Deviation (mm)**	**Mean (mm)**	**Standard Deviation (mm)**
Internal - Small triangular fin 1	10	10.23	10.237	0.237	0.1375	10.187	0.070
Internal - Small triangular fin 2	10.1	9.878	10.138	0.038			
Internal - Big triangular fin 1	53.2	53.828	52.776	-0.424	-0.256	52.894	0.166
Internal - Big triangular fin 2	53.1	53.251	53.012	-0.088			
External 1	116.6	117.509	117.364	0.764	0.021	116.621	1.050
External 2	116.6	115.759	115.878	-0.722			

Table S49. Measured Internal and External Angles (6.22 mm Thickness, Straight Cut)

Measured Internal and External Angles (6.22 mm Thickness, Straight Cut)							
**Angles (°)**	**Actual/ Intended (°)**	**Measured Top Face (mm)**	**Measured Bottom Face (mm)**	**Deviation (mm) (Based on Bottom face only)**	**Average Deviation (mm)**	**Mean (mm)**	**Standard Deviation (mm)**
Internal - Small triangular fin 1	10	10.635	10.216	0.216			
Internal - Small triangular fin 2	10.1	11.345	10.381	0.281	0.2485	10.298	0.116
Internal - Big triangular fin 1	53.2	53.536	53.373	0.173			
Internal - Big triangular fin 2	53.1	53.207	53.106	0.006	0.089	53.239	0.188
External 1	116.6	117.744	116.285	-0.315			
External 2	116.6	116.501	116.675	0.075	-0.12	116.48	0.275

Table S50. Measured Dimensions of 9 tooth gear Profile (9.6 mm Thickness, Straight Cut)

Measured Dimensions of 9 tooth gear Profile (9.6 mm Thickness, Straight Cut)							
**Profile type (mm)**	**Actual/ Intended (mm)**	**Measured Top Face (mm)**	**Measured Bottom Face (mm)**	**Deviation (mm) -(Based on top face only)**	**Average Deviation (mm)**	**Mean (mm)**	**Standard Deviation (mm)**
Addendum	1.288	1.3	1.3	0.012	NA	NA	NA
Dedendum	1.508	1.5	1.5	-0.008	NA	NA	NA
Tooth thickness/width	1.631	1.6	1.6	-0.031	NA	NA	NA

Table S51. Measured Dimensions of 5 tooth gear Profile (9.6 mm Thickness, Straight Cut)

Measured Dimensions of 5 tooth gear Profile (9.6 mm Thickness, Straight Cut)							
**Profile type (mm)**	**Actual/ Intended (mm)**	**Measured Top Face (mm)**	**Measured Bottom Face (mm)**	**Deviation (mm) - (Based on top face only)**	**Average Deviation (mm)**	**Mean (mm)**	**Standard Deviation (mm)**
Addendum	1.851	1.8	1.8	-0.051	NA	NA	NA
Dedendum	1.782	1.8	1.7	0.018	NA	NA	NA
Tooth thickness/width	2.407	2.4	2.5	-0.007	NA	NA	NA

Table S52. Measured Dimensions of 9 tooth gear Profile (6.22 mm Thickness, Straight Cut)

Measured Dimensions of 9 tooth gear Profile (6.22 mm Thickness, Straight Cut)							
**Profile type (mm)**	**Actual/ Intended (mm)**	**Measured Top Face (mm)**	**Measured Bottom Face (mm)**	**Deviation (mm) - (Based on top face only)**	**Average Deviation (mm)**	**Mean (mm)**	**Standard Deviation (mm)**
Addendum	1.288	1.3	1.3	0.012	NA	NA	NA
Dedendum	1.508	1.5	1.5	-0.008	NA	NA	NA
Tooth thickness/width	1.631	1.6	1.5	-0.031	NA	NA	NA

Table S53. Measured Dimensions of 5 tooth gear Profile (6.22 mm Thickness, Straight Cut)

Measured Dimensions of 5 tooth gear Profile (6.22 mm Thickness, Straight Cut)							
**Profile type (mm)**	**Actual/ Intended (mm)**	**Measured Top Face (mm)**	**Measured Bottom Face (mm)**	**Deviation (mm) - (Based on top face only)**	**Average Deviation (mm)**	**Mean (mm)**	**Standard Deviation (mm)**
Addendum	1.851	1.9	1.9	0.049	NA	NA	NA
Dedendum	1.782	1.8	1.8	0.018	NA	NA	NA
Tooth thickness/width	2.407	2.4	2.3	-0.007	NA	NA	NA

Table S54. Measured Dimensions of Rectangular Fins (9.6 mm Thickness, 5° Taper Cut)

Measured Dimensions of Rectangular Fins (9.6 mm Thickness, 5° Taper Cut)									
**Fin size (mm)**	**Fin Number**	**Measured Top Face (mm)**	**Measured Bottom Face (mm)**	**Calculated Taper Angle (rad)**	**Calculated Taper Angle (°)**	**Deviation (mm)**	**Average Deviation (mm)**	**Mean (mm)**	**Standard Deviation (mm)**
	1	3.4	1.1	0.119	6.831	1.831			
	2	2.8	1.2	0.083	4.763	-0.236			
2	3	2.9	1.2	0.088	5.059	0.059	0.551	5.551	1.117
	1	1.6	0.2	0.072	4.170	-0.829			
	2	1.4	0.2	0.062	3.576	-1.423			
1	3	1.6	0.2	0.072	4.170	-0.829	-1.027	3.972	0.343
	1								
	2								
0.5	3	Part defective. Measurement not taken	Part defective. Measurement not taken	NA	NA	NA	NA	NA	NA

Table S55. Measured Dimensions of Rectangular Fins (6.22 mm Thickness, 5° Taper Cut)

Measured Dimensions of Rectangular Fins (6.22 mm Thickness, 5° Taper Cut)									
**Fin size (mm)**	**Fin Number**	**Measured Top Face (mm)**	**Measured Bottom Face (mm)**	**Calculated Taper Angle (rad)**	**Calculated Taper Angle (°)**	**Deviation (mm)**	**Average Deviation (mm)**	**Mean (mm)**	**Standard Deviation (mm)**
2	1	2.7	1.2	0.119	6.875	1.875	0.660	5.660	1.052
	2	2.3	1.2	0.088	5.053	0.053			
	3	2.3	1.2	0.088	5.053	0.053			
1	1	1.3	0.2	0.088	5.053	0.053	0.053	5.053	0
	2	1.3	0.2	0.088	5.053	0.053			
	3	1.3	0.2	0.088	5.053	0.053			
0.5	1	Part defective. Measurement not taken	Part defective. Measurement not taken	NA	NA	NA	NA	NA	NA
